# Pyrrospirone Z and related decahydrofluorene alkaloids produced by the new coprophilous species *Schizothecium keniense* (*Schizotheciaceae*, *Sordariales*)

**DOI:** 10.3897/mycokeys.136.191424

**Published:** 2026-07-07

**Authors:** Manuela Agudelo-Restrepo, Patrick Dibouloul, Karen Harms, Joseph Tchamgoue, Marc Stadler, Josphat Matasyoh, Simeon Fogue Kouam, Yasmina Marin-Felix

**Affiliations:** 1 Department of Microbial Drugs, Helmholtz Centre for Infection Research GmbH (HZI), and German Centre for Infection Research Association (DZIF), Inhoffenstraße 7, 38124 Braunschweig, Germany Institute of Microbiology, Technische Universität Braunschweig Braunschweig Germany; 2 Institute of Microbiology, Technische Universität Braunschweig, Spielmannstraße 7, 38106 Braunschweig, Germany Department of Chemistry, Higher Teacher Training College, University of Yaounde 1 Yaounde Cameroon; 3 Department of Chemistry, Higher Teacher Training College, University of Yaounde 1, P.O. Box 47, Yaounde, Cameroon Department of Organic Chemistry, Faculty of Science, University of Yaounde 1 Yaounde Cameroon; 4 Department of Organic Chemistry, Faculty of Science, University of Yaounde 1, P.O. Box 812, Yaounde, Cameroon Department of Microbial Drugs, Helmholtz Centre for Infection Research GmbH (HZI), and German Centre for Infection Research Association (DZIF) Braunschweig Germany; 5 Department of Chemistry, Egerton University, P.O. Box 536, Egerton 20115, Kenya Department of Chemistry, Egerton University Egerton Kenya

**Keywords:** Antimicrobial activity, coprophilous fungi, cytotoxicity, secondary metabolites, Sordariomycetes

## Abstract

A decahydrofluorene-class alkaloid, pyrrospirone Z (**1a**), along with pyrrospirone F (**2**), pyrrospirone M (**3**), GKK1032A2 (**4**), daldinol (**5**), orthosporin (**6**), and 3,4-dihydro-3,4,8-trihydroxy-1(2H)-naphthalenone (**7**), was purified from the new coprophilous species *Schizothecium keniense*, isolated from dung in Kenya. Structural elucidation was achieved using comprehensive 1D and 2D NMR spectroscopy, and high-resolution electrospray ionization mass spectrometry (HR-ESI-MS), with relative configurations assigned based on NOESY correlations. Among the compounds, pyrrospirone F (**2**) and GKK1032A2 (**4**) displayed significant antibacterial activity against the Gram-positive bacteria *Bacillus subtilis* (MIC = 4.2 μg/mL and 8.3 μg/mL, respectively) and *Staphylococcus aureus* (MIC = 8.3 μg/mL for both). Additionally, compounds **2** and **4** showed mild antifungal activity against the filamentous fungi *Mucor hiemalis* at 33.3 μg/mL. Cytotoxicity assays revealed that GKK1032A2 (**4**) was the most potent compound, with IC_50_ values ranging from 2.0 µM to 8.1 µM across the tested human cancer cell lines. This work represents the first investigation of the chemical constituents of the genus *Schizothecium* and underscores the potential of coprophilous fungi as valuable sources of biologically active metabolites.

## Introduction

Fungi are among the most diverse and ubiquitous groups of eukaryotic organisms. They play essential roles as decomposers, mutualists, and parasites across a wide range of habitats, including soil, marine environments and plants, as well as more specialized niches (Case et al. 2025). One such niche is mammalian dung, a nutrient-rich but temporally limited substrate colonized by fungi known as coprophilous (Richardson 2001). These fungi have adapted to this ephemeral environment, while simultaneously facing intense competition from other dung colonizers such as bacteria, protists, invertebrates, and other fungi, in addition to the selective pressures imposed by passage through the mammalian digestive tract (Bills et al. 2013). These ecological challenges are thought to favor the production of distinctive secondary metabolites, making coprophilous fungi a promising source of novel antibiotics and other bioactive compounds. Nevertheless, these coprophilous communities remain relatively unexplored compared with other fungal groups, likely because standard isolation methods predominantly recover ubiquitous fungi that quickly overgrow rather than more unusual species (Charria-Girón et al. 2025).

Within this ecological framework, members of the order *Sordariales* are frequently associated with dung, and several taxa have emerged as prolific producers of bioactive secondary metabolites with therapeutic potential (Charria-Girón et al. 2022). For instance, the coprophilous fungus *Rhypophila pleiospora* (syn: *Podospora pleiospora*), isolated from rabbit dung, produces sordarin and three related sordarin-type metabolites, hydroxysordarin, sordaricin and sordarin B. In that study, sordarin and sordarin B showed minimum inhibitory concentrations of 0.5–2.5 µg/mL against the phytopathogenic yeast *Eremothecium coryli* (syn. *Nematospora coryli*) and against *Sporobolomyces roseus*, an environmental, plant-associated yeast (Weber et al. 2005). Despite these findings, many taxa within the *Sordariales* remain poorly studied, with entire families yet to be investigated in terms of their chemical diversity (Charria-Girón et al. 2025).

As part of our ongoing search for novel fungal metabolites in the order *Sordariales*, we investigated a *Schizothecium* strain isolated from cow dung. *Schizothecium* is a genus in *Schizotheciaceae*, which was first described by Corda (1838). This family is characterized by ornate, ostiolate ascomata, whose structures differ among genera (Marin-Felix et al. 2020). To date, no chemical investigations have been reported for this genus. In the present study, we describe the isolation of a new pyrrospirone alkaloid, pyrrospirone Z (**1a**), together with six known metabolites (**2**–**7**), from the crude extract of the new species, *Schizothecium keniense*. Their antifungal, antibacterial and cytotoxic activities were evaluated, and the structural elucidation and biological data are presented herein.

## Experimental section

### Fungal isolation

Dung samples from various herbivorous animals, such as cow and goat, were collected on the campus of Egerton University, Njoro, Kenya, between late November and early December 2021 and incubated in moist chambers by placing the dung on moist filter papers in petri dishes at room temperature of 23 °C (Richardson 2001). Samples were examined every few days for 2 months using a Zeiss Stemi 508 stereomicroscope (Jena, Germany). Spores of coprophilous fungi from mature ascomata were collected with a sterile needle and transferred to petri dishes containing yeast malt agar (YM agar; malt extract 10 g/L, yeast extract 4 g/L, D-glucose 4 g/L, agar 20 g/L, pH 6.3) before autoclaving (Rupcic et al. 2018). To inhibit bacterial growth, 250 mg/mL of penicillin and streptomycin, and 50 mg/L of ivermectin were added to the medium to prevent mites and nematodes. Plates were incubated at room temperature until mycelial growth was observed.

### Morphological characterization

The strain was incubated for 14 days in oatmeal agar (OA; Sigma-Aldrich, St. Louis, MO, USA), potato carrot agar (PCA; HiMedia, Mumbai, India), potato dextrose agar (PDA; HiMedia, Mumbai, India) and Czapek-Dox agar (Cz; HiMedia, Mumbai, India) at 25 °C, for the culture characterization as previously reported by Marin-Felix et al. 2020. Color notations in parentheses were taken according to the Royal Horticultural Society (1996). The strain was cultured at 5, 8, 10, 15, 25, 28, 30, 35, and 40 °C to determine the cardinal temperatures for growth. Fungal structures were mounted and measured in lactic acid (Marin-Felix et al. 2020) with a Nikon Eclipse Ni compound microscope, using a DS-Fi3 digital camera (Nikon, Tokyo, Japan) and NIS-Elements imaging software v. 5.20. A Keyence (Neu-Isenburg, Germany) VHX-970F microscope was used to obtain photomicrographs.

### DNA isolation, amplification and phylogenetic study

DNA isolation and amplification of the internal transcribed spacer (ITS) regions and the large subunit (LSU) of the nuclear ribosomal RNA (rRNA) gene complex and partial fragments of the second largest subunit of DNA-directed RNA polymerase II (*rpb2*) and β-tubulin (*tub2*) genes were performed following the methodology described in Harms et al. (2021).

Phylogenetic analyses were conducted based on a concatenated dataset of the four loci from our isolate and from type and reference strains of taxa belonging to the family *Schizotheciaceae*, using *Naviculispora terrestris*CBS 137295 and *Pseudorhypophila mangenotii*CBS 419.67 as outgroups (Table 1). The procedures for phylogenetic analyses were performed following the methodology described in Harms et al. (2021). The sequences generated in this study were deposited in GenBank (Table 1), and the final alignment is provided in the Supp. material 1: table SS1.

**Table 1.** Isolates and reference strains of the family *Schizotheciaceae* included in the phylogenetic study. New species introduced in the present study in ***bold and italics***. Sequences generated in this study in **bold**.

**Taxa**	**Strain**	**GenBank accession #**				**References**
		**LSU**	**ITS**	***rpb2***	***tub2***	
Apiosordaria microcarpa*	CBS 692.82^T^	MK926841	MK926841	MK876803	-	Wang et al. (2019)
*Apodus deciduus*	CBS 506.70^T^	AY681165	AY681199			Cai et al. (2006)
Arnium cirriferum*	CBS 120041	KF557673	-	-	KF557709	Kruys et al. (2015)
Cercophora newfieldiana*	SMH 3303	AY780062	-	AY780167	AY780106	Miller and Huhndorf (2005)
Cercophora thailandica*	MFLUCC 12-0845^T^	KU863127	KU940139	KU940176	-	Dai et al. (2017)
*Echria gigantospora*	F77-1	KF557674	-	-	KF557710	Kruys et al. (2015)
*Echria macrotheca*	Lundqvist 2311	KF557684	-	-	KF557715	Kruys et al. (2015)
*Garciamycella chlamydospora*	CBS 150388^T^	PX270288	PX270286	PX275625	PX275627	Agudelo-Restrepo et al. (2026)
*Garciamycella cyclaminis*	CBS 166.42^T^	MH867608	HQ713776	PX275626	-	Grünig et al. (2011); Vu et al. (2019); Agudelo-Restrepo et al. (2026)
	CBS 120402	KP981429	PX270287	KP981611	KP981556	Marin-Felix et al. (2020); Agudelo-Restrepo et al. (2026)
*Garciamycella fici*	MFLUCC 20-0160^T^	MW114439	MW114387	-	-	Tennakoon et al. (2021)
*Immersiella caudata*	SMH 3298	AY436407	-	AY780161	AY780101	Miller and Huhndorf (2004, 2005)
*Immersiella hirta*	E00204950	KF557675	-	-	KF557711	Kruys et al. (2015)
	E00204487	KF557676	-	-	KF557712	Kruys et al. (2015)
*Immersiella immersa*	SMH 4104	AY436409	-	AY780181	AY780123	Miller and Huhndorf (2004, 2005)
	SMH 2589	AY436408	-	-	-	Miller and Huhndorf (2004)
*Jugulospora antarctica*	IMI 381338^T^	KP981433	-	KP981616	KP981561	Marin-Felix et al. (2020)
*Jugulospora carbonaria*	ATCC 34567	AY346302	-	AY780196	AY780141	Huhndorf et al. (2004), Miller and Huhndorf (2005)
*Jugulospora rotula*	ATCC 38359	AY346287	-	AY780178	AY780120	Huhndorf et al. (2004); Miller and Huhndorf (2005)
*Jugulospora vestita*	CBS 135.91^T^	MT785872	MT784135	MT783824	MT783825	Marin-Felix et al. (2020)
*Lundqvistomyces karachiensis*	CBS 657.74	KP981447	MK926850	KP981630	KP981478	Wang et al. (2019); Marin-Felix et al. (2020)
*Lundqvistomyces tanzaniensis*	TRTC 51981^T^	AY780081	MH862260	AY780197	AY780143	Miller and Huhndorf (2005); Vu et al. (2019)
*Morinagamyces vermicularis*	CBS 303.81^T^	KP981427	MT904879	KP981609	KP981554	Harms et al. (2021)
*Naviculispora terrestris*	CBS 137295^T^	KP981439	MT784136	KP981622	KP981567	Marin-Felix et al. (2020)
Podospora bullata*	CBS 115576^T^	MH874548	DQ166960	-	-	Bell et al. (2016); Vu et al. (2019)
Podospora communis*	CBS 118393	MH874584	MH863031	-	-	Vu et al. (2019)
*Podospora cupiformis*	CBS 246.71^T^	AY999102	AY999125	-	-	Cai et al. (2005)
Podospora excentrica*	CBS 118392	MH874583	MH863030	-	-	Vu et al. (2019)
Podospora intestinacea*	CBS 113106	AY999104	AY999121	-	-	Cai et al. (2005)
Podospora prethopodalis*	CBS 121128	MH874659	MH863103	-	-	Vu et al. (2019)
Podospora serotina*	CBS 252.71	MH871878	MH860102			Vu et al. (2019)
*Pseudoechria curvicolla*	NBRC 8548	AY999099	AY999122	-	-	Cai et al. (2005)
	CBS 259.69	MH871036	MH859302	-	-	Vu et al. (2019)
*Pseudoechria decidua*	CBS 254.71^T^	MK926842	MK926842	MK876804	-	Wang et al. (2019)
*Pseudoechria longicollis*	CBS 368.52^T^	MK926847	MK926847	MK876809	-	Wang et al. (2019)
*Pseudoechria prolifica*	CBS 250.71^T^	MK926848	MK926848	MK876810	-	Wang et al. (2019)
*Pseudorhypophila mangenotii*	CBS 419.67^T^	KP981444	MT784143	KP981627	KP981571	Marin-Felix et al. (2020)
*Pseudoschizothecium atropurpureum*	SMH 2961	AY780056	-	-	AY780099	Miller and Huhndorf (2005)
	SMH 3073	AY780057	-	AY780160	AY780100	Miller and Huhndorf (2005)
*Ramophialophora globispora*	CGMCC 3.17940	KU746745	KU746699	KY883252	-	Zhang et al. (2017, 2018)
*Rinaldiella pentagonospora*	CBS 132344^T^	KP981442	MH866007	KP981625	KP981570	Vu et al. (2019); Marin-Felix et al. (2020)
*Schizothecium aloides*	CBS 879.72	AY999097	AY999120	-	-	Cai et al. (2005)
*Schizothecium carpinicola*	CBS 228.87^T^	AY999095	AY999118	-	-	Cai et al. (2005)
*Schizothecium conicum*	CBS 434.50	MH868218	MH856702	-	-	Vu et al. (2019)
*Schizothecium curvisporum*	CBS 507.50	AY999096	AY999119	-	-	Cai et al. (2005)
	ATCC 36709	AY346300	-	AY780192	AY780136	Huhndorf et al. (2004); Miller and Huhndorf (2005)
*Schizothecium curvuloides*	CBS 129.94	-	AY515357	-	-	Debuchy, Bell and Mahoney (Data unpubl.)
*Schizothecium dactylidis*	KUNCC 25-19192^T^	PV607496	PV608640	PV631997	-	Gao et al. (2025)
*Schizothecium dakotense*	CBS 130.94	-	AY515358	-	-	Debuchy, Bell and Mahoney (Data unpubl.)
*Schizothecium fimbriatum*	CBS 144.54	AY780075	AY999115	AY780189	AY780132	Cai et al. (2005); Miller and Huhndorf (2005)
*Schizothecium glutinans*	CBS 134.83	AY999093	AY999116	-	-	Cai et al. (2005)
*Schizothecium inaequale*	CBS 356.49^T^	MK926846	MK926846	MK876808	-	Wang et al. (2019)
***Schizothecium keniense***	**CCF 6931^T^**	**PZ195604**	**PZ195603**	**PZ195162**	**PZ195163**	**Present study**
*Schizothecium minicauda*	CBS 227.87	MH873757	MH862068	-	-	Vu et al. (2019)
*Schizothecium miniglutinans*	CBS 131.94	-	AY515362	-	-	Debuchy, Bell and Mahoney (Data unpubl.)
*Schizothecium selenosporum*	CBS 109403^T^	MK926849	MK926849	MK876811	-	Wang et al. (2019)
*Schizothecium tetrasporum*	CBS 394.87	MH873776	MH862087			Vu et al. (2019)
*Schizothecium vesticola*	SMH 3187	AY780076	-	-	-	Miller and Huhndorf (2005)
Zopfiella erostrata*	CBS 255.71	AY999110	AY999133	-	-	Cai et al. (2005)
*Zygopleurage zygospora*	SMH 4219	AY346306	-	-	AY780147	Huhndorf et al. (2004); Miller and Huhndorf (2005)

ATCC: American Type Culture Collection, Virginia, USA; CBS: Westerdijk Fungal Biodiversity Institute, Utrecht, the Netherlands; CCF: Culture Collection of Fungi, Prague, Czech Republic; CGMCC: Chinese General Microbiological Culture Collection Center, Beijing, China; IMI: International Mycological Institute, CABI-Bioscience, Egham, UK; KUNCC: Kunming Institute of Botany Culture Collection, Kunming, China; MFLUCC: Mae Fah Luang University Culture Collection, Chiang Ria, Thailand; NBRC: Biological Resource Center, Chiba, Japan; TRTC: Royal Ontario Museum, Toronto, Canada; Lundqvist, SMH: personal collections of Nils Lundqvist, Sabine M. Huhndorf, respectively. ^T^ indicates ex-type strains, respectively. *Taxa with generic names applied in the broad sense (sensu lato), not necessarily reflecting molecular phylogenetic relationships

### Fermentation, extraction, and isolation of metabolites

The strain *Schizothecium keniense* was evaluated for secondary metabolite production in solid medium. First, the strain was grown in yeast malt agar (YM: malt extract 10 g/L, yeast extract 4 g/L, D-glucose 4 g/L, agar 20 g/L, pH 6.3, before autoclaving) at 23 °C for 7 days. Then 8 small pieces (1 cm diam.) were transferred in 200 mL of liquid YM, and it was fermented at 23 °C and 140 rpm over 7 days. 6 mL of this seed culture were transferred to 30 flasks containing 200 mL of solid rice medium (BRFT, per flask: brown rice 28 g and 0.1 L of base liquid composed of yeast extract 1 g/L, di-sodium tartrate di-hydrate 0.5 g/L, KH_2_PO_4_ 0.5 g/L) and the cultures were fermented for 14 days at 23 °C in darkness.

To obtain the crude extract, the fermented rice was first extracted twice by adding acetone and sonicating the mixture in an ultrasonic bath at 40 °C for 30 minutes. Consequently, the extract was separated from the mycelia using a cellulose filter paper (MN 615 1/4 Ø 185 mm, Macherey-Nagel GmbH & Co. KG, Düren, Germany) and the acetone was evaporated under reduced pressure at 40 °C using a rotary evaporator (Heidolph Instruments GmbH & Co. KG, Germany) connected to a vacuum pump (Vacuubrand GmbH & Co. KG, Wertheim am Main, Germany). This acetone phase was extracted twice with a 1:1 ratio of ethyl acetate (EtOAc) using a separatory funnel. The resulting EtOAc extract was evaporated to dryness under vacuum at 40 °C, yielding a final mass of 2.23 g.

For metabolites isolation, the crude extract was pre-fractionated by flash chromatography (Grace Reveleris, Columbia, MD, USA) using a 25 g silica cartridge. The mobile phases consisted of A (DCM), B (DCM/MeOH 9:1), and C (MeOH), with a flow rate of 30 mL/min. The gradient was as follows: 100% A for 8 min; increased to 50% B for 20 min, and held for 2 min; further increased to 100% B for 10 min; followed by a mixture of A and C, reaching 10% C in 3.3 min; then increased to 100% C for over 10.5 minutes; and finally holding at 100% C for 15 min.

Subsequently, compounds **1a** (0.47 mg, t_R_ = 2.05 min), **2** (3.75 mg, t_R_ = 12.64 min), and **3** (2.31 mg, t_R_ = 10.27 min) were obtained after combining fractions 7 and 9, yielding a total mass of 150 mg. The mixture was separated using a PLC 2250 preparative HPLC system (Gilson, Middleton, WI, USA) equipped with a Gemini 10u C18 110Å column (250 × 50 mm, 10 μm; Phenomenex, Torrance, CA, USA) as the stationary phase and in the following conditions: solvent A: H_2_O + 0.1% formic acid; solvent B: ACN + 0.1% formic acid; flow rate, 40 mL/min. The gradient program consisted of 5% B to 40% B in 5 minutes, followed by a gradual increase from 40% B to 80% B in 55 min, reaching 100% within 5 min.

Fraction 6 (120 mg) led to the isolation of compounds **6** (1.15 mg, t_R_ = 4.98 min) and **7** (1.64 mg, t_R_ = 2.24 min) using a Luna column (250 × 50 mm, 10 μm, 100 g). The mobile phase consisted of solvent A (H_2_O + 0.1% formic acid) and B (ACN + 0.1% formic acid), with a flow rate of 40 mL/min and a gradient from 5% B to 100% B for 75 minutes. This separation also yielded compound **4** (1.64 mg, t_R_ = 13.38 min), which required further purification using an Agilent Technologies 1200 Infinity Series semipreparative reverse-phase HPLC system equipped with an XBridge BEH C18 column (250 × 10 mm, 5 μm, Waters). The mobile phase consisted of solvent A (H_2_O + 0.1% formic acid) and solvent B (ACN + 0.1% formic acid), with a flow rate of 3 mL/min under isocratic conditions (66.3% ACN + 33.7% H_2_O.

Finally, fractions 1 and 2 were combined, yielding a total mass of 300 mg. The mixture was separated using a PLC 2250 preparative HPLC system (Gilson, Middleton, WI, USA) equipped with a Gemini 10u C18 110Å column (250 × 50 mm, 10 μm; Phenomenex, Torrance, CA, USA) as the stationary phase. The mobile phase consisted of solvent A (H_2_O + 0.1% formic acid) and solvent B (ACN + 0.1% formic acid), with a flow rate of 40 mL/min. The gradient program was as follows: 5% B to 15% B for 10 min, then an increase from 15% B to 65% B for 50 min, and finally reaching 100% within 10 min. This allowed the purification of compound **5** (1.30 mg, t_R_ = 12.28 min).

Pyrrospirone Z (**1a**): white amorphous solid; UV/Vis (MeOH): *λ*_max_ = 230 nm; HR-(+)ESI-MS: *m/z* 536.2987 [M+H]^+^ (calcd. 536.3007 for C_32_H_42_NO_6_^+^); rt = 10.88 min (HR-ESI-MS) ^1^H and ^13^C NMR data (Acetone-*d_6_*, 700 MHz and 175 MHz), see Table 2.

**Table 2.** ^1^H and ^13^C NMR data of pyrrosporine Z (1a)^g^ and pyrrosporine S (1b)^d^ (Ban et al. 2025).

**No**.	**Pyrrosporine Z (1a)**		**Pyrrosporine S (1b)**	
	***150 MHz***	***700 MHz***	***125 MHz***	***500 MHz***
	***δ*_C_**	***δ*_H_ (*mult, J* in Hz)**	***δ*_C_**	***δ*_H_ (*mult, J* in Hz)**
1	49.4	1.96 (*m*), 0.86 (*m*)	47.7	1.83 (*dd*, 12.5, 3.4), 0.82 (*m*)
2	28.4	1.86 (*m*)	27.4	1.72 (*m*)
3	45.8	1.80 (*dd*, 12.7, 3.8), 0.64 (*m*)	44.9	1.72 (*m*), 0.59 (*q*, 14.1)
4	27.6	1.96 (*m*)	26.7	1.72 (*m*)
5	61.4	1.09 (*m*)	59.6	1.15 (*dd*, 8.2, 3.2)
6	41.4	-	40.5	-
7	54.3	1.85 (*m*)	53.3	1.68 (*brd*, 13.5)
8	48.7	2.73 (*m*)	42.1	3.04 (*ddd*, 12.9, 8.0, 4.6)
9	89.5	4.31 (*dd*, 7.3, 4.4)	88.6	4.09 (*dd*, 7.7, 4.6)
10	136.5	-	55.4	-
11	132.6	5.04 (*d*, 1.9)	65.5	2.40 (*s*)
12	76.3	-	75.6	-
13	58.1	3.95 (*d*, 7.5)	55.8	3.63 (*d*, 8.0)
1*4*	203.0	-	199.8	-
15	58.6	-	58.0	-
16	51.2	2.05 (*m*), 1.34 (*m*)	50.1	2.04 (*d*, 13.1) 1.30 (*m*)
17	88.7	5.71 (*m*)	87.6	5.47 (*d*, 9.5)
18	175.9	-	174.8	-
19	87.0	-	86.1	-
20	44.0	3.08 (*d*, 15.2), 1.52 (*d*, 15.2)	42.9	2.85 (*d*, 15.0) 1.30 (*m*)
21	46.6	3.00 (*d*, 12.8), 2.77 (*m*)	45.5	2.79 (*d*, 12.6) 2.59 (*d*, 12.6)
22	131.0	-	129.6	-
23	132.3	7.12 (*dd*, 8.2, 2.2)	131.5	7.06 (*dd*, 8.1, 2.1)
24	123.3	6.66 (*dd*, 8.2, 2.5)	121.9	6.60 (*dd*, 8.0, 2.4)
25	158.5	-	157.5	-
26	119.2	6.92 (*dd*, 8.4, 2.5)	116.7	6.78 (*dd*, 8.6, 2.5)
27	134.5	7.14 (*dd*, 8.4, 2.2)	133.4	7.01 (*dd*, 8.6, 2.1)
28	22.8	0.91 (*d*, 6.5)	22.7	0.88 (*d*, 6.4)
29	19.7	1.11 (*d*, 6.3)	19.3	1.00 (*d*, 6.0)
30	16.4	1.22 (*s*)	14.8	1.12 (*s*)
31	19.0	1.85 (*s*)	20.8	1.27 (*s*)
32	25.2	1.36 (*s*)	23.8	1.36 (*s*)

Pyrrospirone F (**2**): white to yellow amorphous solid; UV/Vis (MeOH): *λ*_max_ = 230 nm; HR-(+)ESI-MS: *m/z* 520.3036 [M+H]^+^ (calcd. 520.3057 for C_32_H_42_NO_5_^+^); rt = 11.45 min (HR-ESI-MS); ^1^H and ^13^C NMR data (Acetone-*d_6_*, 500 MHz and 125 MHz) (Song et al. 2018a).

Pyrrospirone M (**3**): white to yellow amorphous solid; UV/Vis (MeOH): *λ*_max_ = 230 nm; HR-(+)ESI-MS: *m/z* 552.2928 [M+H]^+^ (calcd. 552.2956 for C_32_H_42_NO_7_^+^); rt = 9.13 min (HR-ESI-MS); ^1^H and ^13^C NMR data (Acetone-*d_6_*, 500 MHz and 125 MHz) (Yao et al. 2022).

GKK1032A2 (**4**): colorless amorphous solid; UV/Vis (MeOH): *λ*_max_ = 234, 260 nm; HR-(+)ESI-MS: *m/z* 504.3083 [M+H]^+^ (calcd. 504.3108 for C_32_H_42_NO_4_^+^); rt = 12.18 min (HR-ESI-MS); ^1^H and ^13^C NMR data (Acetone-*d_6_*, 600 MHz and 150 MHz) (Qi et al. 2019).

Daldinol (**5**): white to yellow amorphous solid; UV/Vis (MeOH): *λ*_max_ = 236, 310 nm; HR-(+)ESI-MS: *m/z* 347.1260 [M+H]^+^ (calcd. 347.1278 for C_22_H_19_O_4_^+^); rt = 11.11 min (HR-ESI-MS); ^1^H and ^13^C NMR data (Acetone-*d_6_*, 500 MHz and 125 MHz) (Hashimoto et al. 1994).

Orthosporin (**6**): light orange oil; UV/Vis (MeOH): *λ*_max_ = 246, 326 nm; HR-(+)ESI-MS: *m/z* 237.0746 [M+H]^+^ (calcd. 237.0757 for C_12_H_13_O_5_^+^); rt = 4.0 min (HR-ESI-MS); ^1^H and ^13^C NMR data (Acetone-*d_6_*, 600 MHz and 150 MHz) (Ichihara et al. 1989).

3,4-dihydro-3,4,8-trihydroxy-1(2H)-naphthalenone (**7**): dark brown oil; UV/Vis (MeOH): *λ*_max_ = 260, 334 nm; HR-(+)ESI-MS: *m/z* 195.0642 [M+H]^+^ (calcd. 195.0652 for C_10_H_11_O_4_^+^); rt = 1.85 min (HR-ESI-MS); ^1^H and ^13^C NMR data (Acetone-*d_6_*, 600 MHz and 150 MHz) (Ayer et al. 1989).

### Chromatography and spectral methods

Crude extracts and pure compounds were dissolved in a 1:1 acetone-methanol mixture and adjusted to final concentrations of 4.5 and 1 mg/mL, respectively. HPLC-DAD-MS analyses were carried out on an amaZon speed ETD ion trap mass spectrometer (Bruker Daltonics, Bremen, Germany) operated in both positive and negative ionization modes, using a C18 Acquity UPLC BEH column (50 × 2.1 mm, 1.7 µm; Waters, MA, USA) connected to a Dionex UltiMate 3000 UHPLC system (Thermo Scientific, Waltham, MA, USA). The chromatographic separation used solvent A (deionized H2O with 0.1% formic acid) and solvent B (acetonitrile with 0.1% formic acid) with the following gradient: 5% B for 0.5 min, linear increase to 100% B over 20.5 min, and isocratic at 100% B for 4.5 min; the flow rate was 0.6 mL/min and UV-Vis signals were recorded from 190 to 600 nm. High-resolution ESI-MS (HR-ESI-MS) data were acquired on a maXis ESI-TOF mass spectrometer (Bruker Daltonics, Bremen, Germany) coupled to an Agilent 1200 Infinity Series HPLC-UV system (Agilent Technologies, Santa Clara, CA, USA), using the same column and gradient conditions as in the HPLC-DAD-MS analysis; additional MS parameters were: scan range 100–2500 m/z, acquisition rate 2 Hz, capillary voltage 4500 V, and drying gas temperature 200 °C.

1D/2D nuclear magnetic resonance (NMR) spectra were measured in Acetone-*d_6_* on an Avance III 700 spectrometer (Bruker, ^1^H NMR: 700 MHz, ^13^C: 175 MHz, Billerica, MA, USA), an Avance III 600 spectrometer (Bruker, ^1^H NMR: 600 MHz, ^13^C: 150 MHz, Billerica, MA, USA), and an Avance III 500 spectrometer (Bruker, ^1^H NMR: 500 MHz, ^13^C: 125 MHz, Billerica, MA, USA), using a 5 mm TXI cryoprobe.

### Biological assays

The cytotoxic and antimicrobial activities of all isolated compounds were tested following the experimental procedures described by Shao et al. (2020), with five additional cell lines, human prostate carcinoma (PC-3), human ovarian cancer (SKOV-3), human breast adenocarcinoma (MCF-7), human epidermoid carcinoma (A431), and human lung carcinoma (A549).

The cytotoxicity of the compounds was evaluated against the mouse fibroblast cell line L929 and the human cancer cell lines: PC-3, SKOV-3, MCF-7, A431, and A549 using the MTT assay to determinate cell viability, and the half-maximum inhibitory concentration (IC_50_) values were calculated from dose-response curves relative to untreated control cells. The minimum inhibitory concentration (MIC) was determined by a serial dilution assay against one filamentous fungus, *Mucor hiemalis* (DSM2656), four yeasts, *Candida albicans* (DSM 1665), *Rhodotorula glutinis* (DSM 10134), *Schizosaccharomyces pombe* (DSM 70572), *Wickerhamomyces anomalus* (DSM 6766), as well as the bacteria *Acinetobacter baumannii* (DSM 30008), *Bacillus subtilis* (DSM 10), *Chromobacterium violaceum* (DSM 30191), *Escherichia coli* (DSM 1116), *Mycolicibacterium smegmatis* (ATCC 700084), *Pseudomonas aeruginosa* PA14 (DSM 19882), and *Staphylococcus aureus* (DSM 346).

## Results and discussion

### Phylogenetic analysis

**Figure 1.**
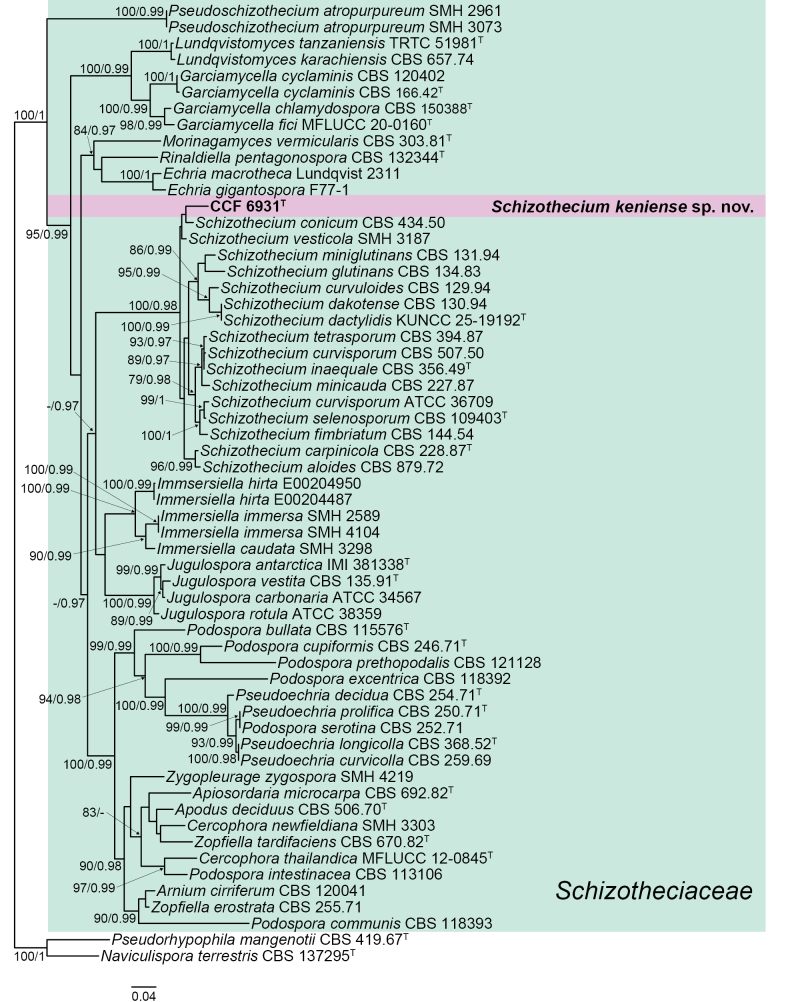
Randomized accelerated maximum likelihood (RAxML) phylogram obtained from the combined sequences of the internal transcribed spacer region (ITS), the nuclear rDNA large subunit (LSU), and fragments of ribosomal polymerase II subunit 2 (*rpb2*) and β-tubulin (*tub2*) genes of type and reference strains belonging to the *Schizotheciaceae*. Bootstrap support values ≥ 70 / Bayesian posterior probability scores ≥ 0.95 are indicated along branches. Branch lengths are proportional to distance. New species proposed in the present study is indicated in **bold**. ^T^ indicates ex-type strains.

The lengths of the individual alignments used in the combined dataset were 631 bp (ITS), 886 bp (LSU), 966 bp (*rpb2*) and 618 bp (*tub2*), and the final total alignment was 3101 bp. The phylogenetic tree obtained from the RAxML analysis of the combined dataset, including bootstrap support and Bayesian posterior probability at the nodes, is shown in Fig. 1. The RAxML tree obtained agreed with the topology of the tree generated by the Bayesian analysis. Our strain was placed in a well-supported clade (100 bs / 0.98 pp) corresponding to the genus *Schizothecium (Schizotheciaceae)*. However, its phylogenetic distance from the other accepted species of the genus was sufficient to support its recognition as a new species, herein introduced as *S. keniense*.

### Taxonomy

#### Schizothecium keniense

Taxon classificationFungiMonhysteridaLasiosphaeriaceae

Y. Marín & K. Harms sp. nov.

9AB48C61-DC5E-59A6-A933-8A799EB5E61C

862939

Fig. 2

##### Etymology.

Refers to the country from which the fungus was isolated.

**Figure 2.**
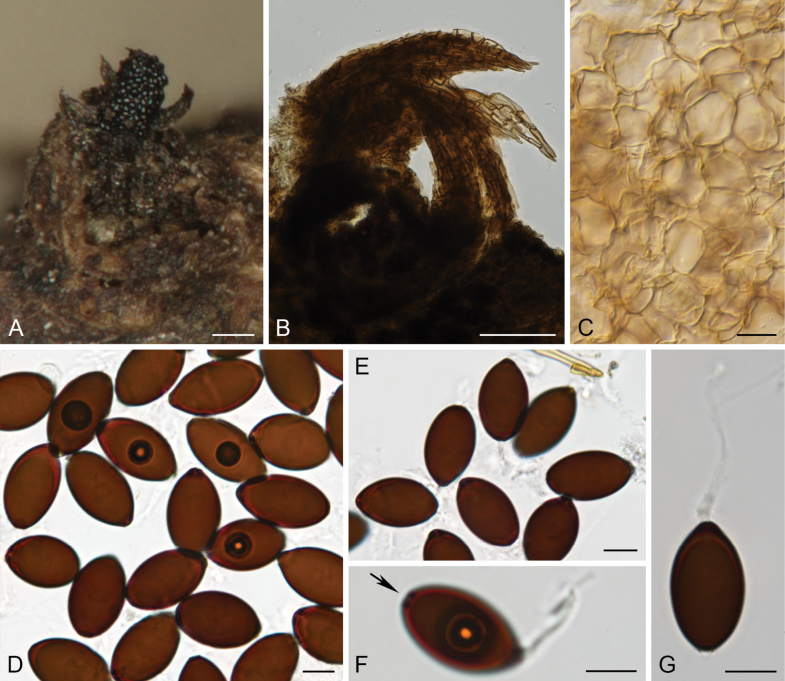
*Schizothecium keniense* (ex-type strain CCF 6931). **A**. Ascoma; **B**. Groups of agglutinated hairs in the upper part of the ascoma; **C**. Ascomatal wall of *textura angularis* to *globulosa*; **D, E**. Ascospores; **F**. Ascospore with upper and lower cell (arrow indicates germ pore of the upper cell); **G**. Ascospore with mucilaginous appendage in the upper cell. Scale bars: 100 µm (**A**); 50 µm (**B**); 10 µm (**C–G**).

##### Description.

Specimen growing on dung. Ascomata ostiolate, immersed, erumpent, scattered in the dung, pyriform, brown to dark brown, translucent, upper part ornate with groups of pale brown to brown swollen agglutinated hairs 149–169 × 42–63 µm (n = 3); ascomatal wall membranaceous, pale brown to brown, *textura angularis* to *textura globulosa*. Paraphyses and asci not observed due to the aged condition of the specimen. Ascospores two-celled; upper cell dark brown, fusiform to obovoid, smooth-walled, guttulate, with a terminal germ pore, 21–30 × 13–17(–35) µm (n = 31); lower cell hyaline, cylindrical, 22–32 µm × 2–3 µm (n = 10); gelatinous appendages observed in the upper cell. Asexual morph not observed.

##### Culture characters.

Colonies on OA attaining a diameter of 36–40 mm after 14 days at 25 °C, cottony, lobulate, umbonate, margins regular to fringed, in the center black (202A) to silver gray (202C) and dark gray (202B); reverse silver gray (202C). Colonies on PCA attaining a diameter of 39–49 mm after 14 days at 25 °C, cottony, slightly lobulate, umbonate, margins fimbriate to fringed, in the center dark brown (200A) to gray-brown (199A), to gray (201D) margins; reverse brown (200A) to grayed green (197C). Colonies on PDA attaining a diameter of 24–37 mm after 14 days at 25 °C, cottony, lobulate, flat, margins fimbriate to fringed, in the center gray (201C to 201D) to gray green in the margins (198A); reverse grayed green (189A). Colonies on Cz attaining a diameter of 35–64 mm after 14 days at 25 °C, velvety, slightly lobulate, umbonate, margins fringed, in the center black (202A) to gray brown (199A) and lighter brown in the margins (199D); reverse center brown (200A) to gray brown (199A to 199D). Cardinal temperatures for growth: minimum 8 °C, optimal 25 °C, and maximum 28 °C.

##### Specimen examined.

Kenya, campus-side Egerton University, isolated from cow dung, isol. by K. Harms, col. by A. Wennrich and J.P. Wennrich (herbarium PRM 964937, ex-type culture CCF 6931 = YMF 175).

##### Notes.

*Schizothecium keniense* is introduced here to accommodate a fungus isolated from dung collected in Kenya. It is characterized by the distinctive agglutinated hairs in the upper part of the ascomata, a feature typical of *Schizothecium*. Although the fungus was isolated in pure culture, it did not sporulate; therefore, the description is based on the specimen observed on dung. As the material was already in a senescent state, asci and paraphyses could not be observed. Furthermore, the lower cell of the ascospores was frequently absent or collapsed in most observed spores.

The phylogenetically closest species to *S. keniense* is *S. conicum*. Morphologically, the two species are also highly similar, both possessing comparable ascospores and groups of agglutinated, swollen hairs on the upper part of the ascomata. *Schizothecium conicum* is one of the most common coprophilous species found on dung (Lundqvist 1972). The two species can be distinguished by the length of the agglutinated hairs and the lower ascospore cell, measuring up to 160 µm and 31.5 µm in *S. keniense*, respectively, vs. up to 120 µm and 14 µm in *S. conicum* (Lundqvist 1972; Mungai et al. 2012). *Schizothecium vesticola* is also phylogenetically related, but this can be easily distinguished in both mentioned species by the production of swollen hairs often non-agglutinated and not forming triangular scales at the neck base (Doveri 2008).

Other species included in the phylogenetic study and isolated from dung in Kenya were *S. curvuloides*, *S. dakotense*, and *S. glutinans*. However, these species were phylogenetically distant from the newly described taxon. *Schizothecium curvuloides* and *S. dakotense* differ in possessing much shorter agglutinated hairs, reaching up to 32 µm and 30 µm, respectively (Mungai et al. 2012). In addition, *S. curvuloides* produces ascospores with longer upper cells (31–45 µm) than the other species (Cai et al. 2005; Mungai et al. 2012), whereas *S. dakotense* has the shortest upper cells (18–24 µm) (Cai et al. 2005). *Schizothecium glutinans* is readily distinguished by producing two types of perithecial hairs: short, swollen agglutinated hairs and long, flexuous, non-agglutinated hairs (Mirza and Cain 1969). Moreover, it produces ascospores with longer upper cells (26.5–33 µm) than *S. keniense* (Cai et al. 2005; Mungai et al. 2012). *Schizothecium dubium* was also isolated from Kenyan dung samples, although no molecular data are available for comparison. This species is easily distinguished by producing swollen agglutinated cells rather than groups of agglutinated hairs (Mungai et al. 2012).

The genus *Schizothecium* comprises predominantly coprophilous species. Within the African records of the genus, *S. vesticola* has been reported from Algeria and Morocco (Lundqvist 1972). This species differs from *S. keniense* by producing short, swollen hairs that are often non-agglutinated and do not form triangular scales at the base of the neck, but instead occur as scattered, articulated elements (25–35 × 5–10 µm) or are reduced to single protruding cells (Doveri 2008). Moreover, the upper ascospore cell is shorter in *S. vesticola*, measuring 17–21.5 µm (Mirza and Cain 1969; Doveri 2008).

### Isolation and structural elucidation

**Figure 3.**
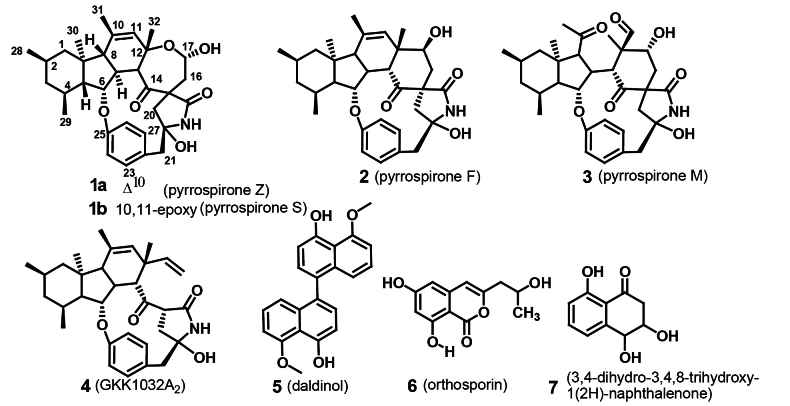
Structures of compounds **1**–**7**.

The strain *Schizothecium keniense*CCF 6931 was evaluated for its production of secondary metabolites on solid medium (BRFT). The EtOAc-soluble fraction of the crude acetone extract was subjected to preparative high-performance liquid chromatography (HPLC), affording pyrrospirone Z (**1a**) together with six known metabolites (Fig. 3). Structural elucidation was achieved through high-resolution mass spectrometry (HR-MS) and nuclear magnetic resonance (NMR) spectroscopy, further supported by comparison with previously reported data.

Compound **1a** was isolated as a white amorphous solid from 40% ACN/H_2_O. Its molecular formula C_32_H_41_NO_6_ was deduced from the HRESIMS, which exhibited a protonated molecular ion peak [M+H]^+^ at *m/z* 536.2987 (Calcd for C_32_H_42_NO_6_^+^: 536.3007, D -3.7 ppm). The UV, ^1^H, ^13^C, HSQC, COSY, and HMBC spectra of **1** (Supp. material 1: figs S1–S8) suggested that it is an analogue of pyrrospirone alkaloids (Song et al. 2018a, 2018b; Yao et al. 2022).

The analysis of the ^1^H NMR spectrum of **1a** revealed two methyl doublets at *δ*_H_ 0.91 (d, *J* = 6.5 Hz, CH_3_-28) and 1.11 (d, *J* = 6.3 Hz, CH_3_-29), along with three methyl singlets at *δ*_H_ 1.22 (s, CH_3_-30), 1.85 (s, CH_3_-31), and 1.36 (s, CH_3_-32). Additionally, four aromatic proton signals were observed at *δ*_H_ 7.12 (dd, *J* = 8.2, 2.2 Hz, H-23), 6.66 (dd, *J* = 7.9, 2.5 Hz, H-24), 6.92 (dd, *J* = 8.4, 2.5 Hz, H-26), and 7.14 (dd, *J* = 8.2, 2.1 Hz, H-27). These signals correspond to a para-substituted aromatic ring, thereby supporting the assignment of the compound to the decahydrofluorene class (Yao et al. 2022). The combination of HSQC and HMBC experiments enabled the identification of several key structural features, including a ketone carbon at *δ*_C_ 203.0 (C-14), an amide carbon at *δ*_C_ 175.9 (C-18), and six aromatic carbons at *δ*_C_ 131.0 (C-22), 132.3 (C-23), 123.3 (C-24), 158.5 (C-25), 119.2 (C-26), and 134.5 (C-27). Two olefinic carbons were also observed at *δ*_C_ 136.5 (C-10) and 132.6 (C-11), indicating the presence of a *γ*-lactam moiety and a decahydrofluorene ring system (Ariantari et al. 2020).

**Figure 4.**
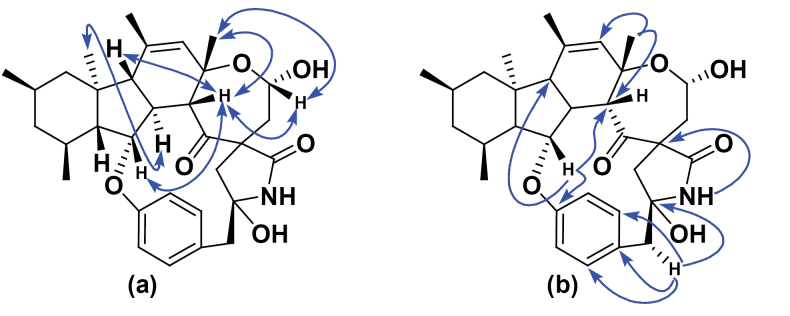
Key NOESY (**a**) and HMBC (**b**) correlations in **1a**.

The HMBC spectrum further supported the structural assignment. Thus, key correlations were observed from proton signal at *δ*_H_ 3.08 (H-20a) to carbon signals at *δ*_C_ 87.0 (C-19), 175.8 (C-18), and 46.6 (C-21), and from proton signals at *δ*_H_ 3.00 (H-21a) and 2.77 (H-21b) to carbon signals at *δ*_C_ 131.0 (C-22), 132.3 (C-23), and 134.5 (C-27), establishing the connection of the aromatic ring to the *γ*-lactam unit. Furthermore, a correlation from H-9 (*δ*_H_ 4.31) to C-25 (*δ*_C_ 158.5) confirmed the linkage of the aromatic ring to the five-membered ring of the decahydrofluorene core. The oxepan-4-one moiety was indicated by the signal at *δ*_H_ 5.71, corresponding to a proton attached to C-17 (*δ*_C_ 88.7). This assignment was supported by ^1^H–^1^H COSY correlations with two diastereotopic protons at *δ*_H_ 2.07 (H-16a) and 1.35 (H-16b), both of which exhibited long-range connectivity to the carbon at *δ*_C_ 175.9 (*γ*-lactam carbonyl group). The presence of a hemiacetal moiety was further corroborated by HMBC correlations involving the methyl group (CH_3_-32) and the diastereotopic protons (CH_2_-16). Notably, in contrast to the structure of pyrrospirone F, which shows four HMBC correlations (Song et al. 2018a), only three correlations were clearly observed on its spectrum. Another significant structural difference lies in the chemical shift of C-12, which appears downfield at *δ*_C_ 76.3, compared to approximately 46 ppm in pyrrospirone F and its analogues (Song et al. 2018a), but close to that of pyrrospirone S (**1b**) (Ban et al. 2025). Comparison of the data (Table 2) of the latter with those of **1a** further confirmed the presence of a seven-membered ring. The relative stereochemistry of **1a** was assigned based on the observed NOESY correlations. Correlations were observed between H-13 (*δ*_H_ 3.95) and four protons viz. H-7 (*δ*_H_ 1.85), H-9 (*δ*_H_ 4.31), CH_3_-32 (*δ*_H_ 1.36), and H-17 (*δ*_H_ 5.71), and also between H-17 (*δ*_H_ 5.71) and CH_3_-32 (*δ*_H_ 1.39) (Fig. 4), indicating their *β*-orientation (Song et al. 2018a). The *α*-orientation of H-8 (*δ*_H_ 2.73) and CH_3_-30 (*δ*_H_ 1.22) was supported by a unique NOE correlation observed between these two protons. The attributed relative stereochemistry of **1a** is consistent with that of previously reported pyrrospirones (Song et al. 2018a; Yao et al. 2022). Based on this comprehensive spectral evidence, the structure of compound **1a** was established.

The known compounds were identified as pyrrospirone F (**2**) (Song et al. 2018a), pyrrospirone M (**3**) (Yao et al. 2022), GKK1032A_2_ (**4**) (Qi et al. 2019), daldinol (**5**) (Hashimoto et al. 1994), orthosporin (**6**) (Ichihara et al. 1989), and 3,4-dihydro-3,4,8-trihydroxy-1(2H)-naphthalenone (**7**) (Ayer et al. 1989) based on comparisons of their spectrometric and spectroscopic data (Supp. material 1: figs S8–S49) with values reported in the literature.

Decahydrofluorene-class alkaloids are a group of secondary metabolites characterized by a tricyclic decahydrofluorene core, a 12- or 13-membered ether ring, a para-phenyl ether moiety, and either a *γ*-lactam or succinimide functional group (Yao et al. 2022). This class is divided into four main groups: GKK1032s, pyrrocidines, hirsutellones, and pyrrospirones (Yao et al. 2024). These compounds have so far only been reported from fungi, mainly from marine-derived and endophytic strains (Ariantari et al. 2020; Yao et al. 2022; Ban et al. 2025), making this study the first report from a coprophilous fungal isolate.

However, some metabolites isolated herein have previously been reported from phylogenetically related ascomycetes. In the case of pyrrospirone-type alkaloids, pyrrospirone F (**2**) and pyrrospirone M (**3**) have been reported previously from marine-derived *Penicillium* strains, such as *Penicillium* sp. ZZ380 and *Penicillium* sp. SCSIO 41512 (Song et al. 2018a; Yao et al. 2022). In addition, the production of daldinol (**5**) and 3,4-dihydro-3,4,8-trihydroxy-1(2H)-naphthalenone (**7**) has been reported by the sordariomycetes *Daldinia* spp. and *Leptographium wageneri* (Ayer et al. 1989; Stadler et al. 2014). These findings indicate that structurally related metabolites recur across Sordariomycetes and other ascomycetous lineages, suggesting conserved or convergent biosynthetic capabilities within these groups.

The detection of decahydrofluorene-class alkaloids in *Schizothecium keniense* expands the known taxonomic and ecological distribution of these compounds beyond marine and endophytic habitats. While further in situ metabolomic studies are required to confirm the production of these metabolites within the natural dung substrate, this finding highlights the genetic potential of dung-inhabiting fungi and supports the hypothesis that this group represents an underexplored reservoir of chemically prolific taxa.

### Antimicrobial and cytotoxic activities

Compounds **1**–**7** were evaluated for antimicrobial activity against a panel of fungi as well as gram-negative and gram-positive bacteria (Table 3). Among the isolates, pyrrospirone F (**2**) and GKK1032A2 (**4**) exhibited activity against some of the tested microorganisms. Both metabolites showed mild antifungal activity against the filamentous fungus *Mucor hiemalis* at a concentration of 33.3 μg/mL. Compounds **2** and **4** also displayed antibacterial activity against Gram-positive bacteria. Notably, compound **4** showed the strongest effect against *Bacillus subtilis* with an MIC value of 4.2 μg/mL, followed by compound **2** with an MIC of 8.3 μg/mL; both values were more potent than that of the positive control oxytetracycline (MIC = 16.6 μg/mL). Both compounds also showed a notably inhibitory effect against *Staphylococcus aureus* with MIC values of 8.3 μg/mL. In contrast, compounds **5**–**7** did not exhibit any antimicrobial activity.

**Table 3.** Cytotoxicity and antimicrobial activity of compounds **1**–**7**.

	**IC_50_ (µM)**				**Positive control**
**test cell line**	**1a**	**2**	**3**	**4**	**epothilone B (µg/mL)**
Mouse fibroblast (L929)	n.d.	22	-	7.6	0.00098
Human endocervical adenocarcinoma (KB3.1)	21	6.7	11	6.4	0.000028
Human prostate carcinoma (PC-3)	18	9.5	17	2.0	0.00075
Human ovarian cancer (SKOV-3)	n.d.	17	26	8.1	0.0016
Human breast adenocarcinoma (MCF-7)	n.d.	7.0	7.8	7.0	0.0026
Human epidermoid carcinoma (A431)	n.d.	8.1	8.5	3.9	0.000041
Human lung carcinoma (A549)	n.d.	7.4	14	7.3	0.000078
**test microorganism**	**MIC (µg/mL)**				**positive control (µg/mL)**
*Schizosaccharomyces pombe* (DSM 70572)	n.d.	33.3	-	-	4.2 ^N^
*Wickerhamomyces anomalus* (DSM 6766)	n.d.	-	-	-	4.2 ^N^
*Mucor hiemalis* (DSM 2656)	-	33.3	-	33.3	2.1 ^N^
*Candida albicans* (DSM 1665)	-	66.6	-	-	2.1 ^N^
*Rhodosporidium toruloides* (DSM 10134)	n.d.	66.6	-	-	1.0 ^N^
*Acinetobacter baumannii* (DSM 30008)	-	-	-	-	0.53 ^C^
*Escherichia coli* (DSM 116)	-	-	-	-	0.42 ^G^
*Bacillus subtilis* (DSM 10)	n.d.	8.3	-	4.2	16.6 ^O^
*Mycolicibacterium smegmatis* (ATCC 700084)	n.d.	66.6	-	-	0.1 ^K^
*Staphylococcus aureus* (DSM 346)	66.6	8.3	-	8.3	0.42 ^G^
*Pseudomonas aeruginosa* (PA 14)	n.d.	-	-	-	0.21 ^G^
*Chromobacterium violaceum* (DSM 30191)	n.d.	-	-	-	0.83 ^G^

IC_50_: half-maximal inhibitory concentration, µg/mL. MIC: minimum inhibitory concentration in µg/mL. “-“: No activity under test conditions (MIC > 66.6 µg/mL, IC_50_ > 37). n.d.: Not determined. C: Ciprofloxacin; G: Gentamicin; K: Kanamycin, N: Nystatin; O: Oxytetracycline. Compounds **5**–**7** were inactive in all assays.

Pyrrospirone F (**2**) has previously been reported to exhibit antimicrobial activity against *S. aureus* and methicillin-resistant *S. aureus* (MRSA), with MIC values of 25.8 μg/mL and 2.0 μg/mL, respectively (Song et al. 2018a; Qi et al. 2019). Furthermore, Qi et al. (2019) demonstrated that GKK1032A2 (**4**), isolated from the endophytic fungus *Penicillium* sp. CPCC 400817, displayed potent activity against *S. aureus* and MRSA (3.2 μg/mL). In contrast to our findings, Song et al. (2018a) reported that compound **2** also exhibited activity against *Escherichia coli* with an MIC of 3.0 μg/mL, whereas in the present study it was considered inactive, showing an MIC > 66.6 μg/mL. Previous studies have indicated that major structural alterations to the decahydrofluorene core, such as disruption of the carbon bond between C-10 and C-1, significantly decrease the antibacterial activity of this class of alkaloids (Yao et al. 2022). This structural feature may explain why pyrrospirone Z (**1a**) did not exhibit any significant antimicrobial activity.

Regarding cytotoxicity, compounds **2**–**4** exhibited moderate to strong activity, with GKK1032A2 (**4**) being the most potent, showing IC_50_ values ranging from 2.0 to 8.1 µM across all tested cell lines. Pyrrospirone F (**2**) displayed mild cytotoxicity (IC_50_ = 6.7–22 µM), followed by pyrrospirone M (**3**), which showed IC_50_ values between 7.8 and 26 µM. In contrast, pyrrospirone Z (**1a**) exhibited only weak cytotoxicity, with IC_50_ values of 18 µM in PC-3 cells and 21 µM in KB3.1 cells. Compounds **5**–**7** did not display any detectable cytotoxic activity.

## Conclusion

This study represents the first chemical investigation of a strain belonging to the genus *Schizothecium (Schizotheciaceae)*. It reveals its potential to produce structurally diverse secondary metabolites, including the newly described decahydrofluorene-class alkaloid, pyrrospirone Z, and six additional known compounds. Among these, the alkaloid GKK1032A2 exhibited notable antibacterial activity against Gram-positive bacteria and strong cytotoxicity across all tested cell lines.

The discovery of these metabolites from a fungus isolated from dung underscores the potential of this poorly explored substrate to harbor undocumented fungal lineages and unique specialized metabolites. This also highlights the importance of studying underexplored biodiversity hotspots as Kenya.

By integrating fungal phylogenetics with secondary metabolite profiling, this work expands current knowledge of compounds produced within the order *Sordariales* and emphasizes the value of unexplored ecological niches, such as dung, in the search for novel natural products.

## Supplementary Material

XML Treatment for Schizothecium keniense

## Additional information

### Artificial Intelligence (AI) use

The authors accept full responsibility for the content of the manuscript, including the disclosure of any use of AI.

No AI tools were used in the preparation of this manuscript.

### Author ORCIDs

M. Agudelo-Restrepo https://orcid.org/0009-0003-5842-202X

K. Harms https://orcid.org/0000-0002-5867-0846

J. Tchamgoue https://orcid.org/0000-0002-0506-9678

M. Stadler https://orcid.org/0000-0002-7284-8671

J. Matasyoh https://orcid.org/0000-0003-1209-9805

S.F. Kouam https://orcid.org/0000-0003-0191-0527

Y. Marin-Felix https://orcid.org/0000-0001-8045-4798

### Data availability

All of the data that support the findings of this study are available in the main text or Supplementary Information.

## Supplementary materials

Supplementary material 1 Data related to structure elucidation, and phylogenetic analysis Data type docx Explanation note **fig. S1**. UV spectrum of pyrrospirone Z (1); **fig. S2**. (+)-HR-ESI mass spectrum of pyrrospirone Z (1); **fig. S3**. 1H NMR spectrum of pyrrospirone Z (1) (Acetone-d_6_, 700 MHz); **fig. S4**. HSQC-DEPT spectrum of pyrrospirone Z (1); **fig. S5**. HMBC spectrum of pyrrospirone Z (1); **fig. S6**. COSY spectrum of pyrrospirone Z (1); **fig. S7**. NOESY spectrum of pyrrospirone Z (1); **fig. S8**. UV spectrum of pyrrospirone F (2); **fig. S9**. (+)-HR-ESI mass spectrum of pyrrospirone F (2); **fig. S10**. 1H NMR spectrum of pyrrospirone F (2) (Acetone-d_6_, 500 MHz); **fig. S11**. 13C NMR spectrum of pyrrospirone F (2) (Acetone-d_6_, 125 MHz); **fig. S12**. HSQC-DEPT spectrum of pyrrospirone F (2); **fig. S13**. HMBC spectrum of pyrrospirone F (2); **fig. S14**. COSY spectrum of pyrrospirone F (2); **fig. S15**. NOESY spectrum of pyrrospirone F (2); **fig. S16**. UV spectrum of pyrrospirone M (3); **fig. S17**. (+)-HR-ESI mass spectrum of pyrrospirone M (3); **fig. S18**. 1H NMR spectrum of pyrrospirone M (3) (Acetone-d_6_, 500 MHz); **fig. S19**. 13C NMR spectrum of pyrrospirone M (3) (Acetone-d_6_, 125 MHz); **fig. S20**. HSQC-DEPT spectrum of pyrrospirone M (3); **fig. S21**. HMBC spectrum of pyrrospirone M (3); **fig. S22**. COSY spectrum of pyrrospirone M (3); **fig. S23**. NOESY spectrum of pyrrospirone M (3); **fig. S24**. UV spectrum of GKK1032A2 (4); **fig. S25**. (+)-HR-ESI mass spectrum of GKK1032A2 (4); **fig. S26**. 1H NMR spectrum of GKK1032A2 (4) (Acetone-d_6_, 600 MHz); **fig. S27**. 13C NMR spectrum of GKK1032A2 (4) (Acetone-d_6_, 150 MHz); **fig. S28**. HSQC-DEPT spectrum of GKK1032A2 (4); **fig. S29**. HMBC spectrum of GKK1032A2 (4); **fig. S30**. COSY spectrum of GKK1032A2 (4); **fig. S31**. UV spectrum of daldinol (5); **fig. S32**. (+)-HR-ESI mass spectrum of daldinol (5); **fig. S33**. 1H NMR spectrum of daldinol (5) (Acetone-d_6_, 500 MHz); **fig. S34**. HSQC-DEPT spectrum of daldinol (5); **fig. S35**. HMBC spectrum of daldinol (5); **fig. S36**. COSY spectrum of daldinol (5); **fig. S37**. UV spectrum of orthosporin (6); **fig. S38**. (+)-HR-ESI mass spectrum of orthosporin (6); **fig. S39**. 1H NMR spectrum of orthosporin (6) (Acetone-d_6_, 600 MHz); **fig. S40**. HSQC-DEPT spectrum of orthosporin (6); **fig. S41**. HMBC spectrum of orthosporin (6); **fig. S42**. COSY spectrum of orthosporin (6); **fig. S43**. UV spectrum of 3,4-dihydro-3,4,8-trihydroxy-1(2H)-naphthalenone (7); **fig. S44**. (+)-HR-ESI mass spectrum of 3,4-dihydro-3,4,8-trihydroxy-1(2H)-naphthalenone (7); **fig. S45**. 1H NMR spectrum of 3,4-dihydro-3,4,8-trihydroxy-1(2H)-naphthalenone (7) (Acetone-d_6_, 600 MHz); **fig. S46**. 13C NMR spectrum of 3,4-dihydro-3,4,8-trihydroxy-1(2H)-naphthalenone (7) (Acetone-d_6_, 150 MHz); **fig. S47**. HSQC-DEPT spectrum of 3,4-dihydro-3,4,8-trihydroxy-1(2H)-naphthalenone (7) ; **fig. S48**. HMBC spectrum of 3,4-dihydro-3,4,8-trihydroxy-1(2H)-naphthalenone (7); **fig. S49**. COSY spectrum of 3,4-dihydro-3,4,8-trihydroxy-1(2H)-naphthalenone (7); **table SS1**. Alignment used in the phylogenetic study. https://binary.pensoft.net/file/1709437This dataset is made available under the Open Database License (http://opendatacommons.org/licenses/odbl/1.0/). The Open Database License (ODbL) is a license agreement intended to allow users to freely share, modify, and use this Dataset while maintaining this same freedom for others, provided that the original source and author(s) are credited. Manuela Agudelo-Restrepo, Patrick Dibouloul, Karen Harms, Joseph Tchamgoue, Marc Stadler, Josphat Matasyoh, Simeon Fogue Kouam, Yasmina Marin-Felix
