# Supplementary material for: Pyrrospirone Z and related decahydrofluorene alkaloids produced by the new coprophilous species Schizothecium keniense (Schizotheciaceae, Sordariales)
Source: MycoKeys. 2026 Jul 7;136:139–59. doi: 10.3897/mycokeys.136.191424 (PMC13370148; doi:10.3897/mycokeys.136.191424)
Supplement: Supplementary material 1 — Data related to structure elucidation, and phylogenetic analysis [file mycokeys-136-139-s001.docx]

**Supplementary information to:**

**Pyrrospirone Z and related decahydrofluorene alkaloids produced by the new coprophilous species *Schizothecium keniense* (*Schizotheciaceae, Sordariales*)**

Manuela Agudelo-Restrepo^1,2^, Patrick Dibouloul^3,4^, Karen Harms^1,2^, Joseph Tchamgoue^3,4^, Marc Stadler^1^, Josphat Matasyoh^5^, Simeon Fogue Kouam^3*^, Yasmina Marin-Felix^1,2*^

^1^ Department of Microbial Drugs, Helmholtz Centre for Infection Research GmbH (HZI), and German Centre for Infection Research Association (DZIF), Inhoffenstraße 7, 38124 Braunschweig, Germany

^2^ Institute of Microbiology, Technische Universität Braunschweig, Spielmannstraße 7, 38106 Braunschweig, Germany

^3^ Department of Chemistry, Higher Teacher Training College, University of Yaounde 1, P.O. Box 47, Yaounde, Cameroon

^4^ Department of Organic Chemistry, Faculty of Science, University of Yaounde 1, P.O. Box 812, Yaounde, Cameroon

^5^ Department of Chemistry, Egerton University, P.O. Box 536, Egerton 20115, Kenya

Correspondence: Simeon Fogue Kouam (kfogue@yahoo.com); Yasmina Marin-Felix (yasmina.marinfelix@helmholtz-hzi.de)

**Table of content**

[Figure S1. UV spectrum of pyrrospirone Z (1) S4](#_Toc222937480)

[Figure S2. (+)-HR-ESI mass spectrum of pyrrospirone Z (1) S4](#_Toc222937481)

[Figure S3. ^1^H NMR spectrum of pyrrospirone Z (1) (Acetone-*d₆*, 700 MHz) S4](#_Toc222937482)

[Figure S4. HSQC-DEPT spectrum of pyrrospirone Z (1) S5](#_Toc222937483)

[Figure S5. HMBC spectrum of pyrrospirone Z (1) S5](#_Toc222937484)

[Figure S6. COSY spectrum of pyrrospirone Z (1) S6](#_Toc222937485)

[Figure S7. NOESY spectrum of pyrrospirone Z (1) S6](#_Toc222937486)

[Figure S8. UV spectrum of pyrrospirone F (2) S7](#_Toc222937487)

[Figure S9. (+)-HR-ESI mass spectrum of pyrrospirone F (2) S7](#_Toc222937488)

[Figure S10. ^1^H NMR spectrum of pyrrospirone F (2) (Acetone-*d₆*, 500 MHz) S7](#_Toc222937489)

[Figure S11. ^13^C NMR spectrum of pyrrospirone F (2) (Acetone-*d₆*, 125 MHz) S8](#_Toc222937490)

[Figure S12. HSQC-DEPT spectrum of pyrrospirone F (2) S8](#_Toc222937491)

[Figure S13. HMBC spectrum of pyrrospirone F (2) S9](#_Toc222937492)

[Figure S14. COSY spectrum of pyrrospirone F (2) S9](#_Toc222937493)

[Figure S15. NOESY spectrum of pyrrospirone F (2) S10](#_Toc222937494)

[Figure S16. UV spectrum of pyrrospirone M (3) S10](#_Toc222937495)

[Figure S17. (+)-HR-ESI mass spectrum of pyrrospirone M (3) S10](#_Toc222937496)

[Figure S18. ^1^H NMR spectrum of pyrrospirone M (3) (Acetone-*d₆*, 500 MHz) S11](#_Toc222937497)

[Figure S19. ^13^C NMR spectrum of pyrrospirone M (3) (Acetone-*d₆*, 125 MHz) S11](#_Toc222937498)

[Figure S20. HSQC-DEPT spectrum of pyrrospirone M (3) S12](#_Toc222937499)

[Figure S21. HMBC spectrum of pyrrospirone M (3) S12](#_Toc222937500)

[Figure S22. COSY spectrum of pyrrospirone M (3) S13](#_Toc222937501)

[Figure S23. NOESY spectrum of pyrrospirone M (3) S13](#_Toc222937502)

[Figure S24. UV spectrum of GKK1032A2 (4) S14](#_Toc222937503)

[Figure S25. (+)-HR-ESI mass spectrum of GKK1032A2 (4) S14](#_Toc222937504)

[Figure S26. ^1^H NMR spectrum of GKK1032A2 (4) (Acetone-*d₆*, 600 MHz) S14](#_Toc222937505)

[Figure S27. ^13^C NMR spectrum of GKK1032A2 (4) (Acetone-d₆, 150 MHz) S15](#_Toc222937506)

[Figure S28. HSQC-DEPT spectrum of GKK1032A2 (4) S15](#_Toc222937507)

[Figure S29. HMBC spectrum of GKK1032A2 (4) S16](#_Toc222937508)

[Figure S30. COSY spectrum of GKK1032A2 (4) S16](#_Toc222937509)

[Figure S31. UV spectrum of daldinol (5) S17](#_Toc222937510)

[Figure S32. (+)-HR-ESI mass spectrum of daldinol (5) S17](#_Toc222937511)

[Figure S33. ^1^H NMR spectrum of daldinol (5) (Acetone-*d₆*, 500 MHz) S17](#_Toc222937512)

[Figure S34. HSQC-DEPT spectrum of daldinol (5) S18](#_Toc222937513)

[Figure S35. HMBC spectrum of daldinol (5) S18](#_Toc222937514)

[Figure S36. COSY spectrum of daldinol (5) S19](#_Toc222937515)

[Figure S37. UV spectrum of orthosporin (6) S19](#_Toc222937516)

[Figure S38. (+)-HR-ESI mass spectrum of orthosporin (6) S19](#_Toc222937517)

[Figure S39. ^1^H NMR spectrum of orthosporin (6) (Acetone-*d₆*, 600 MHz) S20](#_Toc222937518)

[Figure S40. HSQC-DEPT spectrum of orthosporin (6) S20](#_Toc222937519)

[Figure S41. HMBC spectrum of orthosporin (6) S21](#_Toc222937520)

[Figure S42. COSY spectrum of orthosporin (6) S21](#_Toc222937521)

[Figure S43. UV spectrum of 3,4-dihydro-3,4,8-trihydroxy-1(2H)-naphthalenone (7) S22](#_Toc222937522)

[Figure S44. (+)-HR-ESI mass spectrum of 3,4-dihydro-3,4,8-trihydroxy-1(2H)-naphthalenone (7) S22](#_Toc222937523)

[Figure S45. ^1^H NMR spectrum of 3,4-dihydro-3,4,8-trihydroxy-1(2H)-naphthalenone (7) (Acetone-*d₆*, 600 MHz) S22](#_Toc222937524)

[Figure S46. ^13^C NMR spectrum of 3,4-dihydro-3,4,8-trihydroxy-1(2H)-naphthalenone (7) (Acetone-*d₆*, 150 MHz) S23](#_Toc222937525)

[Figure S47. HSQC-DEPT spectrum of 3,4-dihydro-3,4,8-trihydroxy-1(2H)-naphthalenone (7) S23](#_Toc222937526)

[Figure S48. HMBC spectrum of 3,4-dihydro-3,4,8-trihydroxy-1(2H)-naphthalenone (7) S24](#_Toc222937527)

[Figure S49. COSY spectrum of 3,4-dihydro-3,4,8-trihydroxy-1(2H)-naphthalenone (7) S24](#_Toc222937528)

Table S1[. Alignment used in the phylogenetic study. S25](#_Toc222937528)

Figure S1. UV spectrum of pyrrospirone Z (1)

Figure S2. (+)-HR-ESI mass spectrum of pyrrospirone Z (1)


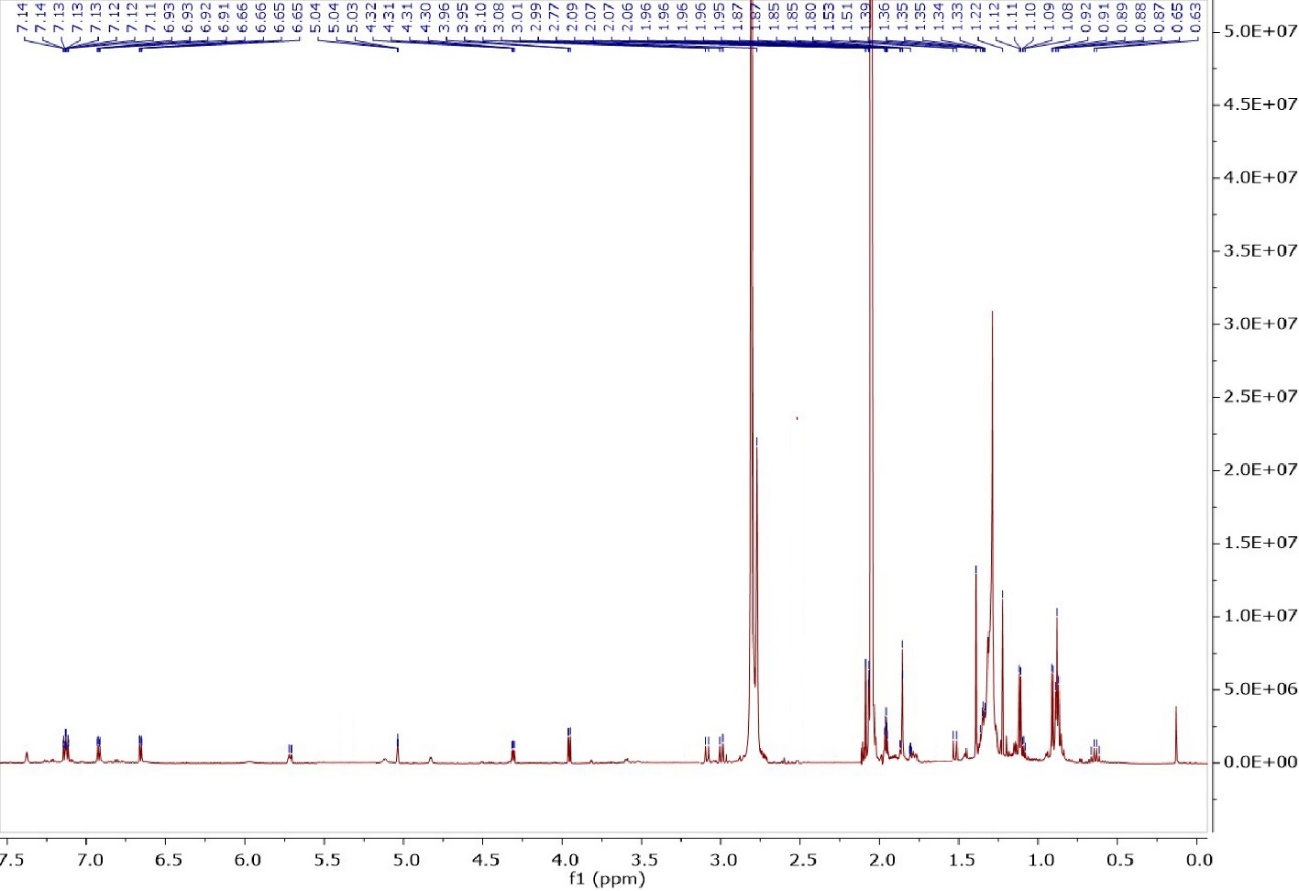


Figure S3. ^1^H NMR spectrum of pyrrospirone Z (1) (Acetone-*d₆*, 700 MHz)

Figure S4. HSQC-DEPT spectrum of pyrrospirone Z (1)

Figure S5. HMBC spectrum of pyrrospirone Z (1)

Figure S6. COSY spectrum of pyrrospirone Z (1)

Figure S7. NOESY spectrum of pyrrospirone Z (1)


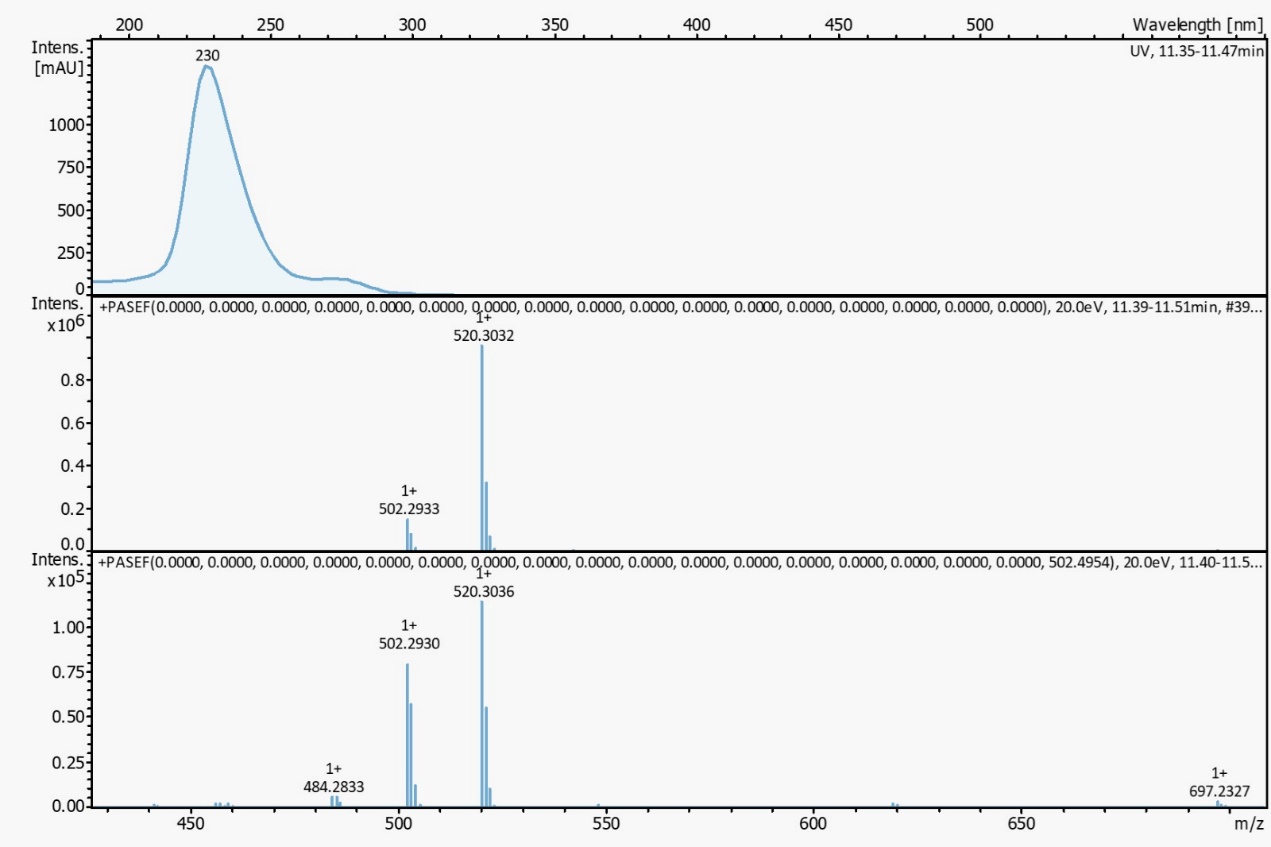


Figure S8. UV spectrum of pyrrospirone F (2)


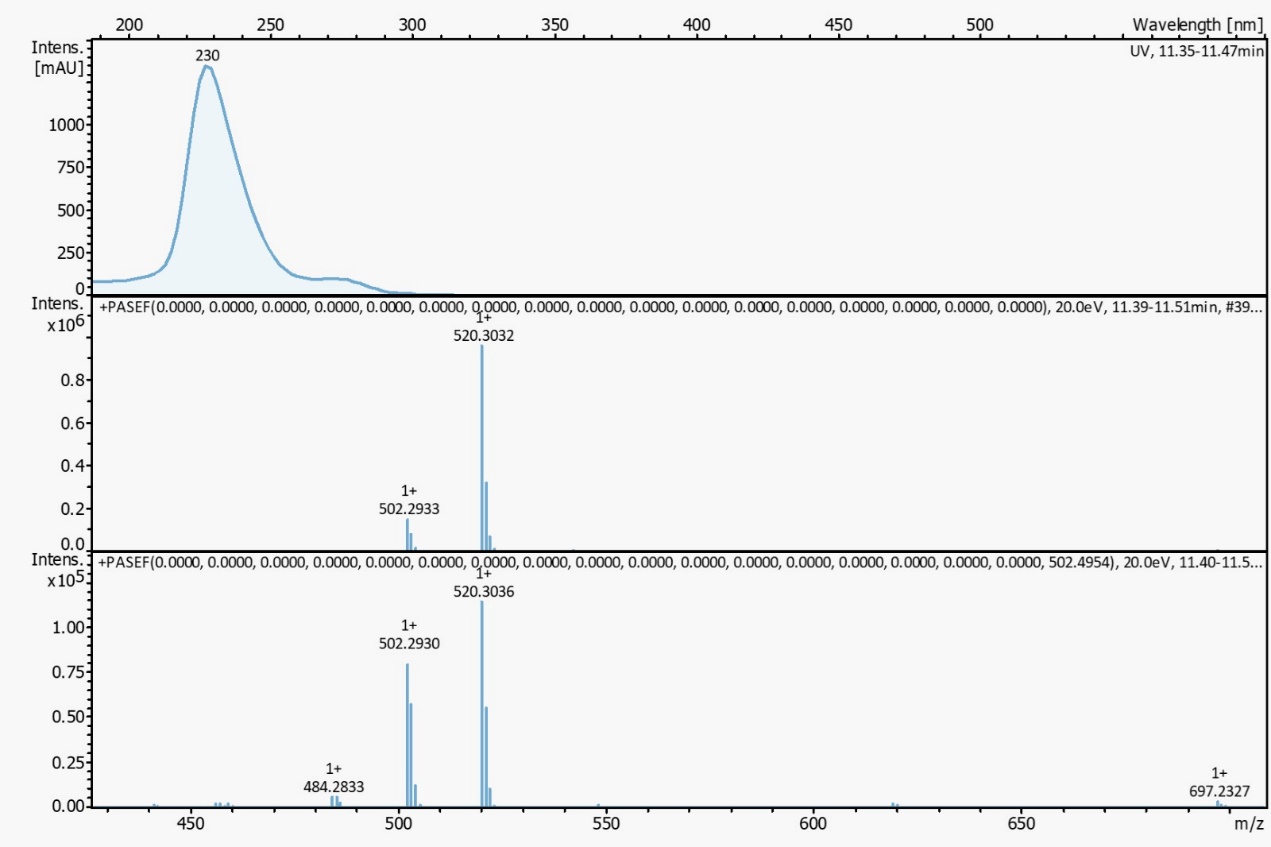


Figure S9. (+)-HR-ESI mass spectrum of pyrrospirone F (2)

Figure S10. ^1^H NMR spectrum of pyrrospirone F (2) (Acetone-*d₆*, 500 MHz)

Figure S11. ^13^C NMR spectrum of pyrrospirone F (2) (Acetone-*d₆*, 125 MHz)

Figure S12. HSQC-DEPT spectrum of pyrrospirone F (2)

Figure S13. HMBC spectrum of pyrrospirone F (2)

Figure S14. COSY spectrum of pyrrospirone F (2)

Figure S15. NOESY spectrum of pyrrospirone F (2)


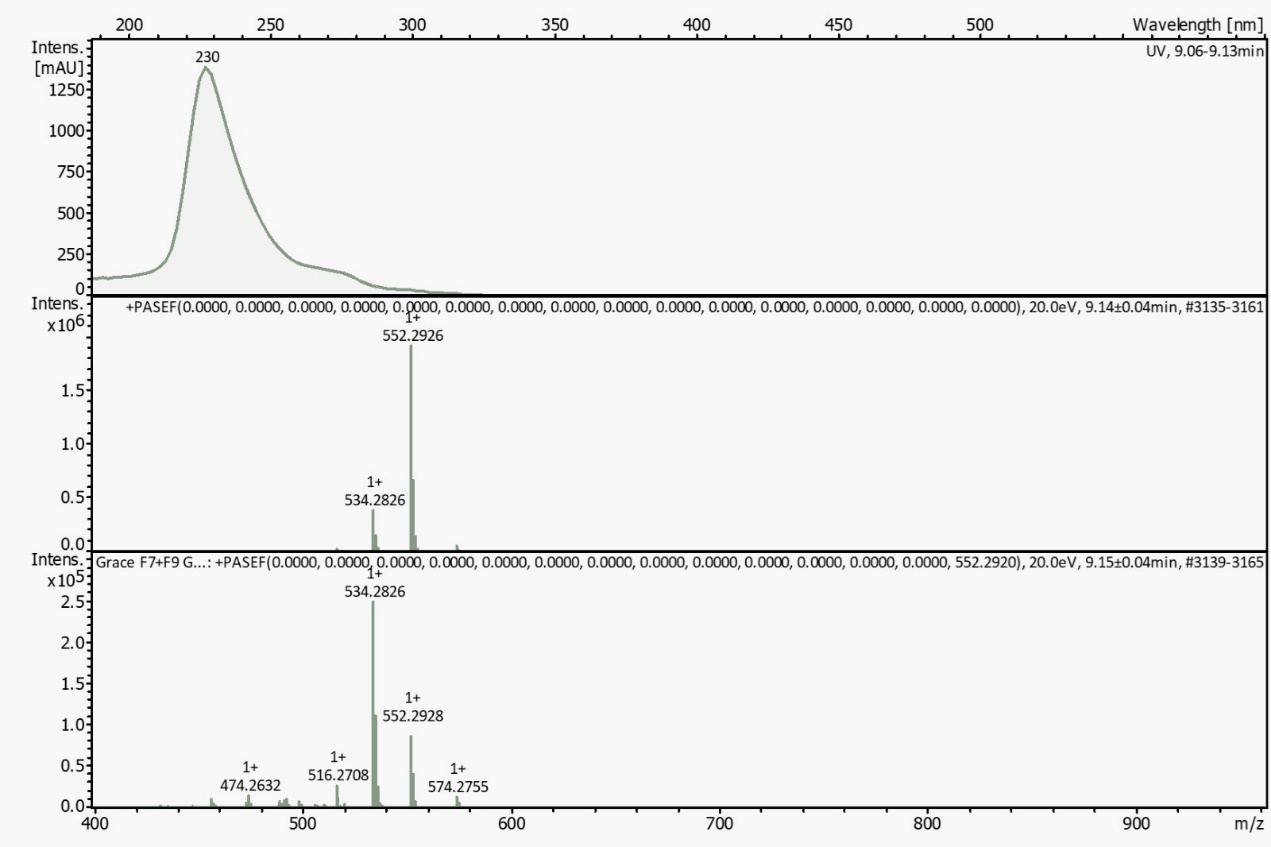


Figure S16. UV spectrum of pyrrospirone M (3)


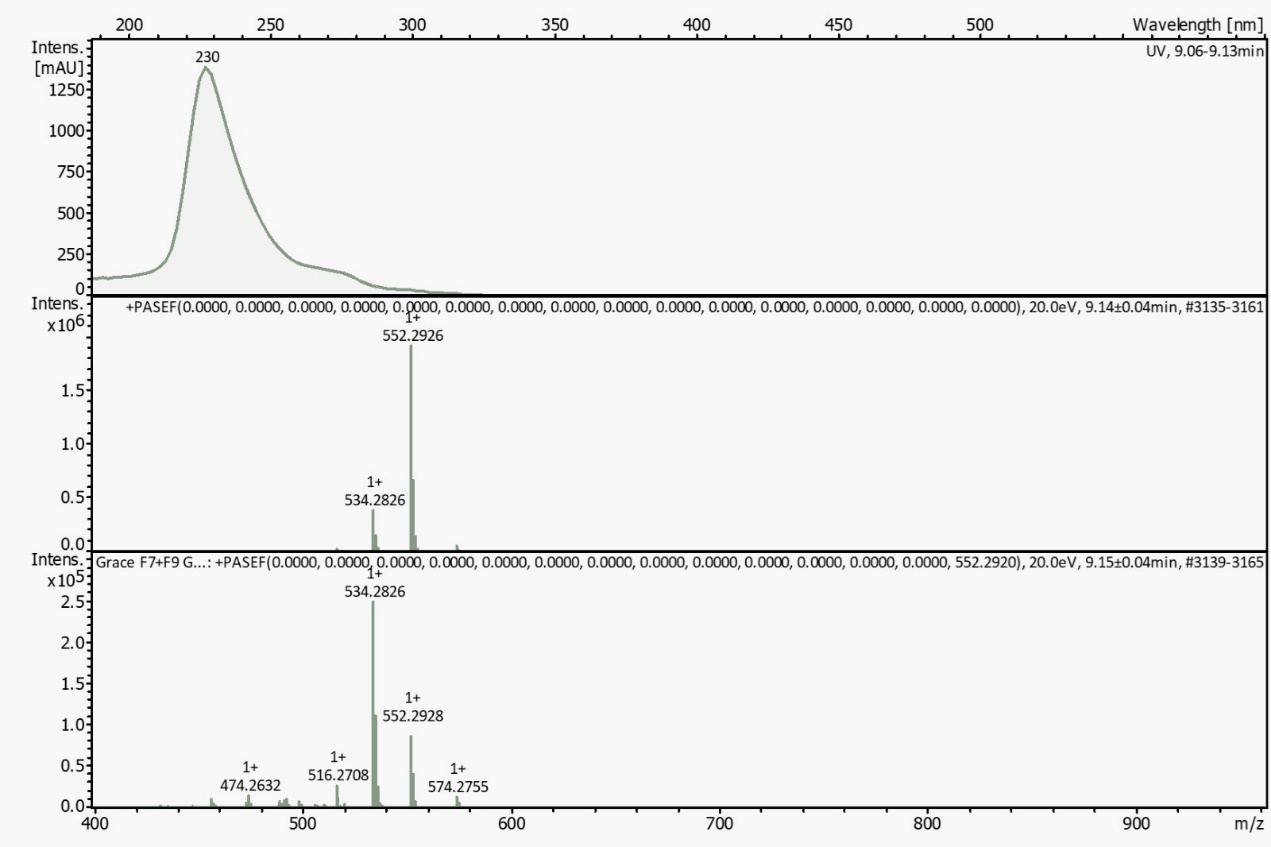


Figure S17. (+)-HR-ESI mass spectrum of pyrrospirone M (3)

Figure S18. ^1^H NMR spectrum of pyrrospirone M (3) (Acetone-*d₆*, 500 MHz)

Figure S19. ^13^C NMR spectrum of pyrrospirone M (3) (Acetone-*d₆*, 125 MHz)

Figure S20. HSQC-DEPT spectrum of pyrrospirone M (3)

Figure S21. HMBC spectrum of pyrrospirone M (3)

Figure S22. COSY spectrum of pyrrospirone M (3)

Figure S23. NOESY spectrum of pyrrospirone M (3)


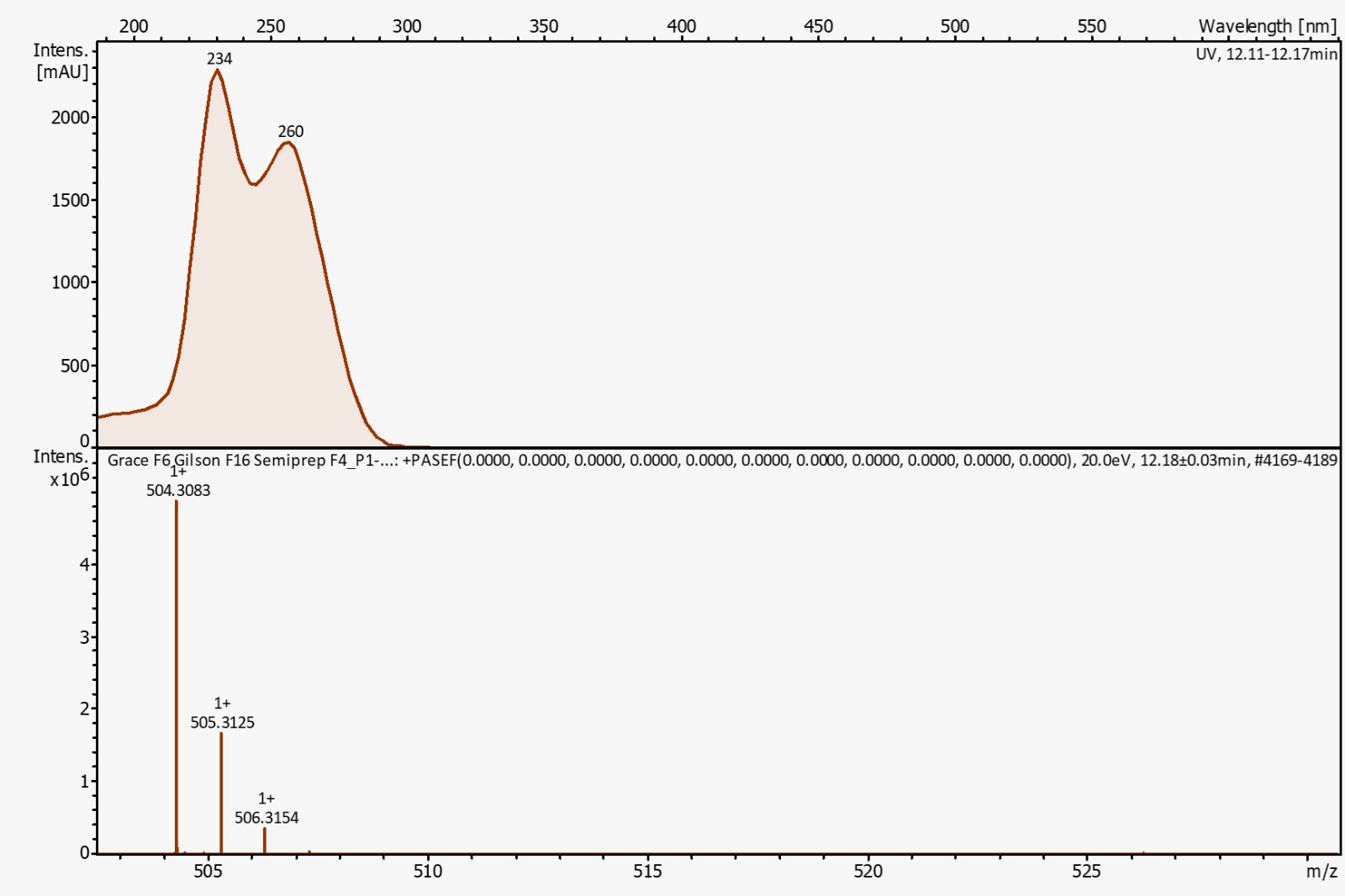


Figure S24. UV spectrum of GKK1032A2 (4)


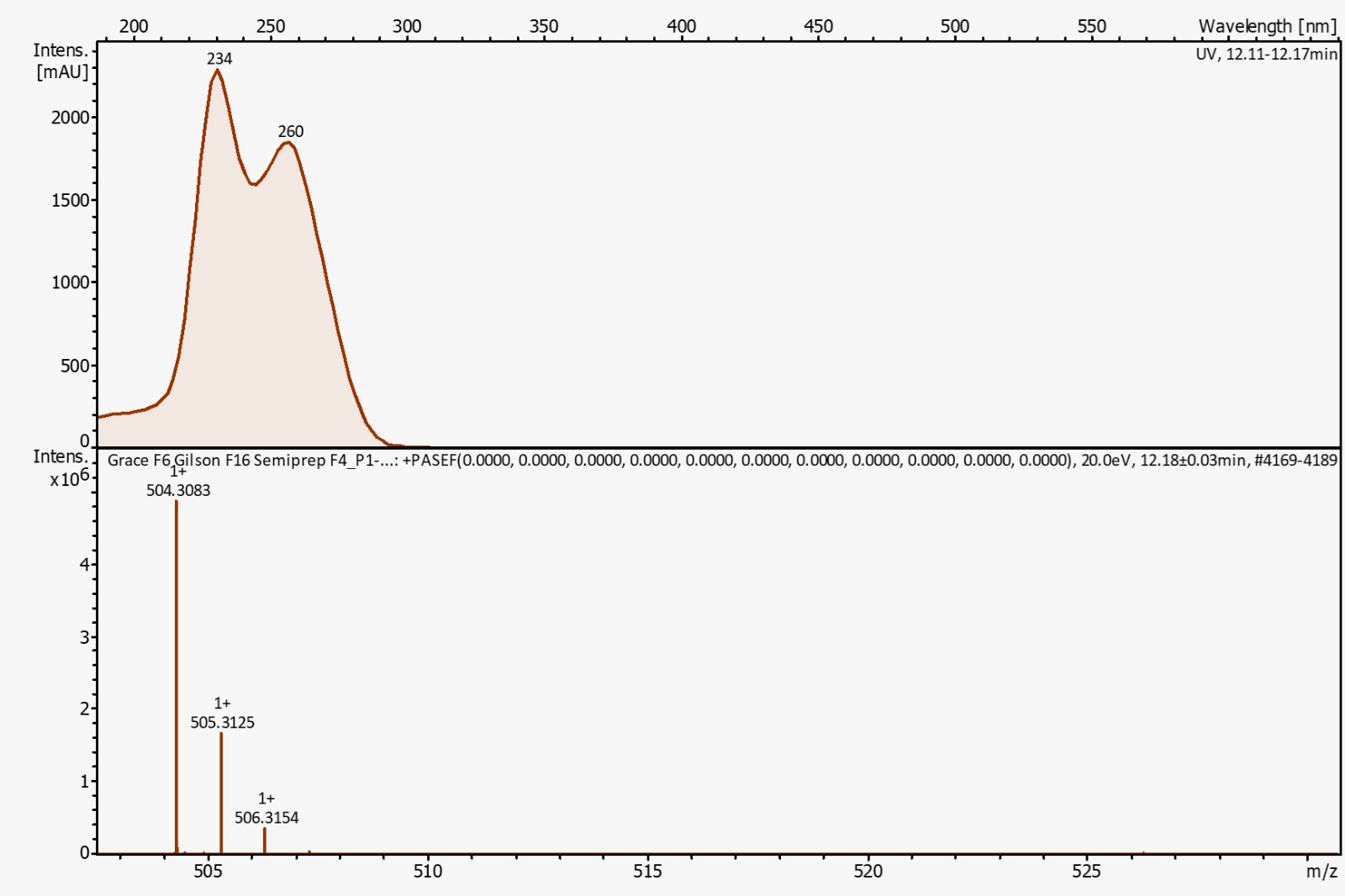


Figure S25. (+)-HR-ESI mass spectrum of GKK1032A2 (4)

Figure S26. ^1^H NMR spectrum of GKK1032A2 (4) (Acetone-*d₆*, 600 MHz)

Figure S27. ^13^C NMR spectrum of GKK1032A2 (4) (Acetone-d₆, 150 MHz)

Figure S28. HSQC-DEPT spectrum of GKK1032A2 (4)

Figure S29. HMBC spectrum of GKK1032A2 (4)

Figure S30. COSY spectrum of GKK1032A2 (4)


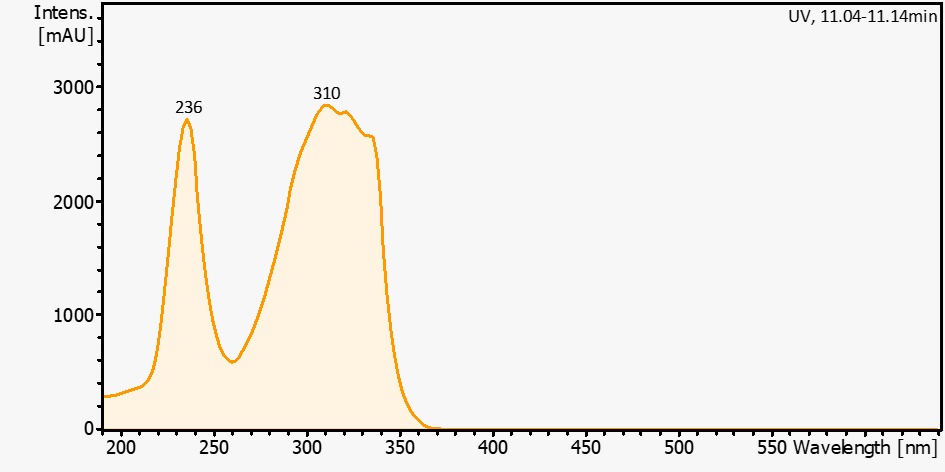


Figure S31. UV spectrum of daldinol (5)


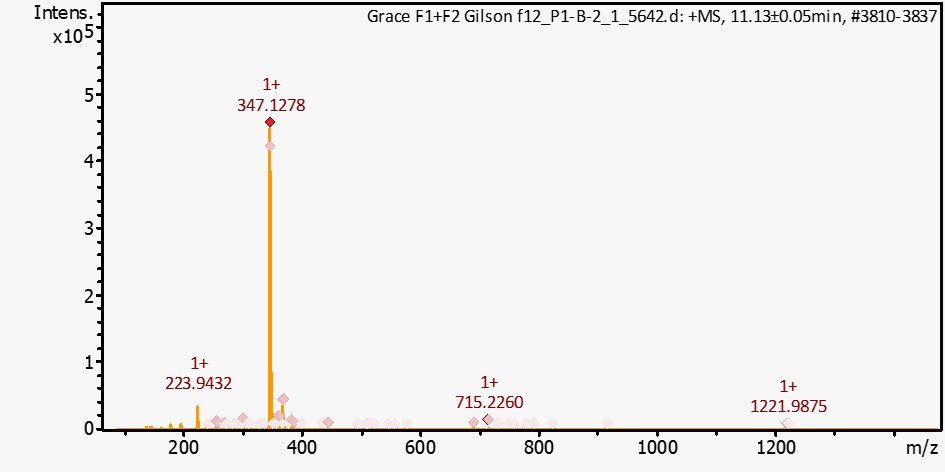


Figure S32. (+)-HR-ESI mass spectrum of daldinol (5)

Figure S33. ^1^H NMR spectrum of daldinol (5) (Acetone-*d₆*, 500 MHz)

Figure S34. HSQC-DEPT spectrum of daldinol (5)

Figure S35. HMBC spectrum of daldinol (5)

Figure S36. COSY spectrum of daldinol (5)


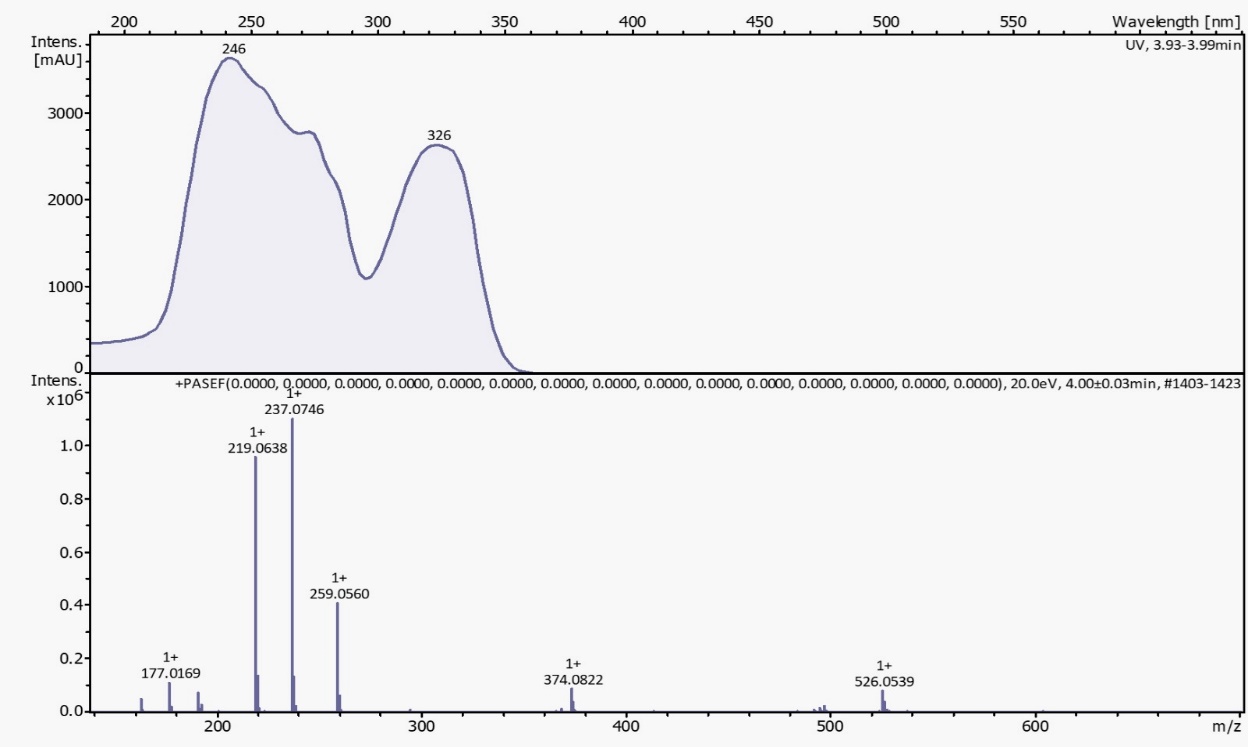


Figure S37. UV spectrum of orthosporin (6)


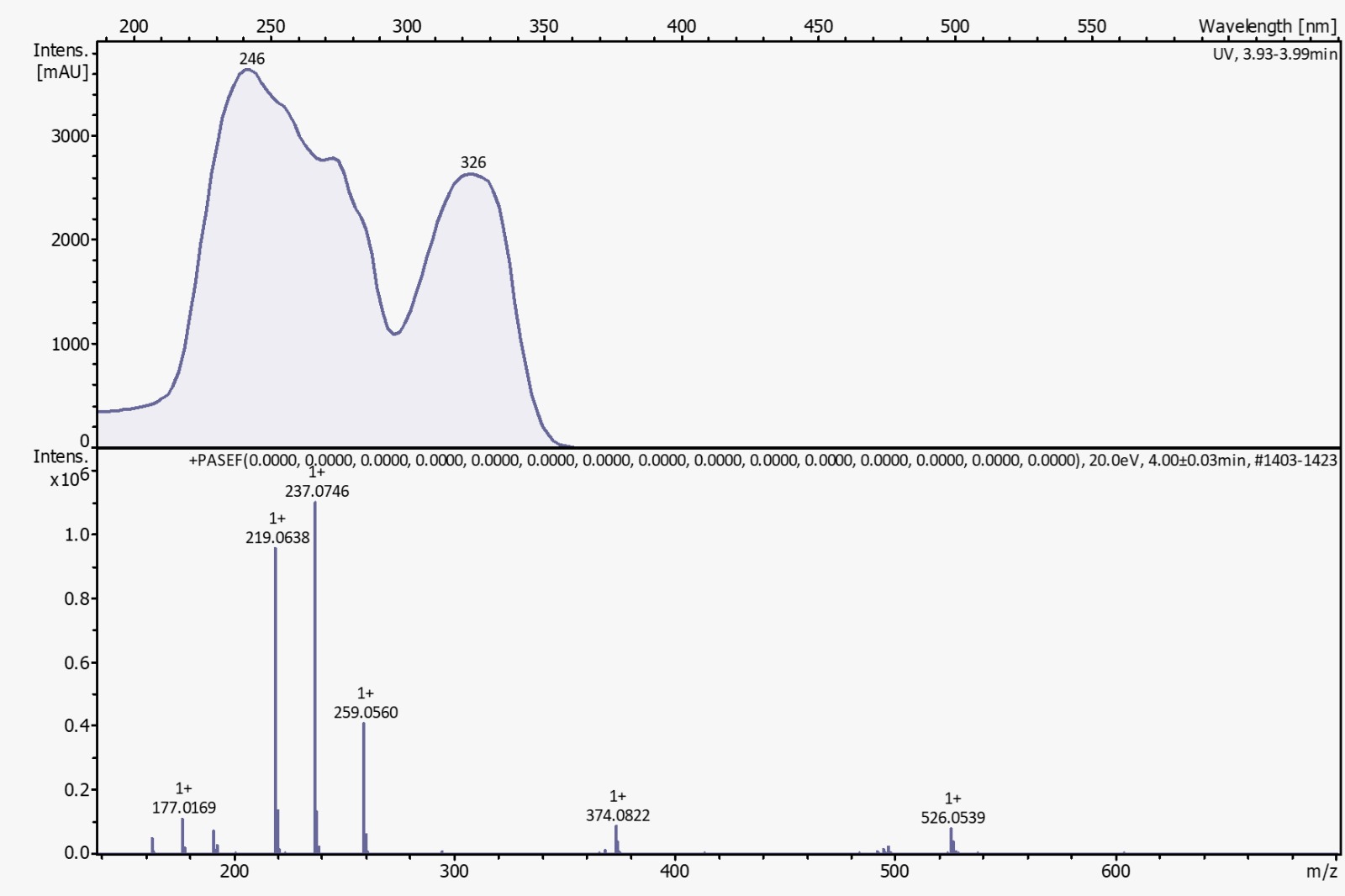


Figure S38. (+)-HR-ESI mass spectrum of orthosporin (6)

Figure S39. ^1^H NMR spectrum of orthosporin (6) (Acetone-*d₆*, 600 MHz)

Figure S40. HSQC-DEPT spectrum of orthosporin (6)

Figure S41. HMBC spectrum of orthosporin (6)

Figure S42. COSY spectrum of orthosporin (6)


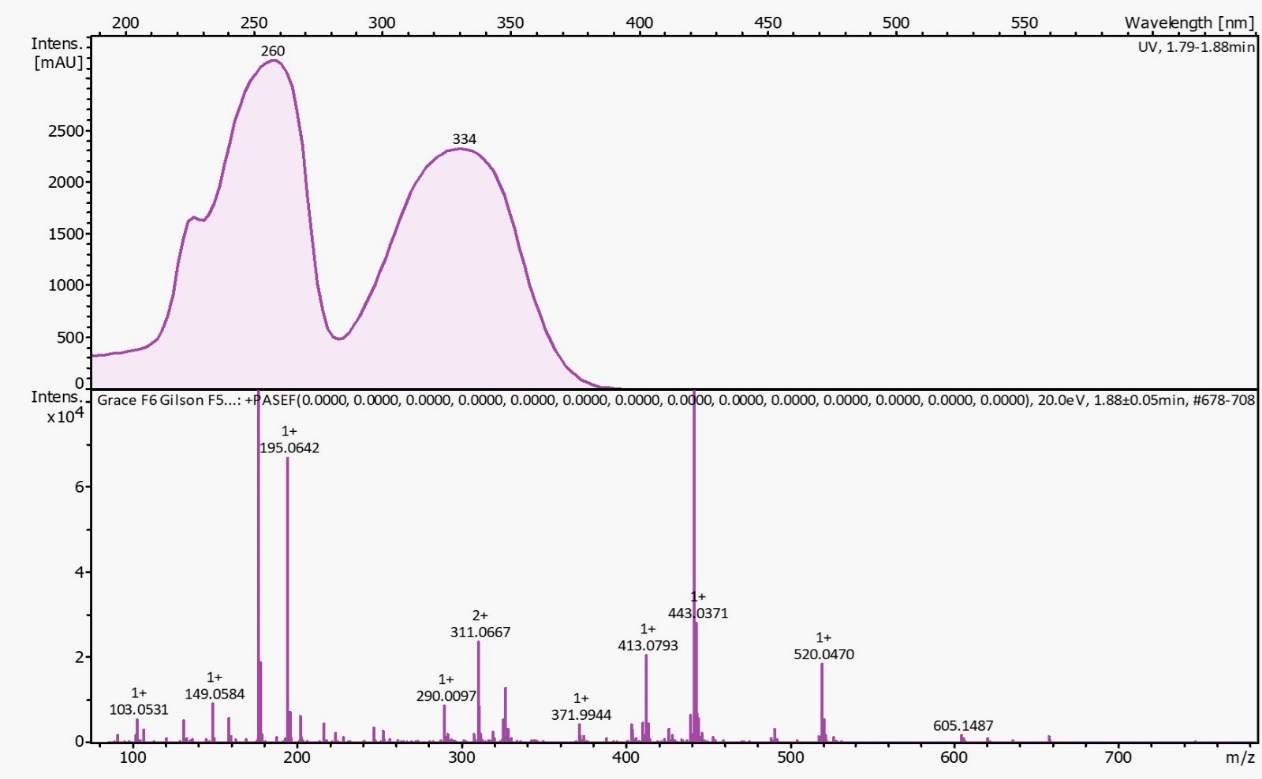


Figure S43. UV spectrum of 3,4-dihydro-3,4,8-trihydroxy-1(2H)-naphthalenone (7)


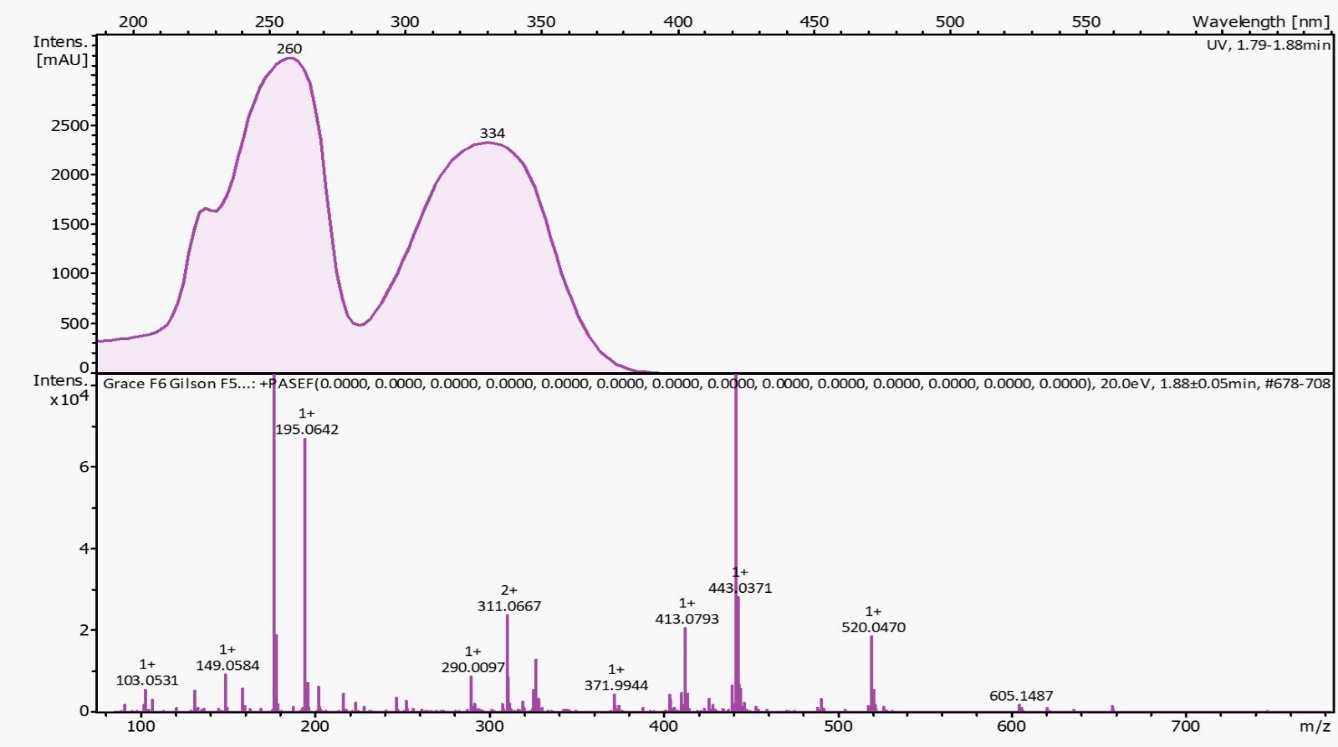


Figure S44. (+)-HR-ESI mass spectrum of 3,4-dihydro-3,4,8-trihydroxy-1(2H)-naphthalenone (7)

Figure S45. ^1^H NMR spectrum of 3,4-dihydro-3,4,8-trihydroxy-1(2H)-naphthalenone (7) (Acetone-*d₆*, 600 MHz)

Figure S46. ^13^C NMR spectrum of 3,4-dihydro-3,4,8-trihydroxy-1(2H)-naphthalenone (7) (Acetone-*d₆*, 150 MHz)

Figure S47. HSQC-DEPT spectrum of 3,4-dihydro-3,4,8-trihydroxy-1(2H)-naphthalenone (7)

Figure S48. HMBC spectrum of 3,4-dihydro-3,4,8-trihydroxy-1(2H)-naphthalenone (7)

Figure S49. COSY spectrum of 3,4-dihydro-3,4,8-trihydroxy-1(2H)-naphthalenone (7)

**Table S1.** Alignment used in the phylogenetic study.

>*Arnium*_*cirriferum*_CBS_120041

???????????????????????????????????????????????????????????????????????????????????????????????????????????????????????????????????????????????????????????????????????????????????????????????????????????????????????????????????????????????????????????????????????????????????????????????????????????????????????????????????????????????????????????????????????????????????????????????????????????????????????????????????????????????????????????????????????????????????????????????????????????????????????????????????????????????????????????????????????????????????????????????????????????????????????????????????????????????????????AAAGAAACCAACAGGG-ATTGCCCTAGTAACGGCGAGTGAAGCGGCAACAGCTCAAATTTGAAATCTGGCTCCGG-CCCGAGTTGTAATTTGCAGAGGAAGCTTCTGGTGATGCGCTGTCTAAGTCCCCTGGAACGGGGCGCCACAGTGGGTGAGAGCCCCATTT-GACAGAGGCAGATCCTGTGTGAAGCTCCTTCGACGAGTCGAGTAGTTTGGGAATGCTGCTCAAAATGGGAGGTAAATTCCTTCTAAAGCTAAATATTGGCCAGAGACCGATAGCGCACAAGTAGAGTGATC-------GAAAGATGAAAAGCACTTTGAAAAGAGGGTTAAACAGCACGTGAAATTGTTGAAAGGGAAGCGCTTGTGACCAGACTTGCGCTGGGCTGATCATCCGGTGTTCTCACCGGTGCACTCGGCCCAGCTCAGGCCAGCATCGGTTTTGGCGGGGGGATAAAGGCGTTAGGAACGTAGCTCCCTAGGGAGTGTTATAGCCCAGCGTGCAATGCCCCCGCTGGGACCGAGGTTCGCGCATCTGCAAGGATGCTGGCGTAATGGTCATCAGCGACCCGTCTTGAAACACGGACCAAGGAGTCAAGGTTTTGCGCGAGTGTTTGGGTGTCAAACCCGCACGCGTAATGAAAGTGAA-CGTAGGTGAGAG----CTTCGGCGCATCATCGACCGATCCTGATGTATTCGGATGGATTTGAGTAGGAGCGTTAAGCCTTGGACCCGAAAGATGGTGAACTATGCTTGGATAGGGTGAAGCCAGAGGAAACTCTGGTGGAGGCTCGCAGCGGTTCTGACGTGCAAATCGATCGTCAAATC-TGAGCATGGGGGCGAAAGACTAATCGAACCATCTAGTAGCTGGTTACCGCC??????????????????????????????????????????????????????????????????????????????????????????????????????????????????????????????????????????????????????????????????????????????????????????????????????????????????????????????????????????????????????????????????????????????????????????????????????????????????????????????????????????????????????????????????????????????????????????????????????????????????????????????????????????????????????????????????????????????????????????????????????????????????????????????????????????????????????????????????????????????????????????????????????????????????????????????????????????????????????????????????????????????????????????????????????????????????????????????????????????????????????????????????????????????????????????????????????????????????????????????????????????????????????????????????????????????????????????????????????????????????????????????????????????????????????????????????????????????????????????????????????GCTTGTTGACCAAGTTCTCGATGTCGTTCGTCGCGAGGCCGAGGGCTGCGACTGCCTCCAGGGCTTCCAGATCACCCACTCCCTCGGTGGTGGTACCGGTGCTGGTATGGGTACCCTCCTTATCTCCAAGATTCGCGAGGAGTTCCCCGACCGCATGATGGCGACCTTCTCTGTCGTCCCCTCCCCCAAGGTCTCTGACACCGTCGTCGAGCCCTACAACGCCACCCTGTCGGTGCACCAGCTTGTCGAGAACTCGGACGAGACCTTCTGCATTGACAACGAGGCTCTCTACGACATCTGCATGCGGACGCTCAAGCTGTCCAACCCCTCGTACGGCGACCTCAACCACCTAGTCTCCGCCGTCATGTCGGGTGTCACCGTCTCTCTCCGTTTCCCCGGCCAGCTCAACTCCGATCTCCGCAAGCTCGCCGTCAACATGGTTCCCTTCCCTCGTCTTCACTTCTTCATGGTCGGCTTCGCGCCCCTTACTAGCCGTGGCGCGCACTCTTTCCGTGCTGTCTCGGTTCCTGAGCTCACCCAGCAGATGTTCGACCCCAAGAACATGATGGCCGCCTCTGACTTCCGCAACGGTCGCTACCTCACCTGCTCTGCCATCTT

>*Immersiella*_*hirta*_E00204487

???????????????????????????????????????????????????????????????????????????????????????????????????????????????????????????????????????????????????????????????????????????????????????????????????????????????????????????????????????????????????????????????????????????????????????????????????????????????????????????????????????????????????????????????????????????????????????????????????????????????????????????????????????????????????????????????????????????????????????????????????????????????????????????????????????????????????????????????????????????????????????????????????????????????????????????????????????????????????????AAAGAAACCAACAGGG-ATTGCCCTAGTAACGGCGAGTGAAGCGGCAACAGCTCAAATTTGAAATCTGGCTTCGG-CCCGAGTTGTAATTTGCAGAGGAAGATTCTGGCGACGCGCCGTCCGAGTCCCCTGGAACGGGGCGCCATAGAGGGTGAGAGCCCCGTATGGACGGATGCCTAGCCTGTGTGAATCTCCTTCGACGAGTCGAGTAGTTTGGGAATGCTGCTCAAAATGGGAGGTAAATTCCTTCTAAAGCTAAATATTGGCCAGAGACCGATAGCGCACAAGTAGAGTGATC-------GAAAGATGAAAAGCACTTTGAAAAGAGGGTTAAACAGCACGTGAAATTGTTGAAAGGGAAGCGCTTGTGACCAGACTTGCGCCTGGCTGATCATCCGGTGTTCTCACCGGTGCACTCTGCCAGGCTCAGGCCAGCATCGGTTTTCGTGGGGGGATAAAGGTCCCGGGAATGTGGCTCCTCCGGGAGTGTTATAGCCCGGGGCGTAATGCCCTCGCGGGGACCGAGGTTCGCGCATCTGCAAGGATGCTGGCGTAATGGTCATCAGCGACCCGTCTTGAAACACGGACCAAGGAGTCAAGGTTTTGCGCGAGTGTTTGGGTGTCAAACCCGCACGCGTAATGAAAGTGAA-CGTAGGTGAGAG----CTTCGGCGCATCATCGACCGATCCTGATGTATTCGGATGGATTTGAGTAGGAGCGTTAAGCCTTGGACCCGAAAGATGGTGAACTATGCTTGGATAGGGTGAAGCCAGAGGAAACTCTGGTGGAGGCTCGCAGCGGTTCTGACGTGCAAATCGATCGTCAAATC-TGAGCATGGGGGCGAAAGACTAATCGAACCATCTAGTAGCTGGTTACCGCC??????????????????????????????????????????????????????????????????????????????????????????????????????????????????????????????????????????????????????????????????????????????????????????????????????????????????????????????????????????????????????????????????????????????????????????????????????????????????????????????????????????????????????????????????????????????????????????????????????????????????????????????????????????????????????????????????????????????????????????????????????????????????????????????????????????????????????????????????????????????????????????????????????????????????????????????????????????????????????????????????????????????????????????????????????????????????????????????????????????????????????????????????????????????????????????????????????????????????????????????????????????????????????????????????????????????????????????????????????????????????????????????????????????????????????????????????????????????????????????????????????GCTTGTTGACCAGGTTCTCGATGTCGTCCGTCGTGAGGCTGAGGGCTGCGACTGCCTCCAGGGCTTCCAGATCACCCACTCTCTCGGTGGTGGTACCGGTGCCGGTATGGGTACCCTCCTGATCTCCAAGATCCGCGAGGAGTTCCCCGACCGCATGATGGCCACTTTCTCGGTCGTCCCCTCTCCCAAGGTGTCGGATACCGTTGTTGAGCCCTACAACGCCACCCTCTCCGTTCATCAGCTCGTCGAGAACTCGGACGAGACCTTCTGCATTGACAACGAGGCTCTCTACGATATCTGCATGCGTACCCTGAAGCTGTCCAACCCCTCGTACGGTGACCTCAACCACCTGGTCTCGGCCGTCATGTCGGGTGTCACGGTTTCCCTGCGCTTCCCCGGCCAGCTCAACTCGGATCTCCGCAAGCTTGCCGTCAACATGGTTCCTTTCCCGCGTCTCCATTTCTTCATGGTCGGCTTCGCGCCTCTTACGAGCCGTGGCGCGCACTCTTTCCGTGCCGTCTCCGTTCCTGAGTTGACCCAGCAGATGTTTGACCCCAAGAACATGATGGCTGCCTCTGATTTCCGCAACGGTCGCTACCTGACTTGCTCTGCCATCTT

>*Immersiella*_*hirta*_E00204950

?????????????????????????????????????????????????????????????????????????????????????????????????????????????????????????????????????????????????????????????????????????????????????????????????????????????????????????????????????????????????????????????????????????????????????????????????????????????????????????????????????????????????????????????????????????????????????????????????????????????????????????????????????????????????????????????????????????????????????????????????????????????????????????????????????????????????????????????????????????????????????????????????????????????????????????????????????????????????????????????????????????????????????????????????????????????????????????????????????????????????????????????????GGCGACGCGCCGTCCGAGTCCCCTGGAACGGGGCGCCATAGAGGGTGAGAGCCCCGTATGGACGGATGCCTAGCCTGTGTGAATCTCCTTCGACGAGTCGAGTAGTTTGGGAATGCTGCTCAAAATGGGAGGTAAATTCCTTCTAAAGCTAAATATTGGCCAGAGACCGATAGCGCACAAGTAGAGTGATC-------GAAAGATGAAAAGCACTTTGAAAAGAGGGTTAAACAGCACGTGAAATTGTTGAAAGGGAAGCGCTTGTGACCAGACTTGCGCCTGGCTGATCATCCGGTGTTCTCACCGGTGCACTCTGCCAGGCTCAGGCCAGCATCGGTTTTCGTGGGGGGATAAAGGTCCCGGGAATGTGGCTCCTCCGGGAGTGTTATAGCCCGGGGCGTAATGCCCTCGCGGGGACCGAGGTTCGCGCATCTGCAAGGATGCTGGCGTAATGGTCATCAGCGACCCGTCTTGAAACACGGACCAAGGAGTCAAGGTTTTGCGCGAGTGTTTGGGTGTCAAACCCGCACGCGTAATGAAAGTGAA-CGTAGGTGAGAG----CTTCGGCGCATCATCGACCGATCCTGATGTATTCGGATGGATTTGAGTAGGAGCGTTAAGCCTTGGACCCGAAAGATGGTGAACTATGCTTGGATAGGGTGAAGCCAGAGGAAACTCTGGTGGAGGCTCGCAACGGTTCTGACGTGCAAATCGATCGTCAAATC-TGAGCATGGGGGCGAAAGACTAATCGAACCATCTAGTAGCTGGTTACCGCC??????????????????????????????????????????????????????????????????????????????????????????????????????????????????????????????????????????????????????????????????????????????????????????????????????????????????????????????????????????????????????????????????????????????????????????????????????????????????????????????????????????????????????????????????????????????????????????????????????????????????????????????????????????????????????????????????????????????????????????????????????????????????????????????????????????????????????????????????????????????????????????????????????????????????????????????????????????????????????????????????????????????????????????????????????????????????????????????????????????????????????????????????????????????????????????????????????????????????????????????????????????????????????????????????????????????????????????????????????????????????????????????????????????????????????????????????????????????????????????????????????GCTTGTTGACCAGGTTCTCGATGTCGTCCGTCGTGAGGCTGAGGGCTGCGACTGCCTCCAGGGCTTCCAGATCACCCACTCTCTCGGTGGTGGTACCGGTGCCGGTATGGGTACCCTCCTGATCTCCAAGATCCGCGAGGAGTTCCCCGACCGCATGATGGCCACTTTCTCGGTCGTCCCCTCTCCCAAGGTGTCGGATACCGTTGTTGAGCCCTACAACGCCACCCTCTCCGTTCATCAGCTCGTCGAGAACTCGGACGAGACCTTCTGCATTGACAACGAGGCTCTCTACGATATCTGCATGCGTACCCTGAAGCTGTCCAACCCCTCGTACGGTGACCTCAACCACCTGGTCTCGGCCGTCATGTCGGGTGTCACGGTTTCCCTGCGCTTCCCCGGCCAGCTCAACTCGGATCTCCGCAAGCTTGCCGTCAACATGGTTCCTTTCCCGCGTCTCCATTTCTTCATGGTCGGCTTCGCGCCTCTTACGAGCCGTGGCGCGCACTCTTTCCGTGCCGTCTCCGTTCCTGAGTTGACCCAGCAGATGTTTGACCCCAAGAACATGATGGCTGCCTCTGATTTCCGCAACGGTCGCTACCTGACTTGCTCTGCCATCTT

>*Pseudoschizothecium*_*atropurpureum*_SMH2961

???????????????????????????????????????????????????????????????????????????????????????????????????????????????????????????????????????????????????????????????????????????????????????????????????????????????????????????????????????????????????????????????????????????????????????????????????????????????????????????????????????????????????????????????????????????????????????????????????????????????????????????????????????????????????????????????????????????????????????????????????????????????????????????????????????????????????????????????????????????????????????????????????????????????????????????????????????????????????????AAAGAAACCAACAGGG-ATTGCCCTAGTAACGGCGAGTGAAGCGGCAACAGCTCAAATTTGAAATCTGGCTCCGG-CCCGAGTTGTAATTTGCAGAGGAAGCATTTGGTGCGGTATTCTCCGAGTCCCCTGGAATGGGGCGCCAGAGAGGGTGAGAGCCCCGTAGGATTGAATACCTAGCCTGTGTGAAGCTCCTTCGACGAGTCGAGTAGTTTGGGAATGCTGCTCTAAATGGGAGGTAAATTCCTTCTAAAGCTAAATATTGGCCAGAGACCGATAGCGCACAAGTAGAGTGATC-------GAAAGATGAAAAGCACTTTGAAAAGAGGGTTAAATAGCACGTGAAATTGTTGAAAGGGAAGCGCCTGTGACCAGACTTGCGCCCAGCGGATCATCCGGTGTTCTCACCGGTGCACTCCGCTTGGCTCAGGCCAGCATCGGTTCTGGTGGGGGGATAAAGGTTCCGGGAATGTAGCTCTTTCGGGAGTGTTATAGCCCGGGGCGCAATGCCCTCGCCGGGACCGAGGACCGCGCGAAAGCAAGGATGCTGGCGTAATGGTCACCAGCGACCCGTCTTGAAACACGGACCAAGGAGTCAAGGTTTTGCGCGAGTGTTTGGGTGTCAAACCCGCACGCGTAATGAAAGTGAA-CGTAGGTGAGAG----CTTCGGCGCATCATCGACCGATCCTGATGTACTCGGATGGATTTGAGTAGGAGCGTTAAGCCTTGGACCCGAAAGATGGTGAACTATGCTTGGATAGGGTGAAGCCAGAGGAAACTCTGGTGGAGGCTCGCAGCGGTTCTGACGTGCAAATCGATCGTCAAATC-TGAGCATGGGGGCGAAAGACTAATCGAACCATCTAGTAGCTGGTTACCGCC??????????????????????????????????????????????????????????????????????????????????????????????????????????????????????????????????????????????????????????????????????????????????????????????????????????????????????????????????????????????????????????????????????????????????????????????????????????????????????????????????????????????????????????????????????????????????????????????????????????????????????????????????????????????????????????????????????????????????????????????????????????????????????????????????????????????????????????????????????????????????????????????????????????????????????????????????????????????????????????????????????????????????????????????????????????????????????????????????????????????????????????????????????????????????????????????????????????????????????????????????????????????????????????????????????????????????????????????????????????????????????????????????????????????????????????????????????????????????????????????????????GCTTGTTGACCAAGTTCTCGACGTCGTCCGCCGTGAGGCCGAGGGCTGCGACTGCCTCCAGGGTTTCCAGATCACCCACTCGCTCGGTGGTGGTACTGGTGCTGGTATGGGTACCCTTCTCATCTCCAAGATTCGCGAGGAGTTCCCCGACCGCATGATGGCGACTTTCTCCGTCGTCCCCTCGCCCAAGGTGTCCGACACCGTCGTCGAGCCCTACAACGCTACCCTCTCCGTTCACCAGCTCGTTGAGAACTCGGACGAGACCTTCTGTATTGATAACGAGGCTCTGTACGATATCTGCATGCGCACCCTGAAGCTGTCCAACCCCTCGTATGGCGACCTCAACCACCTCGTTTCGGCTGTCATGTCGGGCGTCACGGTCTCGCTCCGTTTCCCCGGCCAGCTTAACTCGGATCTCCGCAAGCTCGCCGTCAACATGGTGCCTTTCCCTCGTCTGCACTTCTTCATGGTTGGCTTTGCGCCCCTGACTAGCCGCGGCGCCTACACTTTCCGTGCCGTCTCGGTTCCGGAGCTCACCCAGCAGATGTTCGACCCCAAGAACATGATGGCTGCTTCTGACTTCCGCAATGGCCGCTACCTCACTTGCTCAGCCATTTT

>*Pseudoschizothecium*_*atropurpureum*_SMH3073

???????????????????????????????????????????????????????????????????????????????????????????????????????????????????????????????????????????????????????????????????????????????????????????????????????????????????????????????????????????????????????????????????????????????????????????????????????????????????????????????????????????????????????????????????????????????????????????????????????????????????????????????????????????????????????????????????????????????????????????????????????????????????????????????????????????????????????????????????????????????????????????????????????????????????????????????????????????????????????AAAGAAACCAACAGGG-ATTGCCCTAGTAACGGCGAGTGAAGCGGCAACAGCTCAAATTTGAAATCTGGCTCCGG-CCCGAGTTGTAATTTGCAGAGGAAGCATTTGGTGCGGTATTCTCCGAGTCCCCTGGAATGGGGCGCCAGAGAGGGTGAGAGCCCCGTAGGATTGAATACCTAGCCTGTGTGAAGCTCCTTCGACGAGTCGAGTAGTTTGGGAATGCTGCTCTAAATGGGAGGTAAATTCCTTCTAAAGCTAAATATTGGCCAGAGACCGATAGCGCACAAGTAGAGTGATC-------GAAAGATGAAAAGCACTTTGAAAAGAGGGTTAAATAGCACGTGAAATTGTTGAAAGGGAAGCGCCTGTGACCAGACTTGCGCCCAGCGGATCATCCGGTGTTCTCACCGGTGCACTCCGCTTGGCTCAGGCCAGCATCGGTTCTGGTGGGGGGATAAAGGTTCCGGGAATGTAGCTCTTTCGGGAGTGTTATAGCCCGGGGCGCAATGCCCTCGCCGGGACCGAGGACCGCGCGAAAGCAAGGATGCTGGCGTAATGGTCACCAGCGACCCGTCTTGAAACACGGACCAAGGAGTCAAGGTTTTGCGCGAGTGTTTGGGTGTCAAACCCGCACGCGTAATGAAAGTGAA-CGTAGGTGAGAG----CTTCGGCGCATCATCGACCGATCCTGATGTACTCGGATGGATTTGAGTAGGAGCGTTAAGCCTTGGACCCGAAAGATGGTGAACTATGCTTGGATAGGGTGAAGCCAGAGGAAACTCTGGTGGAGGCTCGCAGCGGTTCTGACGTGCAAATCGATCGTCAAATC-TGAGCATGGGGGCGAAAGACTAATCGAACCATCTAGTAGCTGGTTACCGCCGCTCTGAAATACTCACTCGCGACTGGCAATTGGGGAGATCAAAAGAAAGCAATGAACTCGACCGCCGGTGTCTCTCAAGTTTTGAACAGATATACGTTTGCTTCGACCCTCTCTCATTTGCGTCGGACAAATACACCCATCGGACGTGATGGCAAGCTTGCCAAACCCCGCCAGCTTCACAACACGCACTGGGGCTTAGTTTGCCCCGCCGAGACCCCAGAAGGACAGGCTTGCGGTTTGGTCAAGAATCTATCGCTCATGTGTTACGTCAGTGTTGGCACCAATGCCGAACCGATTGTCGACTTCATGGTGCAGAGAGGCATGGAAGTGCTGGAAGAATACGAGCCCTTGCGATATCCAAATTCCACCAAGGTTTTCGTCAACGGGACTTGGGTGGGTGTCCACCCCGATCCAAAGCACCTGGTTACCGCGGTCCAGGGTCTACGGCGTAGCAACCTTATATCATTCGAGGTTTCGCTCGTGAGAGATATCAGGGACCGCGAGTTCCAAATCTTCTCCGATGCTGGCCGTGTCATGAGGCCGTTATTTGTGGTAGAGCAGGAAGACAGTGGCGAA---------CATGGAGTTGAGAAGGGCCAGCTGGTTCTCACGAAATCCATGGTCCGCGAGCTTGAACGAAGCAAGGGGCTGGGCAAGTTCCACCCTGATTACAAGGGCTGGGAGTGGCTCCTCGGGCAGGGAGCTATTGAGTATTTGGATGCCGAAGAGGAGGAAACGGCCATGATCTGCATGACTCCAGAGGATCTCGACAATTATCGGCTCACTCGTCT------------GGGCTTGGAGATGCCCGAAGAAGA--GGAG-GGGAACGCCAAGCGTATCAAGACGAGGATGAACCCGACGACGCACATGTATACGCATTGCGAAATCCACCCGGCTATGCTGCTGGGAATTTGCGCCAGCATTATCCCCTTCCCGGATCACAACCAAGCTTGTTGACCAAGTTCTCGACGTCGTCCGCCGTGAGGCCGAGGGCTGCGACTGCCTCCAGGGTTTCCAGATCACCCACTCGCTCGGTGGTGGTACTGGTGCTGGTATGGGTACCCTTCTCATCTCCAAGATTCGCGAGGAGTTCCCCGACCGCATGATGGCGACTTTCTCCGTCGTCCCCTCGCCCAAGGTGTCCGACACCGTCGTCGAGCCCTACAACGCTACCCTCTCCGTTCACCAGCTCGTTGAGAACTCGGACGAGACCTTCTGTATTGATAACGAGGCTCTGTACGATATCTGCATGCGCACCCTGAAGCTGTCCAACCCCTCGTATGGCGACCTCAACCACCTCGTTTCGGCTGTCATGTCGGGCGTCACGGTCTCGCTCCGTTTCCCCGGCCAGCTTAACTCGGATCTCCGCAAGCTCGCCGTCAACATGGTGCCTTTCCCTCGTCTGCACTTCTTCATGGTTGGCTTTGCGCCCCTGACTAGCCGCGGCGCCTACACTTTCCGTGCCGTCTCGGTTCCGGAGCTCACCCAGCAGATGTTCGACCCCAAGAACATGATGGCTGCTTCTGACTTCCGCAATGGCCGCTACCTCACTTGCTCAGCCATTTT

>*Cercophora*_*newfieldiana*_SMH3303

?????????????????????????????????????????????????????????????????????????????????????????????????????????????????????????????????????????????????????????????????????????????????????????????????????????????????????????????????????????????????????????????????????????????????????????????????????????????????????????????????????????????????????????????????????????????????????????????????????????????????????????????????????????????????????????????????????????????????????????????????????????????????????????????????????????????????????????????????????????????????????????????????????????????????????????????????????????????????????????????????????GG-ATTGCCCTAGTAACGGCGAGTGAAGCGGCAACAGCTCAAATTTGAAATCTGGCTCCGG-CCCGAGTTGTAATTTGCAGAGGAAGCTTCTGGTGATATACTGTCTAAGTCCCCTGGAACGGGGCGCCACAGTGGGTGAGAGCCCCATAT-GACAGCTGTAGATCCTGTGTGAAGCTCCTTCGACGAGTCGAGTAGTTTGGGAATGCTGCTCTAAATGGGAGGTAAATTCCTTCTAAAGCTAAATATTGGCCAGAGACCGATAGCGCACAAGTAGAGTGATC-------GAAAGATGAAAAGCACTTTGAAAAGAGAGTTAAACAGCACGTGAAATTGTTGAAAGGGAAGCGCTTATGACCAGACTTGCGCTGGGCTAATCATCCGGTGTTCTCACCGGTGCACTTGGCCCAGCTCAGGCCAGCATCGGTTTTGGTGGGGGGATAAAGGCGCTGGGAACGTAGCTCTTTCGGGAGTGTTATAGCCCAGTGTGCAATGCCCCCGCTGGGACCGAGGTTCGCGCATTTGCAAGGATGCTGGCGTAATGGTCATCAGCGACCCGTCTTGAAACACGGACCAAGGAGTCAAGGTTTTGCGCGAGTGTTTGGGTGTCAAACCCGCACGCGTAATGAAAGTGAA-CGTAGGTGAGAG----CTTCGGCGCATCATCGACCGATCCTGATGTATTCGGATGGATTTGAGTAGGAGCGTTAAGCCTTGGACCCGAAAGATGGTGAACTATGCTTGGATAGGGTGAAGCCAGAGGAAACTCTGGTGGAGGCTCGCAGCGGTTCTGACGTGCAAATCGATCGTCAAATC-TGAGCATGGGGGCGAAAGACTAATCGAACCATCTAGTAGCTGGTTACCGCCGCCCTCAAGTACTCGCTGGCTACCGGCAACTGGGGTGATCAGAAGAAGGCGATGAGCTCTACGGCTGGTGTGTCGCAGGTGTTGAACCGATATACATTTGCCTCGACGCTCTCCCATTTGCGGCGAACAAACACACCTATCGGTCGTGACGGCAAGCTTGCCAAACCTCGACAGCTGCACAACACTCACTGGGGCCTCGTCTGCCCTGCCGAAACCCCAGAAGGCCAAGCTTGCGGCCTCGTCAAGAACCTCTCTCTGATGTGCTATATTAGCGTGGGCACTAATGCGGAACCTATCGTCGACTTTATGGTGGCTAGGAACATGGAAGTCCTCGAAGAGTACGAGCCGCTCCGCTATCCCAACGCCACGAAGGTCTTCGTCAACGGAACCTGGGTTGGCGTGCACCAAGACCCCAAGCATTTGGTGACCCTGGTCCAGAATCTCAGGAGGTCCAACATCATCTCCTTTGAAGTTTCGCTTGTCCGGGACATTCGAGATCGAGAGTTCAAGATCTTCTCCGACGCAGGCCGTGTCATGAGGCCGCTCTTTGTTGTTGAGCAAGAAGACGAGAACAAG------GTGACCAAGGTCCAGAAGGGCCAGTTGGTCTTGACAAGGTCGCACATCGACCGGCTGGATCGGGACAAGGAGCTCGGGCCATTGGACGAAGGTTTCTTTGGTTGGAACGGCCTCCTCAGAGAGGGTTGTGTCGAGTATCTCGACGCCGAGGAAGAGGAGACGGCCATGATTTGCATGACGCCCGAAGACTTGGATCACTACCGATCTACCAAGCTGGGC--GTTAAGTCGAAAGCCCACCTA-GACGATGAAGAGGAAGGGCACAACAAGCGCATCAAAACGAAGGCGAACCCGACCACTCATATGTACACTCATTGCGAGATCCATCCCAGTATGCTGCTTGGTATCTGCGCGAGCATTATCCCCTTCCCGGATCACAATCAGGCTTGTTGACCAAGTTCTCGATGTCGTCCGTCGTGAGGCCGAGGGCTGCGACTGCCTCCAGGGCTTCCAGATCACCCACTCGCTCGGTGGTGGTACCGGTGCCGGTATGGGTACCCTCCTTATCTCCAAGATTCGCGAGGAGTTCCCCGACCGCATGATGGCGACTTTTTCCGTCGTTCCCTCCCCCAAGGTCTCGGACACCGTCGTTGAGCCATACAACGCCACCCTGTCGGTTCACCAGCTTGTCGAGAACTCGGACGAGACTTTCTGCATTGACAACGAGGCTCTGTACGATATCTGCATGCGGACGCTGAAGCTTTCCAACCCCTCGTACGGCGACCTCAACCACCTGGTCTCCGCCGTCATGTCGGGTGTCACCGTTTCCCTGCGTTTCCCCGGCCAGCTCAACTCCGATCTTCGCAAGCTTGCCGTCAACATGGTTCCCTTCCCTCGTCTGCACTTCTTCATGGTCGGCTTCGCGCCTCTTACTAGCCGTGGCGCGTACACTTTCCGTGCCGTCTCGGTGCCCGAGCTCACCCAGCAGATGTTCGACCCCAAGAACATGATGGCTGCCTCCGACTTCCGCAACGGTCGCTATCTGACTTGCTCGGCCATTTT

>*Echria*_*gigantospora*_F77_1

???????????????????????????????????????????????????????????????????????????????????????????????????????????????????????????????????????????????????????????????????????????????????????????????????????????????????????????????????????????????????????????????????????????????????????????????????????????????????????????????????????????????????????????????????????????????????????????????????????????????????????????????????????????????????????????????????????????????????????????????????????????????????????????????????????????????????????????????????????????????????????????????????????????????????????????????????????????????????????AAAGAAACCAACAGGG-ATTGCCCTAGTAACGGCGAGTGAAGCGGCAACAGCTCAAATTTGAAATCTGGCCTCGG-CCCGAGTTGTAATTTGCAGAGGAAGCTTCTGGCGCGGCGCTGTCCGAGTCCCCTGGAACGGGGCGCCATAGAGGGTGAGAGCCCCGTATGGATGGATGCCTAGCCTGTGTGAAGCTCCTTCGACGAGTCGAGTAGTTTGGGAATGCTGCTCAAAATGGGAGGTAAATTCCTTCTAAAGCTAAATATTGGCCAGAGACCGATAGCGCACAAGTAGAGTGATC-------GAAAGATGAAAAGCACTTTGAAAAGAGGGTTAAATAGCACGTGAAATTGTTGAAAGGGAAGCGCTTGTGACCAGACTTGCGCCAGGCTGATCATCCGGTGTTCTCACCGGTGCACTCTGCCTGGCTCAGGCCAGCATCGGTTTCAGCGGGGGGATAAAGGCCTAGGGAACGTAGCTCCCCCGGGAGTGTTATAGCCCTGGGTGCAATGCCCCCGCCGGGACCGAGGTTCGCGCATCTGCTAGGATGCTGGCGTAATGGTCATCAGCGACCCGTCTTGAAACACGGACCAAGGAGTCAAGGTTTTGCGCGAGTGTTTGGGTGTTAAACCCGCACGCGTAATGAAAGTGAA-CGTAGGTGAGAG----CTTCGGCGCATCATCGACCGATCCTGATGTATTCGGATGGATTTGAGTAGGAGCGTTAAGCCTTGGACCCGAAAGATGGTGAACTATGCTTGGATAGGGTGAAGCCAGAGGAAACTCTGGTGGAGGCTCGCAGCGGTTCTGACGTGCAAATCGATCGTCAAATC-TGAGCATGGGGGCGAAAGACTAATCGAACCATCTAGTAGCTGGTTACCGCC??????????????????????????????????????????????????????????????????????????????????????????????????????????????????????????????????????????????????????????????????????????????????????????????????????????????????????????????????????????????????????????????????????????????????????????????????????????????????????????????????????????????????????????????????????????????????????????????????????????????????????????????????????????????????????????????????????????????????????????????????????????????????????????????????????????????????????????????????????????????????????????????????????????????????????????????????????????????????????????????????????????????????????????????????????????????????????????????????????????????????????????????????????????????????????????????????????????????????????????????????????????????????????????????????????????????????????????????????????????????????????????????????????????????????????????????????????????????????????????????????????ACTTGTTGACCAAGTTCTCGATGTCGTTCGTCGTGAGGCTGAGGGCTGCGACTGCCTTCAGGGTTTCCAGATCACCCACTCGCTCGGTGGTGGTACCGGTGCCGGTATGGGTACCCTCCTTATCTCCAAGATTCGTGAGGAGTTCCCCGACCGCATGATGGCGACTTTCTCAGTCGTGCCGTCGCCCAAGGTCTCGGATACCGTTGTCGAGCCCTACAATGCCACTCTGTCGGTGCACCAGCTTGTCGAGAACTCGGACGAGACCTTCTGCATTGATAACGAGGCTCTCTACGATATCTGCATGCGGACGCTCAAGCTGTCGAACCCCTCGTACGGCGATCTGAACCACCTCGTTTCGGCCGTCATGTCCGGCGTCACCGTTTCTCTCCGTTTCCCTGGCCAGCTCAACTCTGATCTGCGCAAGCTTGCGGTGAACATGGTTCCCTTCCCTCGTCTCCACTTCTTCATGGTCGGCTTCGCGCCACTGACGAGCCGTGGCGCCCACTCCTTCCGCGCTGTCTCTGTGCCGGAGCTTACGCAGCAGATGTTCGACCCCAAGAACATGATGGCTGCTTCGGACTTCCGCAATGGCCGGTACCTTACTTGCTCGGCCATCTT

>*Echria*_*macrotheca*_Lundqvist_2311

???????????????????????????????????????????????????????????????????????????????????????????????????????????????????????????????????????????????????????????????????????????????????????????????????????????????????????????????????????????????????????????????????????????????????????????????????????????????????????????????????????????????????????????????????????????????????????????????????????????????????????????????????????????????????????????????????????????????????????????????????????????????????????????????????????????????????????????????????????????????????????????????????????????????????????????????????????????????????????AAAGAAACCAACAGGG-ATTGCCCTAGTAACGGCGAGTGAAGCGGCAACAGCTCAAATTTGAAATCTGGCCCCGG-CCCGAGTTGTAATTTGCAGAGGAAGCTTCTGGCGCGGCGCCGTCCGAGTCCCCTGGAACGGGGCGCCATAGAGGGTGAGAGCCCCGTATGGATGGACGCCTAGCCTGTGTGAAGCTCCTTCGACGAGTCGAGTAGTTTGGGAATGCTGCTCAAAATGGGAGGTAAATTCCTTCTAAAGCTAAATATTGGCCAGAGACCGATAGCGCACAAGTAGAGTGATC-------GAAAGATGAAAAGCACTTTGAAAAGAGGGTTAAATAGCACGTGAAATTGTTGAAAGGGAAGCGCTTGTGACCAGACTTGCGCCAGGCTGATCATCCGGTGTTCTCACCGGTGCACTCTGCCTGGCTCAGGCCAGCATCGGTTTCAGCGGGGGGATAAAGGCCTAGGGAACGTAGCTCCCCCGGGAGTGTTATAGCCCTGGGTGCAATGCCCCCGCCGGGACCGAGGTTCGCGCATCTGCTAGGATGCTGGCGTAATGGTCATCAGCGACCCGTCTTGAAACACGGACCAAGGAGTCAAGGTTTTGCGCGAGTGTTTGGGTGTTAAACCCGCACGCGTAATGAAAGTGAA-CGTAGGTGAGAG----CTTCGGCGCATCATCGACCGATCCTGATGTATTCGGATGGATTTGAGTAGGAGCGTTAAGCCTTGGACCCGAAAGATGGTGAACTATGCTTGGATAGGGTGAAGCCAGAGGAAACTCTGGTGGAGGCTCGCAGCGGTTCTGACGTGCAAATCGATCGTCAAATC-TGAGCATGGGGGCGAAAGACTAATCGAACCATCTAGTAGCTGGTTACCGCC??????????????????????????????????????????????????????????????????????????????????????????????????????????????????????????????????????????????????????????????????????????????????????????????????????????????????????????????????????????????????????????????????????????????????????????????????????????????????????????????????????????????????????????????????????????????????????????????????????????????????????????????????????????????????????????????????????????????????????????????????????????????????????????????????????????????????????????????????????????????????????????????????????????????????????????????????????????????????????????????????????????????????????????????????????????????????????????????????????????????????????????????????????????????????????????????????????????????????????????????????????????????????????????????????????????????????????????????????????????????????????????????????????????????????????????????????????????????????????????????????????GCTTGTTGACCAAGTTCTCGATGTCGTCCGTCGTGAGGCCGAGGGCTGCGACTGCCTCCAGGGTTTCCAGATCACGCACTCGCTCGGTGGAGGTACCGGTGCCGGTATGGGTACCCTCCTTATCTCCAAGATTCGTGAGGAGTTCCCCGACCGCATGATGGCGACTTTCTCGGTCGTGCCGTCGCCCAAGGTCTCGGATACCGTTGTCGAGCCCTACAACGCCACTCTGTCGGTGCACCAGCTTGTCGAGAACTCGGACGAGACGTTCTGCATTGATAACGAGGCTCTCTACGATATCTGCATGCGGACGCTCAAGCTGTCGAACCCCTCATACGGCGACCTGAACCACCTCGTTTCGGCCGTCATGTCGGGCGTCACCGTTTCTCTCCGTTTCCCTGGCCAGCTCAACTCTGATCTCCGCAAGCTTGCGGTGAACATGGTTCCCTTCCCTCGTCTCCACTTCTTCATGGTGGGCTTCGCGCCGCTGACGAGCCGTGGCGCCCACTCTTTCCGCGCTGTCTCTGTGCCGGAGCTTACGCAGCAGATGTTCGACCCCAAGAACATGATGGCTGCTTCGGACTTCCGCAATGGCCGTTACCTTACTTGCTCGGCCATCTT

>*Schizothecium*_*curvisporum*_ATCC_36709

???????????????????????????????????????????????????????????????????????????????????????????????????????????????????????????????????????????????????????????????????????????????????????????????????????????????????????????????????????????????????????????????????????????????????????????????????????????????????????????????????????????????????????????????????????????????????????????????????????????????????????????????????????????????????????????????????????????????????????????????????????????????????????????????????????????????????????????????????????????????????????????????????????????????????????????????????????????????????????AAAGAAACCAACAGGG-ATTGCCCCAGTAACGGCGAGTGAAGCGGCAACAGCTCAAATTTGAAATCTGGCCTCGG-CCCGAGTTGTAATTTGCAGAGGAAGCTTCTGGTGCGGCGCCGTCCGAGTCTCCTGGAACGGAGCGCCATAGAGGGTGAGAGCCCCGTATGGACGGACGCCAAACCTGTGTGAAGCTCCTTCGACGAGTCGAGTAGTTTGGGAATGCTGCTCAAAATGGGAGGTAAATTCCTTCTAAAGCTAAATACCGGCCAGAGACCGATAGCGCACAAGTAGAGTGATC-------GAAAGATGAAAAGCACTTTGAAAAGAGGGTTAAACAGCACGTGAAATTGTTGAAAGGGAAGCGCTTGTGACCAGACTCGGGCGCGGCGGATCATCCGGTGTTCTCACCGGTGCACTCCGCCGCGCCCGGGCCAGCATCGGCTTCCGCCGGGGGACAAAGGTCCCGGGAACGTAGCTCCTCCGGGAGTGTTATAGCCCGGGGCGCAATGCCCCGGCGGGGGCCGAGGACCGCGCAT-TGCAAGGATGCTGGCGTAATGGTCATCAGCGACCCGTCTTGAAACACGGACCAAGGAGTCAAGGTTTTGCGCGAGTGTTTGGGTGTTAAACCCGCACGCGTAATGAAAGTGAA-CGTAGGTGAGAG----CTTCGGCGCATCATCGACCGATCCTGATGTATTCGGATGGATTTGAGTAGGAGCGTTAAGCCTTGGACCCGAAAGATGGTGAACTATGCTTGGATAGGGTGAAGCCAGAGGAAACTCTGGTGGAGGCTCGCAGCGGTTCTGACGTGCAAATCGATCGTCAAATC-TGAGCATGGGGGCGAAAGACTAATCGAACCATCTAGTAGCTGGTTACCGCCGCGCTCAAGTACTCGCTTGCCACGGGTAACTGGGGCGACCAGAAGAAGGCGGCGAGCTCGACAGCCGGTGTGTCCCAGGTGTTGAATCGCTACACGTTTGCCTCGACCCTTTCTCATTTGCGCCGCACCAACACGCCCATCGGCCGCGACGGAAAGCTGGCCAAGCCGCGCCAGCTGCACAACACCCATTGGGGCCTCGTCTGTCCAGCCGAGACGCCCGAGGGCCAGGCCTGCGGGCTGGTCAAGAATCTGTCGCTCATGTGCTACATTAGCGTGGGCACCAACGCGGAGCCCATTATCGAGTTCATGATCGCGCGCAACATGGAGGTCTTGGAAGAGTACGAGCCACTGCGCTCCCCCAACGCCACCAAGATCTTTGTCAACGGCACGTGGGTCGGCGTGCACCACGACGCCAAGCACCTCGTGCACCTTGTCCAGGGTCTCCGGCGATCCAACATTGTGAGCTTCGAGGTGTCGCTGGTCCGGGATATCCGAGACCGCGAGTTCAAGATCATGTCGGATGCCGGCCGCGTCATGAGGCCCCTCTTCGTCGTCGAGACCGAGGACGAGAGCTCC---------ACCGGGGTGGAAAAGGGCGAGCTGGTGCTCACCAAGACCCACGTCCAGAAGCTGGCCAACGACAAGCTGATTGGGAAATACCACAAAGACTACTTTGGGTGGCAAGGCCTCTTGCAATCAGGTGCCGTCGAATACCTCGACGCCGAGGAGGAGGAGACGGCCATGATCTCCATGTCGCCCGAAGACCTCGACCATTTCCGCGACGCCAAGGC------GAGAAACTTTGAGGAACCCGAGGGCAAGGTGGTGACCGAGGGCAACAAGCGCATCCCGACGAGGATCAACCCGACGACCTACATGTATACGCACTGCGAGATCCACCCGAGCATGCTGCTCGGCATCTGCGCGAGCATCATCCCTTTCCCGGACCACAACCAGGCTTGTCGACCAGGTTCTCGATGTCGTCCGTCGCGAGGCCGAGGGCTGCGACTGCCTCCAGGGCTTCCAGATCACCCACTCGCTCGGTGGTGGTACCGGTGCCGGTATGGGTACCCTCCTCATCTCCAAGATCCGCGAGGAGTTCCCCGACCGCATGATGGCGACCTTCTCCGTCGTCCCCTCCCCCAAGGTGTCGGATACCGTCGTGGAGCCCTACAACGCCACCCTCTCCGTCCACCAGCTTGTCGAGAACTCGGACGAGACCTTCTGCATTGACAACGAGGCTCTGTACGACATCTGCATGCGCACCCTCAAGCTGTCGAACCCCTCGTACGGCGACCTCAACCACCTCGTCTCGGCCGTCATGTCGGGTGTCACCGTTTCGCTGCGCTTCCCCGGCCAGCTCAACTCTGATCTCCGCAAGCTCGCCGTGAACATGGTTCCCTTCCCCCGTCTCCACTTCTTCATGGTCGGCTTCGCCCCTCTTACCAGCCGTGGCGCCCACTCTTTCCGTGCCGTCTCGGTCCCCGAGCTCACCCAGCAGATGTTCGACCCCAAGAACATGATGGCTGCCTCGGACTTCCGCAACGGTCGTTACCTTACCTGCTCCGCCATCTT

>*Immersiella*_*caudata*_SMH3298

??????????????????????????????????????????????????????????????????????????????????????????????????????????????????????????????????????????????????????????????????????????????????????????????????????????????????????????????????????????????????????????????????????????????????????????????????????????????????????????????????????????????????????????????????????????????????????????????????????????????????????????????????????????????????????????????????????????????????????????????????????????????????????????????????????????????????????????????????????????????????????????????????????????????????????????????????????????????????????????????CCAACAGGG-ATTGCCCTAGTAACGGCGAGTGAAGCGGCAACAGCTCAAATTTGAAATCTGGCTTCGG-CCCGAGTTGTAATTTGCAGAGGAAGATTCTGGCGACGCGCCGTCCGAGTCCCCTGGAACGGGGCGCCATAGAGGGTGAGAGCCCCGTATGGATGGATGCCTAGCCTGTGTGAATCTCCTTCGACGAGTCGAGTAGTTTGGGAATGCTGCTCAAAATGGGAGGTAAATTCCTTCTAAAGCTAAATATTGGCCAGAGACCGATAGCGCACAAGTAGAGTGATC-------GAAAGATGAAAAGCACTTTGAAAAGAGGGTTAAACAGCACGTGAAATTGTTGAAAGGGAAGCGCTTGTGACCAGACTTGCGCCTGGCTGATCATCCGGTGTTCTCACCGGTGCACTCTGCCAGGCTCAGGCCAGCATCGGTTTTCGTGGGGGGATAAAGGTCCCGGGAACGTGGCTCCTCCGGGAGTGTTATAGCCCGGGGCGTAATGCCCTCGCGGGGACCGAGGTTCGCGCATCTGCAAGGATGCTGGCGTAATGGTCATCAGCGACCCGTCTTGAAACACGGACCAAGGAGTCAAGGTTTTGCGCGAGTGTTTGGGTGTTAAACCCGCACGCGTAATGAAAGTGAA-CGTAGGTGAGAG----CTTCGGCGCATCATCGACCGATCCTGATGTATTCGGATGGATTTGAGTAGGAGCGTTAAGCCTTGGACCCGAAAGATGGTGAACTATGCTTGGATAGGGTGAAGCCAGAGGAAACTCTGGTGGAGGCTCGCAGCGGTTCTGACGTGCAAATCGATCGTCAAATC-TGAGCATGGGGGCGAAAGACTAATCGAACCATCTAGTAGCTGGTTACCGCCGCTCTGAAGTACTCGTTGGCAACCGGCAACTGGGGCGATCAGAAGAAGGCCATGAGCTCCACCGCCGGTGTCTCGCAAGTCTTGAACCGGTACACATTTGCCTCCACACTTTCCCACTTGCGGCGAACCAACACGCCAATCGGTCGAGATGGCAAGCTCGCGAAACCTCGTCAACTCCATAACACGCATTGGGGCCTGGTCTGTCCTGCGGAGACGCCAGAGGGTCAGGCTTGTGGTCTGGTCAAGAACCTCTCCCTCATGTGCTACATCAGCGTTGGCACCAACGCTGATCCAATCGTCGACTTCATGATTGCTAGGAACATGGAAGTCCTCGAAGAGTACGAGCCCTTGCGGTATCCTAATGCCACCAAGGTCTTTGTCAACGGCACGTGGGTTGGCGTCCACCAGGATCCGAAGCATTTGGTCACGCTGGTGCAGAATCTACGGAGATCGAACATCATCTCATTCGAGGTTTCGCTTGTTAGAGACATCCGAGACCGAGAATTCAAGATTTTCTCTGACGCTGGCCGCGTCATGAGACCACTCTTCGTGGTTGAACAGGAAGACGACCACGAGGGCAAGACTAGCAAGGTGGAGAAGGGCCAGCTCGTTCTTACAAAGGCACACATCCATCAATTAGAACGAGACAAGGAGGTTGGGAGATACCACAAGGACTACTTTGGGTGGAATGGCCTTCTCAGGTCCGGGTGCATCGAATACCTCGATGCCGAAGAGGAAGAGACCACCATGATCTGCATGACGCCAGAGGACTTGGACATTTACCGGCTGACTAAGCT------------CGGCTTCACCGTCAACGAGGAGGATCACAGCGAGGGCAACAAGCGCATCAAAACTAGACTGAACCCGACTACCCACATGTACACCCATTGCGAGATCCACCCCAGCATGCTGCTCGGCATCTGTGCCAGCATCATCCCCTTTCCGGATCACAACCAGGCTTGTTGACCAAGTTCTCGATGTCGTCCGTCGCGAGGCCGAGGGCTGCGACTGCCTCCAGGGCTTCCAGATCACCCACTCTCTCGGCGGTGGTACCGGTGCCGGTATGGGTACCCTCCTGATCTCCAAAATTCGCGAGGAGTTCCCCGACCGCATGATGGCCACTTTCTCGGTCGTCCCCTCCCCCAAGGTGTCGGATACCGTCGTTGAGCCGTACAACGCCACCCTTTCTGTCCACCAGCTTGTCGAGAACTCGGACGAGACCTTTTGCATTGACAACGAGGCTCTCTACGACATCTGCATGCGCACGCTCAAGCTGTCGAACCCCTCGTACGGCGACCTCAACCACCTGGTCTCTGCCGTCATGTCGGGTGTCACTGTTTCCCTGCGCTTCCCCGGCCAGCTCAACTCCGACCTTCGCAAGCTTGCCGTCAATATGGTTCCTTTCCCGCGTCTCCATTTCTTCATGGTCGGCTTCGCGCCTCTTACGAGCCGTGGCGCGCACTCTTTCCGTGCCGTCTCCGTCCCTGAGTTGACTCAGCAGATGTTCGACCCCAAGAACATGATGGCTGCTTCCGATTTCCGCAACGGTCGCTACCTGACTTGCTCTGCCATCTT

>*Immersiella*_*immersa*_SMH2589

???????????????????????????????????????????????????????????????????????????????????????????????????????????????????????????????????????????????????????????????????????????????????????????????????????????????????????????????????????????????????????????????????????????????????????????????????????????????????????????????????????????????????????????????????????????????????????????????????????????????????????????????????????????????????????????????????????????????????????????????????????????????????????????????????????????????????????????????????????????????????????????????????????????????????????????????????????????????????????AAAGAAACCAACAGGG-ATTGCCCCAGTAACGGCGAGTGAAGCGGCAACAGCTCAAATTTGAAATCTGGCTTCGG-CCCGAGTTGTAATTTGCAGAGGAAGATTCTGGTGACGCGCCGTCCGAGTCCCCTGGAACGGGGCGCCATAGAGGGTGAGAGCCCCGTATGGATGGATGCCTAGCCTGTGTGAATCTCCTTCGACGAGTCGAGTAGTTTGGGAATGCTGCTCAAAATGGGAGGTAAATTCCTTCTAAAGCTAAATATTGGCCAGAGACCGATAGCGCACAAGTAGAGTGATC-------GAAAGATGAAAAGCACTTTGAAAAGAGGGTTAAACAGCACGTGAAATTGTTGAAAGGGAAGCGCTTGTGACCAGACTTGCGCCTGGCTGATCATCCGGTGTTCTCACCGGTGCACTCTGCCAGGCTCAGGCCAGCATCGGTTTTCGTGGGGGGATAAAGGTCCTGGGAACGTAGCTCCTCTGGGAGTGTTATAGCCCGGGGCGTAATGCCCTCGCGGGGACCGAGGTTCGCGCATCTGCAAGGATGCTGGCGTAATGGTCATCAGCGACCCGTCTTGAAACACGGACCAAGGAGTCAAGGTTTTGCGCGAGTGTTTGGGTGTCAAACCCGCACGCGTAATGAAAGTGAA-CGTAGGTGAGAG----CTTCGGCGCATCATCGACCGATCCTGATGTATTCGGATGGATTTGAGTAGGAGCGTTAAGCCTTGGACCCGAAAGATGGTGAACTATGCTTGGATAGGGTGAAGCCAGAGGAAACTCTGGTGGAGGCTCGCAGCGGTTCTGACGTGCAAATCGATCGTCAAATC-TGAGCATGGGGGCGAAAGACTAATCGAACCATCTAGTAGCTGGTTACCGCC????????????????????????????????????????????????????????????????????????????????????????????????????????????????????????????????????????????????????????????????????????????????????????????????????????????????????????????????????????????????????????????????????????????????????????????????????????????????????????????????????????????????????????????????????????????????????????????????????????????????????????????????????????????????????????????????????????????????????????????????????????????????????????????????????????????????????????????????????????????????????????????????????????????????????????????????????????????????????????????????????????????????????????????????????????????????????????????????????????????????????????????????????????????????????????????????????????????????????????????????????????????????????????????????????????????????????????????????????????????????????????????????????????????????????????????????????????????????????????????????????????????????????????????????????????????????????????????????????????????????????????????????????????????????????????????????????????????????????????????????????????????????????????????????????????????????????????????????????????????????????????????????????????????????????????????????????????????????????????????????????????????????????????????????????????????????????????????????????????????????????????????????????????????????????????????????????????????????????????????????????????????????????????????????????????????????????????????????????????????????????????????????????????????????????????????????????????????????

>*Immersiella*_*immersa*_SMH4104

???????????????????????????????????????????????????????????????????????????????????????????????????????????????????????????????????????????????????????????????????????????????????????????????????????????????????????????????????????????????????????????????????????????????????????????????????????????????????????????????????????????????????????????????????????????????????????????????????????????????????????????????????????????????????????????????????????????????????????????????????????????????????????????????????????????????????????????????????????????????????????????????????????????????????????????????????????????????????????AAAGAAACCAACAGGG-ATTGCCCCAGTAACGGCGAGTGAAGCGGCAACAGCTCAAATTTGAAATCTGGCTTCGG-CCCGAGTTGTAATTTGCAGAGGAAGATTCTGGTGACGCGCCGTCCGAGTCCCCTGGAACGGGGCGCCATAGAGGGTGAGAGCCCCGTATGGATGGATGCCTAGCCTGTGTGAATCTCCTTCGACGAGTCGAGTAGTTTGGGAATGCTGCTCAAAATGGGAGGTAAATTCCTTCTAAAGCTAAATATTGGCCAGAGACCGATAGCGCACAAGTAGAGTGATC-------GAAAGATGAAAAGCACTTTGAAAAGAGGGTTAAACAGCACGTGAAATTGTTGAAAGGGAAGCGCTTGTGACCAGACTTGCGCCTGGCTGATCATCCGGTGTTCTCACCGGTGCACTCTGCCAGGCTCAGGCCAGCATCGGTTTTCGTGGGGGGATAAAGGTCCTGGGAACGTAGCTCCTCTGGGAGTGTTATAGCCCGGGGCGTAATGCCCTCGCGGGGACCGAGGTTCGCGCATCTGCAAGGATGCTGGCGTAATGGTCATCAGCGACCCGTCTTGAAACACGGACCAAGGAGTCAAGGTTTTGCGCGAGTGTTTGGGTGTCAAACCCGCACGCGTAATGAAAGTGAA-CGTAGGTGAGAG----CTTCGGCGCATCATCGACCGATCCTGATGTATTCGGATGGATTTGAGTAGGAGCGTTAAGCCTTGGACCCGAAAGATGGTGAACTATGCTTGGATAGGGTGAAGCCAGAGGAAACTCTGGTGGAGGCTCGCAGCGGTTCTGACGTGCAAATCGATCGTCAAATC-TGAGCATGGGGGCGAAAGACTAATCGAACCATCTAGTAGCTGGTTACCGCCGCTCTAAAGTACTCGTTGGCAACCGGTAACTGGGGCGATCAGAAGAAGGCCATGAGTTCCACCGCCGGTGTCTCGCAAGTCTTGAACCGGTACACATTTGCCTCCACACTTTCCCATTTGCGGCGAACCAACACACCGATCGGTCGAGATGGCAAGCTTGCGAAACCTCGTCAACTCCATAACACACATTGGGGCCTGGTCTGCCCTGCAGAGACGCCAGAGGGTCAGGCCTGTGGCCTGGTCAAGAACCTCTCCCTCATGTGCTACATCAGCGTTGGCACCAACGCTGATCCAATCGTCGACTTCATGATTGCTAGGAACATGGAAGTCCTCGAAGAGTATGAGCCCTTGCGATATCCCAATGCCACCAAGGTCTTTGTCAACGGCACATGGGTTGGCGTCCACCAGGATCCGAAGCATTTGGTCACGCTGGTGCAGAATCTACGGAGATCGAACATCATCTCATTTGAGGTTTCGCTTGTTAGAGACATCCGGGACCGAGAATTCAAGATTTTCTCTGATGCTGGCCGCGTCATGAGACCGCTCTTTGTGGTTGAGCAGGAAGACGACCATGAGGGCAAGACTAGCAAGGTCGATAAGGGCCAGCTCGTTCTCACGAAGGCACACATCCATCAGCTGGAACGAGACAAGGAGGTTGGGAGATACCACAAGGACTACTTTGGGTGGAATGGCCTTCTCAGGTCCGGGTGCATCGAATACCTCGATGCCGAAGAGGAAGAGACTACCATGATCTGTATGACGCCGGAGGACTTGGACATTTACCGGCTGAACAAGCT------------TGGCTTCGGCGTCCACGAGGAGGATCACAGCGAGGGCAACAAGCGCATCAAAACGAGACTGAACCCGACTACTCACATGTACACCCATTGCGAGATCCACCCCAGCATCCTGCTTGGCATCTGTGCCAGCATCATCCCCTTCCCGGATCACAACCAGGCTTGTTGACCAAGTTCTCGATGTCGTCCGTCGCGAGGCCGAGGGCTGCGACTGCCTTCAGGGTTTCCAGATCACCCACTCTCTCGGTGGTGGTACCGGTGCCGGTATGGGTACCCTCCTGATCTCCAAGATTCGCGAAGAGTTCCCCGACCGCATGATGGCCACTTTCTCGGTCGTCCCCTCCCCCAAGGTGTCGGATACCGTCGTTGAGCCGTACAACGCCACCCTTTCCGTCCACCAGCTTGTCGAGAACTCGGACGAGACCTTCTGCATTGACAACGAGGCTCTCTACGACATCTGCATGCGTACGCTCAAGCTGTCGAACCCCTCGTACGGCGATCTCAACCACCTAGTCTCTGCCGTCATGTCGGGTGTCACTGTTTCCCTGCGCTTCCCCGGCCAGCTCAACTCCGACCTTCGCAAGCTTGCCGTCAACATGGTTCCTTTCCCGCGCCTCCATTTCTTCATGGTCGGCTTCGCGCCTCTTACGAGCCGTGGCGCGCATTCTTTCCGTGCCGTCTCCGTCCCTGAGTTGACCCAGCAGATGTTTGACCCCAAGAACATGATGGCTGCCTCCGATTTCCGCAACGGTCGCTACCTAACTTGCTCTGCCATCTT

>*Jugulospora_rotula*_ATCC_38359

???????????????????????????????????????????????????????????????????????????????????????????????????????????????????????????????????????????????????????????????????????????????????????????????????????????????????????????????????????????????????????????????????????????????????????????????????????????????????????????????????????????????????????????????????????????????????????????????????????????????????????????????????????????????????????????????????????????????????????????????????????????????????????????????????????????????????????????????????????????????????????????????????????????????????????????????????????????????????????AAAGAAACCAACAGGG-ATTGCCCCAGTAACGGCGAGTGAAGCGGCAACAGCTCAAATTTGAAATCTGGCCTCGG-CCCGAGTTGTAATTTGTAGAGGAAGCTTCTGGTGCGGTCTGTTCCGAGTCCCCTGGAACGGGGCGCCATAGAGGGTGAGAGCCCCGTACGGACGGATACCAATCCTGTGTGAAGCTCCTTCGACGAGTCGAGTAGTTTGGGAATGCTGCTCAAAATGGGAGGTAAATTCCTTCTAAAGCTAAATACCGGCCAGAGACCGATAGCGCACAAGTAGAGTGATC-------GAAAGATGAAAAGCACTTTGAAAAGAGGGTTAAACAGCACGTGAAATTGTTGAAAGGGAAGCGCTTGTGACCAGACTTGCGCCAGGCTGATCATCCGGTGTTCTCACCGGTGCACTCTGCCTGGCTCAGGCCAGCATCGGTTTCCGCGGGGGGATAAAGGTCCCGGGAATGTAGCTCCTCCGGGAGTGTTATAGCCCGGGGCGCAATGCCCTCGTGGGGACCGAGGTTCGCGCATCTGCAAGGATGCTGGCGTAATGGTCATCAGCGACCCGTCTTGAAACACGGACCAAGGAGTCAAGGTTTTGCGCGAGTGTTTGGGTGTTAAACCCGCACGCGTAATGAAAGTGAA-CGTAGGTGAGAG----CTTCGGCGCATCATCGACCGATCCTGATGTATTCGGATGGATTTGAGTAGGAGCGTTAAGCCTTGGACCCGAAAGATGGTGAACTATGCTTGGATAGGGTGAAGCCAGAGGAAACTCTGGTGGAGGCTCGCAGCGGTTCTGACGTGCAAATCGATCGTCAAATC-TGAGCATGGGGGCGAAAGACTAATCGAACCATCTAGTAGCTGGTTACCGCCGCGCTCAAATACTCGCTGGCGACTGGAAACTGGGGTGATCAAAAGAAGGCCATGAGCTCCACCGCGGGTGTCTCGCAGGTCCTGAATCGATACACATTTGCCTCTACGCTTTCTCACTTGCGGCGAACGAACACACCCATTGGGCGCGATGGCAAGCTCGCAAAACCCCGCCAACTCCATAACACACACTGGGGTCTGGTCTGTCCAGCAGAGACGCCCGAAGGCCAGGCTTGCGGTCTGGTCAAGAATCTCTCGCTGATGTGCTACATTAGCGTTGGTACCAATGCGGATCCCATCGTCGAATTTATGATTGCCAGGAACATGGAAGTCCTTGAAGAGTATGAGCCTCTGCGGTATCCCAATGCCACAAAGGTTTTCGTCAACGGCACCTGGGTCGGTGTCCACCAAGACCCAAAGCACCTGGTCAGCTTGGTTCAGAGCCTAAGGAGATCCAACATCATCAGTTTCGAGGTTTCCCTGGTTCGTGACATCCGAGACAGAGAGTTCAAGATCTTCTCCGATGCAGGGCGTGTCATGAGACCGCTATTCGTCGTTGAACAGGAGGACGACGGCGAG---------AGCAAGGTTGAGAAGGGCCAGCTGGTTCTTACAAAGTCGCAAGTCCTCAAACTAGAAAAAGACAAGGAGATTGGCAAATACCATCCGGATTACTTCGGCTGGAATGGCCTCTTGAGGGAGGGCTGTGTCGAATACCTTGATGCGGAAGAAGAGGAAACGGCGATGATCTGTATGACGCCCGAGGACCTCGACACGTACCGGCTGGCCAAGCT------------TGGGTTCAACGTCGCCGAGGAAGATCCTAGTGAAGGCAACAAGCGCATCAAGACTAGGCTTAACCCGACGACCCATATGTATACTCACTGCGAGATACACCCCAGCATGCTGCTTGGTATCTGCGCCAGCATCATTCCCTTCCCCGATCACAACCAGGCTTGTTGACCAGGTTCTCGATGTCGTCCGTCGCGAGGCCGAGGGCTGCGACTGCCTCCAGGGCTTCCAGATCACCCACTCTCTCGGTGGTGGTACCGGTGCCGGTATGGGTACCCTCCTGATCTCCAAGATTCGCGAGGAGTTCCCCGACCGCATGATGGCGACCTTCTCTGTCGTCCCCTCCCCCAAGGTCTCTGATACCGTTGTGGAGCCCTACAACGCCACGCTCTCCGTCCATCAGCTTGTCGAGAACTCCGACGAGACCTTCTGCATTGACAACGAGGCTCTCTACGACATCTGCATGCGGACGCTCAAGCTGTCCAACCCTTCATACGGTGATCTCAACCACCTGGTCTCTGCCGTCATGTCCGGCGTTACCGTTTCCCTGCGCTTCCCCGGCCAGCTCAACTCAGATCTCCGCAAGCTCGCCGTGAACATGGTTCCCTTCCCCCGTCTCCACTTCTTCATGGTCGGCTTCGCGCCGCTTACTAGCCGTGGCGCGCACTCTTTCCGTGCTGTCTCGGTTCCTGAGTTGACCCAGCAGATGTTCGACCCCAAGAACATGATGGCTGCGTCTGACTTCCGCAACGGTCGCTACCTGACTTGCTCTGCCATCTT

>*Jugulospora*_*antarctica*_IMI_381338

???????????????????????????????????????????????????????????????????????????????????????????????????????????????????????????????????????????????????????????????????????????????????????????????????????????????????????????????????????????????????????????????????????????????????????????????????????????????????????????????????????????????????????????????????????????????????????????????????????????????????????????????????????????????????????????????????????????????????????????????????????????????????????????????????????????????????????????????????????????????????????????????????????????????????????????????????????????????????????AAAGAAACCAACAGGG-ATTGCCCCAGTAACGGCGAGTGAAGCGGCAACAGCTCAAATTTGAAATCTGGCCTCGG-CCCGAGTTGTAATTTGCAGAGGAAGCTTCTGGTGCGGTCCGTCCCGAGTCCCCTGGAACGGGGCGCCGTAGAGGGTGAGAGCCCCGTACGGACGGATACCAATCCTGTGTGAAGCTCCTTCGACGAGTCGAGTAGTTTGGGAATGCTGCTCAAAATGGGAGGTAAATTCCTTCTAAAGCTAAATACCGGCCAGAGACCGATAGCGCACAAGTAGAGTGATC-------GAAAGATGAAAAGCACTTTGAAAAGAGGGTTAAACAGCACGTGAAATTGTTGAAAGGGAAGCGCTTGTGACCAGACTTGCGCCAGGCTGATCATCCGGTGTTCTCACCGGTGCACTCGGCCTGGCTCAGGCCAGCATCGGTTCCCGCGGGGGGATAAAGGTCCCGGGAATGTAGCTCCTCCGGGAGTGTTATAGCCCGGGGCGCAATGCCCCCGTGGGGACCGAGGTTCGCGCATCTGCAAGGATGCTGGCGTAATGGTCATCAGCGACCCGTCTTGAAACACGGACCAAGGAGTCAAGGTTTTGCGCGAGTGTTTGGGTGTCAAACCCGCACGCGTAATGAAAGTGAA-CGTAGGTGAGAG----CTTCGGCGCATCATCGACCGATCCTGATGTCTTCGGATGGATTTGAGTAGGAGCGTTAAGCCTTGGACCCGAAAGATGGTGAACTATGCTTGGATAGGGTGAAGCCAGAGGAAACTCTGGTGGAGGCTCGCAGCGGTTCTGACGTGCAAATCGATCGTCAAATC-TGAGCATGGGGCGAA????????????????????????????????????GCGCTCAAGTACTCGCTGGCGACTGGAAACTGGGGTGATCAAAAGAAAGCCATGAGCTCCACTGCGGGTGTCTCGCAGGTCCTGAACCGATACACATTTGCCTCTACACTTTCTCACTTGCGGCGAACGAACACGCCTATTGGGCGCGATGGCAAGCTCGCAAAACCCCGCCAACTCCATAACACACACTGGGGTCTGGTCTGTCCAGCAGAGACGCCCGAAGGCCAGGCTTGCGGTCTGGTCAAGAATCTCTCGCTGATGTGCTACATTAGCGTTGGTACCAATGCGGATCCTATCATCGAATTCATGATTGCCAGGAACATGGAAGTCCTGGAAGAGTATGAGCCTCTGCGGTATCCGAATGCCACAAAGGTTTTCGTCAATGGCACCTGGGTCGGTGTCCACCAAGACCCAAAGCACCTGGTCAGCTTGGTTCAGAGCCTAAGGAGATCCAACATTATCAGTTTCGAGGTTTCCCTGGTTCGTGACATCCGAGATAGGGAGTTCAAGATCTTCTCCGATGCAGGGCGTGTCATGAGACCGCTATTCGTCGTTGAACAGGAGGATGACGGCGAG---------AGCAAGGTTGAGAAGGGCCAGCTGGTTCTTACAAAGTCGCAAATCCTCAAACTAGAAAAAGACAAGGAGATTGGCAAATACCATCCGGATTACTTCGGCTGGAATGGCCTCCTAAGGGAGGGCTGTGTCGAATACTTGGATGCGGAAGAAGAGGAAACGGCGATGATCTGTATGACGCCCGAGGACCTCGACACGTACCGGCTGGCCAAGCT------------TGGGTTCAACGTCGCCGAGGAGGATCCTAGTGAAGGCAACAAGCGCATCAAGACTAGGCTCAACCCGACGACCCATATGTATACTCACTGCGAGATACACCCCAGCATGCTGCTTGGTATCTGCGCCAGCATCATTCCCTTCCCCGATCACAAC???GCTTGTTGACCAAGTTCTCGATGTCGTCCGTCGCGAGGCCGAGGGCTGCGACTGCCTCCAGGGCTTCCAGATCACCCACTCTCTCGGTGGTGGTACCGGTGCCGGTATGGGTACCCTCTTGATCTCCAAGATTCGCGAGGAGTTCCCCGACCGCATGATGGCGACTTTCTCTGTCGTCCCCTCCCCCAAGGTCTCTGACACCGTCGTTGAGCCCTACAACGCCACGCTCTCCGTCCACCAACTTGTCGAGAACTCGGACGAGACCTTCTGCATTGACAACGAGGCTCTCTACGACATCTGCATGCGGACGCTCAAGCTGTCCAACCCTTCATACGGTGATCTCAACCACCTGGTCTCTGCCGTCATGTCCGGCGTTACCGTTTCCCTGCGCTTCCCCGGCCAGCTCAACTCCGATCTCCGCAAGCTCGCCGTGAACATGGTTCCTTTCCCCCGTCTCCACTTCTTCATGGTCGGCTTCGCGCCGCTTACTAGCCGTGGAGCGCACTCTTTCCGTGCTGTCTCGGTTCCCGAGTTGACCCAGCAGATGTTCGACCCCAAGAACATGATGGCTGCGTCTGACTTCCGCAACGGTCGCTACCTGACCTGCTCTGCCATCTT

>*Zygopleurage*_*zygospora*_SMH4219

???????????????????????????????????????????????????????????????????????????????????????????????????????????????????????????????????????????????????????????????????????????????????????????????????????????????????????????????????????????????????????????????????????????????????????????????????????????????????????????????????????????????????????????????????????????????????????????????????????????????????????????????????????????????????????????????????????????????????????????????????????????????????????????????????????????????????????????????????????????????????????????????????????????????????????????????????????????????????????AAAGAAACCAACCGGG-ATTGCCCTAGTAACGGCGAGTGAAGCGGCAACAGCTCAAATTTGAAATCTGGCTCCGG-CCCGAGTTGTAATTTGCAGAGGAAGCTTCTGGCGAAACACCTTCTAAGTCCCCTGGAACGGGGCGCCACAGTGGGTGAGAGCCCCATGT-GATGGATGTTGAACCTGTGTGAAGCTCCTTCGACGAGTCGAGTAGTTTGGGAATGCTGCTCAAAATGGGAGGTAAATTCCTTCTAAAGCTAAATATTGGCCAGAGACCGATAGCGCACAAGTAGAGTGATC-------GAAAGATGAAAAGCACTTTGAAAAGAGGGTTAAACAGCACGTGAAATTGTTGAAAGGGAAGCGCTTGTGACCAGACTTGCGCTGGGCTGATCATCCGGTGTTCTCACCGGTGCACTCGGCCCAGCTCAGGCCAGCATCGGTTTCGGCGGGGGGATAAAGGCGCCAGGAACGTAGCTCCCCCGGGAGTGTTATAGCCTGGCGTGCAATGCCCCCGCTGGGACCGAGGTTCGCGCATCTGCAAGGATGCTGGCGTAATGGTCATCAGCGACCCGTCTTGAAACACGGACCAAGGAGTCAAGGTTTTGCGCGAGTGTTTGGGTGTCAAACCCGCACGCGTAATGAAAGTGAA-CGTAGGTGAGAG----CTTCGGCGCATCATCGACCGATCCTGATGTATTCGGATGGATTTGAGTAGGAGCGTTAAGCCTTGGACCCGAAAGATGGTGAACTATGCTTGGATAGGGTGAAGCCAGAGGAAACTCTGGTGGAGGCTCGCAGCGGTTCTGACGTGCAAATCGATCGTCAAATC-TGAGCATGGGGGCGAAAGACTAATCGAACCATCTAGTAGCTGGTTACCGCC??????????????????????????????????????????????????????????????????????????????????????????????????????????????????????????????????????????????????????????????????????????????????????????????????????????????????????????????????????????????????????????????????????????????????????????????????????????????????????????????????????????????????????????????????????????????????????????????????????????????????????????????????????????????????????????????????????????????????????????????????????????????????????????????????????????????????????????????????????????????????????????????????????????????????????????????????????????????????????????????????????????????????????????????????????????????????????????????????????????????????????????????????????????????????????????????????????????????????????????????????????????????????????????????????????????????????????????????????????????????????????????????????????????????????????????????????????????????????????????????????????GCTTGTTGACCAAGTTCTCGATGTCGTCCGTCGTGAGGCTGAGGGCTGCGACTGCCTCCAGGGCTTCCAGATCACCCACTCCCTCGGTGGTGGTACCGGTGCCGGTATGGGTACCCTCCTTATCTCCAAGATTCGCGAGGAGTTCCCCGACCGCATGATGGCGACCTTCTCCGTCGTTCCCTCCCCTAAGGTCTCGGATACCGTCGTCGAGCCCTACAACGCCACCCTGTCGGTGCACCAGCTTGTCGAGAACTCGGATGAGACCTTCTGCATTGACAACGAGGCTCTCTACGACATCTGCATGAGGACGCTCAAGCTCTCCAACCCCTCTTATGGCGACCTTAACCACCTCGTCTCCGCTGTCATGTCGGGTGTCACCGTTTCGCTCCGTTTCCCTGGCCAGCTTAACTCCGACCTGCGCAAGCTTGCTGTCAACATGGTYCCCTTCCCTCGTCTGCACTTCTTCATGGTCGGCTTCGCGCCTCTTACTAGCCGTGGCGCGCACTCTTTCCGTGCTGTCTCGGTTCCTGAGCTCACCCAGCAGATGTTCGACCCCAAGAACATGATGGCCGCCTCCGACTTCCGCAACGGTCGCTACCTCACTTGCTCTGCCATCTT

>*Zopfiella*_*tardifaciens*_CBS_670_82

???????????????????????????????????????????????????????????????????????????????????????????????????????????????????????????????????????????????????????????????????????????????????????????????????????????????????????????????????????????????????????????????????????????????????????????????????????????????????????????????????????????????????????????????????????????????????????????????????????????????????????????????????????????????????????????????????????????????????????????????????????????????????????????????????????????????????????????????????????????????????????????????????????????????????????????????????????????????????????AAAGAAACCAACAGGG-ATTGCCCTAGTAACGGCGAGTGAAGCGGCAACAGCTCAAATTTGAAATCTGGCTCCGG-CCCGAGTTGTAATTTGCAGAGGAAGCTTCTGGTGATATACTGTCTAAGTCCCCTGGAACGGGGCGCCACAGTGGGTGAGAGCCCCATAT-GACAGCTGTAGATCCTGTGTGAAGCTCCTTCGACGAGTCGAGTAGTTTGGGAATGCTGCTCAAAATGGGAGGTAAATTCCTTCTAAAGCTAAATATTGGCCAGAGACCGATAGCGCACAAGTAGAGTGATC-------GAAAGATGAAAAGCACTTTGAAAAGAGAGTTAAACAGCACGTGAAATTGTTGAAAGGGAAGCGCTTGTGACCAGACTTGCGCTGGGCTGATCATCCGGTGTTCTCACCGGTGCACTCGGCCCAGCTCAGGCCAGCATCGGTTTTGGTGGGGGGATAAAGGCGCTGGGAACGTAGCTCCCCCGGGAGTGTTATAGCCCAGCGTGCAATACCCCCGCTGGGACCGAGGTTCGCGCATCTGCAAGGATGCTGGCGTAATGGTCATCAGCGACCCGTCTTGAAACACGGACCAAGGAGTCAAGGTTTTGCGCGAGTGTTTGGGTGTCAAACCCGCACGCGTAATGAAAGTGAA-CGTAGGTGAGAG----CTTCGGCGCATCATCGACCGATCCTGATGTATTCGGATGGATTTGAGTAGGAGCGTTAAGCCTTGGACCCGAAAGATGGTGAACTATGCTTGGATAGGGTGAAGCCAGAGGAAACTCTGGTGGAGGCTCGCAGCGGTTCTGACGTGCAAATCGATCGTCAAATC-TGAGCATGGGGGCGAAAGACTAATCGA??????????????????????????????????????????????????????????????????????????????????????????????????????????????????????????????????????????CATTTGCGGCGAACCAACACGCCTATCGGTCGCGACGGCAAGCTTGCCAAACCCCGACAGCTGCACAATACGCACTGGGGCCTCGTGTGCCCTGCCGAGACTCCAGAAGGCCAGGCTTGCGGCCTCGTCAAGAACCTCTCCCTGATGTGCTATATTAGCGTGGGCACTAATGCTGAACCCATCGTCGACTTCATGGTTGCTAGAAACATGGAAGTCCTGGAAGAGTATGAGCCCCTCCGCTATCCCAACGCCACCAAGGTCTTCGTCAACGGAACCTGGGTCGGCGTGCACCAAGACCCGAAGCACTTGGTCACCCTAGTCCAGAATCTCAGGCGGTCCAACGTCATCTCCTTTGAAGTTTCCTTGGTGCGGGATATCCGAGACCGAGAGTTCAAGATCTTCTCTGATGCTGGCCGCGTCATGAGACCGCTTTTTGTTGTCGAGCAGGAGGACGAGAACAAG------CACACCAAGGTCCAAAAGGGCCAGCTAGTCCTAACTAGGGAACACATCAACCGGCTGGATCGGGACAAGGACCTCACGCAACTCGACGAGGACTTCTTTGGCTGGAACGGCCTCCTGAGGGAAGGCTGTGTCGAGTACCTGGACGCCGAGGAAGAGGAGACGGCCATGATCTGCATGACCCCCGAAGACCTCGACCACTACCGGTCTACCAAGTTGGGC---ATTAAACCAAAAAAGAAAACAGACGAGGAGGAGGAGGGGCACAACCAGCGCATCAAAACGAAGGCGAACCCGACCACCCACATGTATACTCATTGCGAGATTCATCCTAGTATGTTGCTCGGCATCTGCGCGAGCATCATCCCCTTCCCAGATCACAACCAG??????????????????????????????????????????????????????????????????????????????????????????????????????????????????????????????????????????????????????????????????????????????????????????????????????????????????????????????????????????????????????????????????????????????????????????????????????????????????????????????????????????????????????????????????????????????????????????????????????????????????????????????????????????????????????????????????????????????????????????????????????????????????????????????????????????????????????????????????????????????????????????????????????????????????????????????????????????

>*Jugulospora*_*carbonaria*_ATCC_34567

???????????????????????????????????????????????????????????????????????????????????????????????????????????????????????????????????????????????????????????????????????????????????????????????????????????????????????????????????????????????????????????????????????????????????????????????????????????????????????????????????????????????????????????????????????????????????????????????????????????????????????????????????????????????????????????????????????????????????????????????????????????????????????????????????????????????????????????????????????????????????????????????????????????????????????????????????????????????????????AAAGAAACCAACAGGG-ATTGCCCCAGTAACGGCGAGTGAAGCGGCAACAGCTCAAATTTGAAATCTGGCCTCGG-CCCGAGTTGTAATTTGTAGAGGAAGCTTCTGGTGCGGTCCGTCCCGAGTCCCCTGGAACGGGGCGCCGAAGAGGGTGAGAGCCCCGTACGGACGGATACCAATCCTGTGTGAAGCTCCTTCGACGAGTCGAGTAGTTTGGGAATGCTGCTCAAAATGGGAGGTAAATTCCTTCTAAAGCTAAATACCGGCCAGAGACCGATAGCGCACAAGTAGAGTGATC-------GAAAGATGAAAAGCACTTTGAAAAGAGGGTTAAACAGCACGTGAAATTGTTGAAAGGGAAGCGCTTGTGACCAGACTTGCGCCAGGCTGATCATCCGGTGTTCTCACCGGTGCACTCGGCCTGGCTCAGGCCAGCATCGGTTCCCGCGGGGGGATAAAGGTCCCGGGAATGTAGCTCCTCCGGGAGTGTTATAGCCCGGGGCGCAATGCCCCCGTGGGGACCGAGGTTCGCGCATCTGCAAGGATGCTGGCGTAATGGTCATCAGCGACCCGTCTTGAAACACGGACCAAGGAGTCAAGGTTTTGCGCGAGTGTTTGGGTGTCAAACCCGCACGCGTAATGAAAGTGAA-CGTAGGTGAGAG----CTTCGGCGCATCATCGACCGATCCTGATGTCTTCGGATGGATTTGAGTAGGAGCGTTAAGCCTTGGACCCGAAAGATGGTGAACTATGCTTGGATAGGGTGAAGCCAGAGGAAACTCTGGTGGAGGCTCGCAGCGGTTCTGACGTGCAAATCGATCGTCAAATC-TGAGCATGGGGGCGAAAGACTAATCGAACCATCTAGTAGCTGGTTACCGCCGCGCTCAAGTACTCGCTGGCGACTGGAAACTGGGGTGATCAAAAGAAAGCTATGAGCTCCACTGCGGGTGTCTCGCAGGTCCTGAACCGATACACATTTGCCTCTACACTTTCTCACTTGCGGCGAACGAACACGCCTATTGGGCGCGATGGCAAGCTCGCAAAACCCCGCCAACTCCATAACACACACTGGGGTCTGGTCTGTCCAGCAGAGACGCCCGAAGGCCAGGCTTGCGGTCTGGTCAAGAATCTCTCGCTGATGTGCTACATTAGCGTTGGTACCAATGCGGATCCTATCATCGAATTCATGATTGCCAGGAACATGGAAGTCCTGGAAGAGTATGAGCCTCTGCGGTATCCGAATGCCACAAAGGTTTTCGTCAATGGCACCTGGGTCGGTGTCCACCAAGACCCAAAGCACCTGGTCAGCTTGGTTCAGAGCCTAAGGAGATCCAATATTATCAGTTTCGAGGTTTCCCTGGTTCGCGACATCCGAGACAGAGAGTTCAAGATCTTCTCCGATGCAGGGCGTGTCATGAGACCGCTATTCGTCGTTGAACAGGAGGATGACGGTGAG---------AGCAAGGTTGAGAAGGGCCAGCTGGTTCTTACCAAGTCGCAAATCCTCAAACTAGAAAAAGACAAGGAGATTGGCAAATACCATCCGGATTACTTCGGCTGGAATGGCCTCTTGAGGGAAGGCTGTGTCGAATACCTGGATGCGGAAGAAGAGGAAACGGCGATGATCTGTATGACGCCCGAGGACCTCGACACGTACCGGCTGGCCAAGCT------------TGGGTTCAACGTCGCTGAGGAGGATCCTAGTGAAGGCAACAAGCGCATCAAGACTAGGCTCAACCCGACGACCCATATGTATACTCACTGCGAGATACACCCCAGCATGCTGCTTGGTATCTGCGCCAGCATCATTCCCTTCCCAGATCACAACCAGGCTTGTTGACCAAGTTCTCGATGTCGTCCGTCGCGAGGCCGAGGGCTGCGACTGCCTCCAGGGCTTCCAGATCACTCACTCTCTCGGTGGTGGTACCGGTGCCGGTATGGGTACCCTCCTGATCTCCAAGATTCGCGAGGAGTTCCCCGACCGCATGATGGCGACCTTCTCTGTCGTCCCCTCCCCCAAGGTCTCCGACACCGTCGTTGAGCCCTACAACGCCACGCTCTCCGTCCACCAGCTTGTCGAGAACTCGGACGAGACCTTCTGCATTGACAACGAGGCTCTCTACGACATCTGCATGCGGACGCTCAAGCTGTCCAACCCTTCATACGGTGATCTCAACCACCTGGTCTCTGCCGTCATGTCCGGCGTTACCGTTTCCCTGCGCTTCCCCGGCCAGCTCAACTCCGATCTCCGCAAGCTCGCCGTGAACATGGTTCCTTTCCCCCGTCTCCACTTCTTCATGGTCGGCTTCGCGCCGCTTACCAGCCGTGGCGCGCACTCTTTCCGTGCTGTCTCGGTTCCTGAGTTGACCCAGCAGATGTTCGACCCCAAGAACATGATGGCTGCGTCTGACTTCCGCAACGGTCGCTACCTGACCTGCTCTGCCATCTT

>*Schizothecium*_*vesticola*_SMH3187

??????????????????????????????????????????????????????????????????????????????????????????????????????????????????????????????????????????????????????????????????????????????????????????????????????????????????????????????????????????????????????????????????????????????????????????????????????????????????????????????????????????????????????????????????????????????????????????????????????????????????????????????????????????????????????????????????????????????????????????????????????????????????????????????????????????????????????????????????????????????????????????????????????????????????????CCGCTGAACTTAAGCATATCAATAAGCGGAGGAAAAGAAACCAACAGGG-ATTGCCCCAGTAACGGCGAGTGAAGCGGCAACAGCTCAAATTTGAAATCTGGCCTCGG-CCCGAGTTGTAATTTGTAGAGGAAGCTTCTGGTGCGGCGCCGTCCGAGTCTCCTGGAACGGAGCGCCATAGAGGGTGAGAGCCCCGTATGGACGGATGCCAAACCTGTGTGAAGCTCCTTCGACGAGTCGAGTAGTTTGGGAATGCTGCTCTAAATGGGAGGTAAATTCCTTCTAAAGCTAAATATTGGCCAGAGACCGATAGCGCACAAGTAGAGTGATC-------GAAAGATGAAAAGCACTTTGAAAAGAGGGTTAAACAGCACGTGAAATTGTTGAAAGGGAAGCGCTTGTGACCAGACTTGAGCGCGGCGGATCATCCGGTGTTCTCACCGGTGCACTCCGCCGTGCCCAGGCCAGCATCGGTTTCCGCGGGGGGACAAAGGTCCCGGGAACGTAGCTCCTCCGGGAGTGTTATAGCCCGGGGCGTAATGCCCTCGTGGGGACCGAGGACCGCGCATCTGCAAGGATGCTGGCGTAATGGTCATCAGCGACCCGTCTTGAAACACGGACCAAGGAGTCAAGGTTTTGCGCGAGTGTTTGGGTGTTAAACCCGCACGCGTAATGAAAGTGAA-CGTAGGTGAGAG----CTTCGGCGCATCATCGACCGATCCTGATGTATTCGGATGGATTTGAGTAGGAGCGTTAAGCCTTGGACCCGAAAGATGGTGAACTATGCTTGGATAGGGTGAAGCCAGAGGAAACTCTGGTGGAGGCTCGCAGCGGTTCTGACGTGCAAATCGATCGTCAAATC-TGAGCATGGGGGCGAAAGACTAATCGAACCATCTAGTAGCTGGTTACCGCC????????????????????????????????????????????????????????????????????????????????????????????????????????????????????????????????????????????????????????????????????????????????????????????????????????????????????????????????????????????????????????????????????????????????????????????????????????????????????????????????????????????????????????????????????????????????????????????????????????????????????????????????????????????????????????????????????????????????????????????????????????????????????????????????????????????????????????????????????????????????????????????????????????????????????????????????????????????????????????????????????????????????????????????????????????????????????????????????????????????????????????????????????????????????????????????????????????????????????????????????????????????????????????????????????????????????????????????????????????????????????????????????????????????????????????????????????????????????????????????????????????????????????????????????????????????????????????????????????????????????????????????????????????????????????????????????????????????????????????????????????????????????????????????????????????????????????????????????????????????????????????????????????????????????????????????????????????????????????????????????????????????????????????????????????????????????????????????????????????????????????????????????????????????????????????????????????????????????????????????????????????????????????????????????????????????????????????????????????????????????????????????????????????????????????????????????????????????????

>*Apiosordaria*_*microcarpa*_CBS_692_82

ATTACAGAGTTGC---------AAAACTCC--CA-ACCCTTTGTGAACG-TACCTAA---------CAGTGCTTCGGCGG------------------AAGGCTCCTTAG----------------------------------------------------GGGGCGC-CCCGCCGAAGG-----AATAC-AAACTCT----TGATTTT-TTGGCATCTCTGAGC-AAC-TATAAAATAAGTTAAAACTTTC-AACAACGGATCTCTTGGTTCTGGCATCGATGAAGAACGCAGCGAAATGCGATAAGTAATGTGAATTGCAGAATTCAGTGAATCATCGAATCTTTGAACGCACATTGCGCCCGCTAGTATTCTGGCGGGCATGCCTGTTCGAGCGTCATTTCAA-CCATCAAGCCTCA-GGCTT-GTGTTGGAGCCCTGCGG----CTGCCCGCAGCCTCCTAAAAGCAGTGGCGGGCTCGCTAT-CACACCGAGTGCAGTAGTTTACTCTTCGCTCAGGGCGT-GTGGCGGGT-TCTAGCCGTAAAA---CCCCC--TACTTTTAAAGGTTGACCTCGGATCAGGTAGGAATACCCGCTGAACTTAAGCATATCAATAAGCGGAGGAAAAGAAACCAACAGGG-ATTGCCCTAGTAACGGCGAGTGAAGCGGCAACAGCTCAAATTTGAAATCTGGCTCCGG-CCCGAGTTGTAATTTGCAGAGGAAGCTTCTGGTGATATACCGTCTAAGTCCCCTGGAACGGGGCGCCACAGTGGGTGAGAGCCCCATAT-GACGGATGTAGATCCTGTGTGAAGCTCCTTCGACGAGTCGAGTAGTTTGGGAATGCTGCTCAAAATGGGAGGTAAATTCCTTCTAAAGCTAAATATTGGCCAGAGACCGATAGCGCACAAGTAGAGTGATC-------GAAAGATGAAAAGCACTTTGAAAAGAGGGTTAAACAGCACGTGAAATTGTTGAAAGGGAAGCGCTTATGACCAGACTTGCGCTGGGCTGATCATCCGGTGTTCTCACCGGTGCACTCGGCCCAGCTCAGGCCAGCATCGGTTTTGGTGGGGGGATAAAGGCGCTGGGAACGTAGCTCCTTCGGGAGTGTTATAGCCCAGCGTGCAATGCCCCCGCCGGGACCGAGGTTCGCGCATCTGCAAGGATG??????????????????????????????????????????????????????????????????????????????????????????????????????????????????????????????????????????????????????????????????????????????????????????????????????????????????????????????????????????????????????????????????????????????????????????????????????????????????????????????????????????????????????????????????????????????????????????????????????????????????????????????????????????TACACCTTTGCCTCGACGCTCTCCCATTTGCGGCGAACAAACACGCCCATCGGTCGCGATGGCAAGTTGGCCAAACCCCGGCAGCTGCACAATACACACTGGGGCCTTGTTTGCCCTGCCGAAACCCCAGAAGGCCAGGCTTGTGGCCTCGTCAAGAACCTCTCGTTAATGTGCTATATCAGCGTGGGCACGAATGCTGAGCCTATCATCGACTTTATGGTGGCCAGGAACATGGAAGTCCTTGAAGAGTACGAGCCGCTCCGCTACCCTAACGCCACCAAGGTCTTTGTCAACGGGACTTGGGTTGGTGTCCACCAAGACCCGAAGCACTTGGTCACATTGGTTCAGAATCTTAGGAGATCCAACGTTATCTCCTTCGAGGTTTCGCTTGTTCGCGACATCCGAGATCGAGAGTTCAAGATCTTCTCTGATGCTGGCCGAGTCATGAGACCGCTCTTCGTTGTTGAGCAGGAAGACGACAACAAG------TTGACCAAGGTCCAGAAGGGCCAGCTAGTCCTGACAAGAGAGCATATGAACCGGCTAGACCGGGACAAGGATATCGGACCAATGGACGAAGACTTCTTCGGCTGGAACGGCCTTCTGAGGGAGGGTTGTGTTGAGTACCTCGATGCCGAGGAAGAGGAGACAGCCATGATTTGCATGACCCCCGACGACCTGGAACACTATCGGTTCACCAGGATGGGGGC---AAAGACCAGACGGAGGAGCAGCCGGGAGACGATGGAGTATAACAAACGCATCACAACGAAGACGAGTCCGACCACGCAAATGTATACCCATTGCGAGATCCATCCCAGCATGTTACTCGGTATTTGCGCCAGCATTATCCCCTTCCCG??????????????????????????????????????????????????????????????????????????????????????????????????????????????????????????????????????????????????????????????????????????????????????????????????????????????????????????????????????????????????????????????????????????????????????????????????????????????????????????????????????????????????????????????????????????????????????????????????????????????????????????????????????????????????????????????????????????????????????????????????????????????????????????????????????????????????????????????????????????????????????????????????????????????????????????????????????????????????????

>*Jugulospora*_*vestita*

????????????????????????????????CA-CACCATCGTGAACGTCACCGC--------ATCGTTTCTTCGGCGG------------------GCGGCCCCCCACC------------GGGGCCGCGCCTG-------CCCCCTC-------GCGGGGCGGCAG-CCCGCCGGAGG-----CGTCC-AAACTCT--CAGCATCTA-GTGGCATCTCTGAGT-AGCTTACAAAATAAGTCAAAACTTTC-AACAACGGATCTCTTGGCTCTGGCATCGATGAAGAACGCAGCGAAATGCGATAAGTAATGCGAATTGCAGAATCCAGTGAGTCATCGAATCTTTGAACGCACATTGCGCCCGCCAGTATTCTGGCGGGCATGCCTGTCCGAGCGTCATTTCCA-CCATCAAGCCCTGCGGCTT-GTGTTGGGGCCCTGCGG----CCGCCCGCAGCCCCCGGAATGCAGTGGCGGGCTCGTTGT-CACCCCGAGTGCAGTAA--TGCTCTTCTCTCGCGGCGTGGCGGCGGGT-TCCGGCCGTGAAA---CCAACCAAACTCATCAAGGTTGACCTCGGATCAGGTAGGAATACCCGCTGAACTTAAGCATATCAATAAGCGGAGGAAAAGAAACCAACAGGG-ATTGCCCCAGTAACGGCGAGTGAAGCGGCAACAGCTCAAATTTGAAATCTGGCCTCGG-CCCGAGTTGTAATTTGCAGAGGAAGCTTCTGGTGCGGTCTGTCCCGAGTCCCCTGGAACGGGGCGCCGGAGAGGGTGAGAGCCCCGTACGGACGGATACCAATCCTGTGTGAAGCTCCTTCGACGAGTCGAGTAGTTTGGGAATGCTGCTCAAAATGGGAGGTAAATTCCTTCTAAAGCTAAATACCGGCCAGAGACCGATAGCGCACAAGTAGAGTGATC-------GAAAGATGAAAAGCACTTTGAAAAGAGGGTTAAACAGCACGTGAAATTGTTGAAAGGGAAGCGCTTGTGACCAGACTTGCGCCAGGTCGATCATCCGGTGTTCTCACCGGTGCACTCGGCCTGGCTCAGGCCAGCATCGGTTCCCGCGGGGGGATAAAGGCCCAGGGAATGTAGCTCCTCCGGGAGTGTTATAGCCCGGGGCGCAATGCCCCCGTGGGGACCGAGGTTCGCGCATCTGCAAGGATGCTGGCGTAATGGTCATCAGCGACCCGTCTTGAAACACGGACCAAGGAGTCAAGGTTTTGCGCGAGTGTTTGGGTGTCAAACCCGCACGCGTAATGAAAGTGAA-CGTAGGTGAGAG----CTTCGGCGCATCATCGACCGATCCTGATGTCTTCGGATGGATTTGAGTAGGAGCGTTAAGCCTTGGACCCGAAAGATGGTGAACTATGCTTGGATAGGGTGAAGCCAGAGGAAACTCTGGTGGAGGCTCGCAGCGGTTCTGACGTGCAAATCGATCGTC?????????????????????????????????????????????????????????GCGCTCAAGTACTCGCTGGCGACTGGAAACTGGGGTGATCAAAAGAAAGCCATGAGCTCCACTGCAGGTGTCTCGCAGGTCCTGAACCGATACACATTTGCCTCTACACTTTCTCACTTGCGGCGAACGAACACACCTATTGGGCGCGATGGCAAGCTCGCAAAACCCCGCCAACTCCATAACACACACTGGGGTCTGGTCTGTCCAGCAAAGACGCCCGAAGGCCAGGCTTGTGGTCTGGTCAAGAATCTCTCGCTGATGTGCTACATTAGCGTTGGTACCAATGCGGATCCTATCATCGAATTCATGATTGCCAGGAACATGGAAGTCCTGGAAGAGTATGAGCCTCTGCGGTATCCGAATGCCACAAAGGTTTTCGTCAATGGCACCTGGGTCGGTGTTCACCAAGACCCAAAGCACCTGGTCAGCTTGGTTCAGAGCCTAAGGAGATCCAATATTATCAGTTTCGAGGTTTCCCTGGTTCGCGACATCCGAGACAGAGAGTTCAAGATCTTCTCCGATGCAGGGCGTGTCATGAGACCGCTATTCGTCGTTGAACAGGAGGATGACGGTGAG---------AGCAAGGTTGAGAAGGGCCAGCTGGTTCTTACCAAGTCGCAAATCCTCAAACTAGAAAAAGACAAGGAGATTGGCAAATACCATCCGGATTACTTCGGCTGGAATGGCCTCTTGAGGGAAGGCTGTGTGGAATACCTGGATGCGGAAGAAGAGGAAACGGCGATGATCTGTATGACGCCCGAGGACCTAGACACGTACCGGCTAGCCAAGCT------------TGGGTTCAACGTCGCCGAGGAGGATCCTAGTGAAGGCAACAAGCGCATCAAGACTAGGCTCAACCCGACGACCCATATGTATACTCACTGCGAGATTCACCCCAGCATGCTGCTTGGTATCTGCGCCAGCATCATTCCCTTCCCCGATCACAACCA?GCTTGTTGACCAAGTTCTTGATGTCGTCCGTCGCGAGGCCGAGGGCTGCGACTGCCTCCAGGGCTTCCAGATCACCCACTCTCTCGGTGGTGGTACCGGTGCCGGTATGGGTACCCTCCTGATCTCCAAGATTCGCGAGGAGTTCCCCGACCGCATGATGGCGACCTTCTCTGTCGTCCCCTCGCCCAAGGTCTCTGATACCGTCGTTGAGCCCTACAACGCCACGCTCTCCGTCCACCAGCTTGTTGAGAACTCCGACGAGACCTTCTGCATTGACAACGAGGCTCTCTACGACATCTGCATGCGGACGCTCAAGCTGTCCAACCCTTCATACGGTGATCTCAACCACCTGGTCTCTGCCGTCATGTCCGGCGTTACCGTTTCCCTGCGCTTCCCCGGCCAGCTCAACTCCGATCTCCGCAAGCTCGCCGTGAACATGGTTCCTTTTCCCCGTCTCCACTTCTTCATGGTCGGCTTCGCGCCGCTTACCAGCCGTGGCGCGCACTCTTTCCGTGCTGTCTCGGTTCCTGAGTTGACCCAGCAGATGTTCGACCCCAAGAACATGATGGCTGCGTCCGACTTCCGCAACGGTCGCTATCTGACCTGCTCTGCCATCTT

>*Apodus*_*deciduus*_CBS_506_70

ATTACAGAGTTGC---------AAAACTCC--CA-ACCCTTTGTGAACG-AACCTA--------CCAGTTGCTTCGGCGG------------------CAGACCCCTCTGG--------------GGCCTAGCCTTT------------------------GCGGGTAC-CCCGCCGGAGT-----CCTAC-AAACTCT----TGATTTT-TTGGCATCTCTGAGT-AGCTTATA-AATAAGTTAAAACTTTC-AACAACGGATCTCTTGGTTCTGGCATCGATGAAGAACGCAGCGAAATGCGATAAGTAATGTGAATTGCAGAATTCAGTGAATCATCGAATCTTTGAACGCACATTGCGCCCGCTAGTATTCTGGCGGGCATGCCTGTTCGAGCGTCATTTCAA-CCATCAAGCCCTA-GGCTT-GTGTTGGGGCCCTGCGG----CCGTCCGCAGCCCCCTAAAAACAGTGGCGGGCTCGCTAT--ATACCGAGTGCAGTAGTTTACTCTTCGCTCAGGACAT-GTAGTGGGT-TCTTGCCGTAAAA---CCCCC--CATTTTTTAAGGTTGACCTCGGATCAGGTAGGAATACCCGCTGAACTTAAGCATATCAATAA????????????????CAACAGGG-ATTGCCCCAGTAACGGCGAGTGAAGCGGCAACAGCTCAAATTTGAAATCTGGCTCCGG-CCCGAGTTGTAATTTGCAGAGGAAGCTTCTGGTGATATACTGTCTAAGTCCCCTGGAACGGGGCGCCACAGTGGGTGAGAGCCCCATAT-GACAGATGTAGATCCTGTGTGAAGCTCCTTCGACGAGTCGAGTAGTTTGGGAATGCTGCTCTAAATGGGAGGTAAATTCCTTCTAAAGCTAAATATTGGCCAGAGACCGATAGCGCACAAGTAGAGTGATC-------GAAAGATGAAAAGCACTTTGAAAAGAGGGTTAAACAGCACGTGAAATTGTTGAAAGGGAAGCGCTTATGACCAGACTTGCGCTGGGCTGATCATCCGGTGTTCTCACCGGTGCACTCGGCCCAGCTCAGGCCAGCATCGGTTTTGGTGGGGGGATAAAGGCGTTGGGAACGTAGCTCCTTCGGGAGTGTTATAGCCCAGCGTGCAATACCCCCGCTGGGACCGAGGTTCGCGCATCTGCAAGGATGCTGGCGTAATGGTCATCAGCGACCCGTCTTGAAACACGGACCAAGGAGTCAAGGTTTTGCGCGAGTGTTTGGGTGTCAAACCCGCACGCGTAATGAAAGTGAA-CGTAGGTGAGAG----CTTCGGCGCATCATCGACCGATCCTGATGTATTCGGATGGATTTGAGTAGGAGCGTTAAGCCTTGGACCCGAAAGATGGTGAACTATGCTTGGATAGGGTGAAGCCAGAGGAAACTCTGGTGGAGGCTCGCAGCGGTTCTGACGTGCAAATCGATCGTCAAATC-TGAGCATGGGGGCGAAAGACTAATCGA????????????????????????????????????????????????????????????????????????????????????????????????????????????????????????????????????????????????????????????????????????????????????????????????????????????????????????????????????????????????????????????????????????????????????????????????????????????????????????????????????????????????????????????????????????????????????????????????????????????????????????????????????????????????????????????????????????????????????????????????????????????????????????????????????????????????????????????????????????????????????????????????????????????????????????????????????????????????????????????????????????????????????????????????????????????????????????????????????????????????????????????????????????????????????????????????????????????????????????????????????????????????????????????????????????????????????????????????????????????????????????????????????????????????????????????????????????????????????????????????????????????????????????????????????????????????????????????????????????????????????????????????????????????????????????????????????????????????????????????????????????????????????????????????????????????????????????????????????????????????????????????????????????????????????????????????????????????????????????????????????????????????????????????????????????????????????????????????????????????????????????????????????????????????????????????????????????????????????????????????????????????????????????????????????????????????????????????????????????????????????????????????????????????????????????????????????????????????????????????????????????

>*Cercophora*_*thailandica*_MFLUCC_12_0845

ATTACAGAGTTGC---------AAAACTCC--CA-ACCCTTTGTGAACC-AACCTA--------CCAGCTGCTCCGGCGG------------------CGAAACTCTTTA--------------------------------------------------------------------------------------------------TA-TTAATATCTCTGATT-AC-TTATTTAATAAGTTAAAACTTTC-AACAACGGATCTCTTGGTTCTGGCATCGATGAAGAACGCAGCGAAATGCGATAAGTAATGTGAATTGCAGAATTCAGTGAATCATCGAATCTTTGAACGCACATTGCGCCCGCCAGTATTCTGGCGGGCATGCCTGTTCGAGCGTCATTTCAA-CCATCAAGCCCTA-GGCTT-GTGTTGGAGCCCTGCGG----CTG-CCGCAGCCTCCCAAAATTAGTGGCGGGCTCGCTAT-TACACCGAGTGCAGTAGTTTACTCTTCGCTCAGGATGT-GTGGCGGGT-GCTAGCCGTGAAA---CCCCCT---ACTCTCAAGGTTGACCTC???????????????????????????????????????????????????????????????????????CCCTAGTAACGGCGAGTGAAGCGGCAACAGCTCAAATTTGAAATCTGGCTCCGG-CCCGAGTTGTAATTTGCAGAGGAAGCTTCTGGTGATATACCGTCTAAGTCCCCTGGAACGGGGTGCCACAGTGGGTGAGAGCCCCATGT-GACGGATGTAGATCCTGTGTGAAGCTCCTTCGACGAGTCGAGTAGTTTGGGAATGCTGCTCAAAATGGGAGGTAAATTCCTTCTAAAGCTAAATATTGGCCAGAGACCGATAGCGCACAAGTAGAGTGATC-------GAAAGATGAAAAGCACTTTGAAAAGAGGGTTAAACAGCACGTGAAATTGTTGAAAGGGAAGCGCTTATGACCAGACTTGCGCTGGGCTGATCATCCGGTGTTCTCACCGGTGCACTCGGCCCAGCTCAGGCCAGCATCGGTTTTGGCGGGGGGATAAAGGCGTAGGGAATGTAGCTCCTCCGGGAGTGTTATAGCCCAACGTGCAATACCCCCGCTGGGACCGAGGTTCGCGC-TCTGCAAGGATGCTGGCGTAATGGTCATCAGCGACCCGTCTTGAAACACGGACCAAGGAGTCAAGGTTTTGCGCGAGTGTTTGGGTGTCAAACCCGCACGCGTAATGAAAGTGAA-CGTAGGTGAGAG----CTTCGGCGCATCATCGACCGATCCTGATGTATTCGGATGGATTTGAGTAGGAGCGTTAAGCCTTGGACCCGAAAGATGGTGAACTATGCTTGGATAGGGTGAAGCCAGAGGAAACTCTGGTGGAGGCTCGCAGCGGTTCTGACGTGCAAATCGATCGTCAAATC-TGAGCATGGGGGCGAAAGACTAATCGAACCATCTAGTAGCTGGTTACCGCCGCTATCAAGTACTCGCTGGCCACCGGCAACTGGCGTGATCAAAAGAAGGCGATGAGCTCCACGGCCGGTGTGTCTCAAGTGTTGAACCGATATACTTTTGCATCGACCCTCTCCCATTTGCGGCGAACCAACACGCCTATCGGTCGCGACGGCAAGCTCGCTAAGCCACGGCAGCTGCACAACACGCACTGGGGCCTTGTCTGCCCTGCCGAGACCCCAGAAGGCCAGGCTTGCGGTCTCGTCAAGAACCTCTCTTTGATGTGTTACATCAGCGTCGGTACCAATGCGGAACCCATCGTCGATTTTATGACAGCCAGGGGGATGGATGTTCTCGAAGAGTACGAGCCGCTGCGGTATCCCAACGCCACCAAGGTCTTCGTCAACGGAACCTGGGTCGGCGTCCACCAAGAGCCTAAACACCTCGTCACCTTGGTCCAGAATCTAAGGCGGACGAGCATCATCTCCTTTGAGGTCTCGCTAGTTAGGGACATTCGTGACCGAGAATTCAAGATCTTCTCGGACGCCGGCCGCGTCATGAGGCCGCTCTTCGTTGTCGAGCAGGAGGAAAAGAGCGAC------ACGTCCAAGGTCCAGAAGGGCCAGCTGGCCCTCACACGGGCACACATGACCCGTCTGGATAGAGACAAGGAGCTCGGGCCCCTTGACGAAGAATACTTTGGTTGGAACGGTCTCCTGAGAGAGGGCTGTGTCGAGTATCTCGACGCCGAGGAAGAGGAGACGGCCATGATTTGCATGACGCCCGAAGATTTGGAACATTATCGCAACACCAAGATGGGCATGTACAAGGACCAGCCCGAGGAGGACCCGGAAAACAAGGAGTATAACAAGCGCATCAAGACGAAGGCGAACCCGACCACGCACATGTACACCCACTGCGAGATCCATCCCAGCATGTTGCTTGGCATCTGCGCGAGCATCATCCCCTCCC????????????????????????????????????????????????????????????????????????????????????????????????????????????????????????????????????????????????????????????????????????????????????????????????????????????????????????????????????????????????????????????????????????????????????????????????????????????????????????????????????????????????????????????????????????????????????????????????????????????????????????????????????????????????????????????????????????????????????????????????????????????????????????????????????????????????????????????????????????????????????????????????????????????????????????????????????????????????????????

>*Morinagamyces*_*vermicularis*_CBS_303_81

ATTACAGAGTTGC---------AAAACTCC--CA--ACCATTGTGAACG-AACCGC-----------TGTGCTCAGGCGG------------------GTGGCCCT--------------------------------------------------------GCTGCCG-CCCGCCGGGAG-----CACGC-AAGCTCT---TTAATTT--CGTGGATATCTGAGT-AGCTCATTCAATGAGTCAAAACTTTC-AACAACGGATCTCTTGGTTCTGGCATCGATGAAGAACGCAGCGAAATGCGATAAGTAATGTGAATTGCAGAATTCAGTGAATCATCGAATCTTTGAACGCACATTGCGCCCGCTAGTATTCTGGCGGGCATGCCTGTTCGAGCGTCATTTCAA-CCATCAAGCCCCG-GGCTT-GTGTTGGGGACCTGCGG----CTG-CCGCAGCCCCCTAAAAGCAGTGGCGGTCTCGCTGT-CACACCGAGCGCAGTAGTGTAC-CTCCGCTCGGGGAGTGGCGGCGGGTTGCCTGCCGTGAAA---CACAC--------CTAAGGTTGACCTCGGATCAGGTAGGAATACCCGCTGAACTTAAGCATATCAATAAGCGGAGGAAAAGAAACCAACAGGG-ATTGCCTCAGTAACGGCGAGTGAAGCGGCAACAGCTCAAATTTGAAATCTGGCTTCGG-CCCGAGTTGTAATTTGCAGAGGAAGCTTCTGGCGCAGCGCCATCCGAGTCCCCTGGAACGGGGCGCCACAGAGGGTGAGAGCCCCGTATGGATGGACGCCTAGCCTGTGTGAAGCTCCTTCGACGAGTCGAGTAGTTTGGGAATGCTGCTCAAAATGGGAGGTAAATTCCTTCTAAAGCTAAATACCGGCCAGAGACCGATAGCGCACAAGTAGAGTGATC-------GAAAGATGAAAAGCACTTTGAAAAGAGGGTTAAATAGCACGTGAAATTGTTGAAAGGGAAGCGCTCATGACCAGACTTGCGCCAGGCTGATCATCCGGTGTTCTCACCGGTGCACTCTGCCTGGCTCAGGCCAGCATCGGTTTCGGCGGGGGGATAAAGGCCTAGGGAACGTAGCTCCTCCGGGAGTGTTATAGCCCTGGGTGCAATGCCCCCGCTGGGACCGAGGTTCGCGC-TCTGCAAGGATGCTGGCGTAATGGTCATCAGCGACCCGTCTTGAAACACGGACCAAGGAGTCAAGGTTTTGCGCGAGTGTTTGGGTGTCAAACCCGCACGCGTAATGAAAGTGAA-CGTAGGTGAGAG----CTTCGGCGCATCATCGACCGATCCTGATGTATTCGGATGGATTTGAGTAGGAGCGTTAAGCCTTGGACCCGAAAGATGGTGAACTATGCTTGGATAGGGTGAAGCCAGAGGAAACTCTGGTGGAGGCTCGCAGCGGTTCTGACGTGCAAATCGATCGTCATAATC???????????????????????????????????????????????????GGCCTGAAGTACTCGCTCGCCACTGGCAACTGGGGTGACCAGAAGAAGGCCATGAGCTCCACCGCTGGCGTGTCCCAGGTCTTGAACCGATACACCTTCGCCTCGACCCTCTCTCACTTGCGGCGAACCAACACCCCCATCGGCCGCGACGGGAAGCTGGCGAAACCCCGTCAGCTCCACAACACGCACTGGGGCTTGGTCTGCCCTGCCGAGACTCCCGAAGGCCAGGCCTGCGGTCTGGTCAAGAACCTGTCTCTCATGTGTTACATCAGTGTGGGCACTAATGCTGAACCCATTATCGACTTCATGGTTGCTAGGAACATGGAAGTACTCGAAGAATACGAACCTTTGAGGTACCCCAATGCCACGAAAGTCTTTGTCAATGGAACCTGGGTCGGTATCCATCAAGAGCCTAAGCATCTGGTCAACCTTGTCCAGGGCCTGAGACGACTCAACATTATCTCTTTTGAGGTCTCGCTTGTTAGAGATATCCGAGACCGGGAGTTCCAGATCTTCTCGGACGCCGGCCGTGTCATGCGACCGCTGTTCGTTGTCGCGCAGGAAGCGGATCCCGAG---------CGAAAACTCGAGCAGGGCCAGCTCGTCCTCACAAAGGAACATATCCGCCGTCTGGAACATGACAAGGAGATTGGCCGAGAGCACCCCGACCATTTCGGCTGGGATGGTTTGCTTCGAGAGGGGTGCGTCGAGTATCTCGATGCCGAAGAGGAGGAGACGTCGATGATCTGCATGTCGCCCGAGGACTTGTCCGACTATCGACTCACCAAGCT------------TGGTTTCCACGTTGTCGAGGAAGACTCGAGTGAAGGCAATCGGCGCATCAAGACCAAGATGAACCCCACGACACACATGTACACGCATTGCGAGATCCACCCCAGCATGTTGCTCGGCATTTGTGCTAGCATCATTCCCTTCCCCGACCACAACCAGGCTTGTTGACCAAGTTCTCGATGTCGTCCGTCGCGAGGCTGAGGGTTGCGACTGCCTCCAGGGCTTCCAGATCACCCACTCCCTCGGTGGTGGTACCGGTGCCGGTATGGGTACGCTCCTCATCTCCAAGATCCGCGAGGAGTTCCCCGACCGCATGATGGCCACTTTCTCGGTCGTCCCCTCGCCCAAGGTGTCAGATACCGTCGTCGAGCCTTACAACGCCACTCTTTCGGTCCACCAGCTCGTTGAGAACTCGGACGAGACCTTCTGCATTGACAACGAGGCTCTGTACGACATTTGCATGAGGACTCTGAAGCTGTCCAACCCCTCGTACGGCGACCTTAACCACCTGGTCTCGGCTGTCATGTCGGGCGTCACTGTCTCGCTGCGCTTCCCCGGTCAGCTCAACTCGGACCTCCGCAAGCTTGCTGTCAACATGGTTCCTTTCCCGCGTCTCCACTTCTTCATGGTTGGCTTCGCTCCTCTTACCAGCCGTGGCGCGCACTCTTTCCGTGCCGTTTCGGTTCCTGAGCTCACGCAGCAAATGTTCGACCCCAAGAACATGATGGCTGCTTCTGACTTCCGCAACGGTCGCTACCTTACATGCTCTGCCATCTT

>*Naviculispora*_*terrestris*_CBS_137295

ATTAAAGAGTTTC---------AAAACTCCCATA-AACCATCGCGAACGTTACCA------CATGTCGTTGCTTCGGCTA------------------GCAGGCGAGCCCC-------TCACCGGGGGCAGAGCCTCATTGCGGCCCCTC-----------ACGGGGCG-CTAGCCGGAGGGACT-AAAAC-AAACCCT-TGCATTTA---TTGGCACCTCTGAGT-ATGATTTTAAATAAGTCAAAACTTTCAAACAACGGATCTCTTGGTTCTGGCATCGATGAAGAACGCAGCGAAATGCGATAAGTAATGTGAATTGCAGAATTCAGTGAATCATCGAATCTTTGAACGCACATTGCGCCCGCCAGTATTCTGGCGGGCATGCCTGTTCGAGCGTCATTTCAACCCATCAAGCCTT--AGCTT-GTGCTGGGGCCCTGCGGA--TCTACCCGCAGGCCCTGAAAATCAGTGGCGGGCTCGCTAGTCACACCGAGCGCAGTAG--CACATCTCGCTTTGGTCGT-GCGGCGGGT-TCTTGCCGTTAAA---CACCC--CATTTTTTAAGGTTGACCTCGGATCAGTA?????????????????????????????????????????AAAGAAACCAACAGGG-ATTGCCCTAGTAACGGCGAGTGAAGCGGCAACAGCTCAAATTTGAAATCTGGCTTCGG-CCCGAGTTGTAATTTGTAGAGGAAGCTTTTGGCACGGCACCTACTGAGTCCCCTGGAACGGGGCGCCATAGAGGGTGAGAGCCCCGTATAGTAGGATGCCTAGCCTGTGTAAAGCTCCTTCGACGAGTCGAGTAGTTTGGGAATGCTGCTCAAAATGGGAGGTAAATTTCTTCTAAAGCTAAATATTGGCCAGAGACCGATAGCGCACAAGTAGAGTGATC-------GAAAGATGAAAAGCACTTTGAAAAGAGGGTTAAACAGCACGTGAAATTGTTGAAAGGGAAGCGCTTGTGACCAGACTTGCGCCCGGCTGATCATCCGGTGTTCTCACCGGTGCACTCTGCCGGGCTCAGGCCAGCATCGGTTTTCGTGGGGGGATAAAGACCTGGGGAACGTAGCTCTTCCGGGAGTGTTATAGCCCTGGGTGCAACGCCCTCGCGGGGACCGAGGTTCGCGCATCTGCAAGGATGCTGGCGTAATGGTCATCAGCGACCCGTCTTGAAACACGGACCAAGGAGTCAAGGTTTTGCGCGAGTGTTTGGGTGTTAAACCCGCACGCGTAATGAAAGTGAA-CGTAGGTGAGAG----CTTCGGCGCATCATCGACCGATCCTGATGTATTCGGATGGATTTGAGTAGGAGCGTTAAGCCTTGGACCCGAAAGATGGTGAACTATGCTTGGATAGGGTGAAGCCAGAGGAAACTCTGGTGGAGGCTCGCAGCGGTTCTGACGTGCAAATCGATCGTCAAATC-TGAGCATGGGGGCGAAAGACTAATCGAACCATCTAGTAGCTGGTTACCGC?GCCCTGAAATACTCCCTCGCCACTGGCAACTGGGGAGACCAGAAGAAGGCCATGAGCTCGACGGCCGGTGTTTCCCAGGTGTTGAACAGATATACCTTCGCCTCCACACTTTCTCACTTGAGACGCACGAACACCCCGATTGGTCGTGACGGTAAACTGGCAAAACCGCGCCAGTTGCACAACACACATTGGGGTTTGGTCTGTCCCGCTGAGACGCCCGAAGGACAGGCTTGCGGTCTTGTTAAGAATTTGTCCCTCATGTGTTATGTGAGTGTGGGAACCACTGCAGACCCTATCGTGGACTTTATGATTGCCCGAAACATGGAAGTTCTTGAGGAGTACGAGCCTCTTCGTCATCCCAATGCGACTAAAGTCTTTGTCAACGGATCGTGGGTTGGTGTGCATCATGATGCCAAGCATCTGGTTAGTTTGGTGCAATCTTTGCGCCGGAAGGGAACCATCTCGTTCGAGATCTCCCTCGTCAGAGATATTCGTGACAGAGAGTTCAAGATCTTCTCTGATGCTGGCCGAGTCATGAGACCTCTGTTTGTCGTCGAGACCGAAGATAATAGCGAC---------AGTGGTGTGGACAAAGGATCATTGGTCCTTACCAAGTCCCATGTTAAGCGCTTGGAGGAAGACAAAGAGCTTGGCAAATACCATCCTGATTATTGGGGCTGGGAGGGCCTCAAGGCGTCTGGTGCCATCGAATATCTGGACGCTGAGGAGGAAGAGACCACCATGATCTGCATGACGCCAGAGGATTTGGATTCCTACCGCCTTTTCAAGCTGGCAAAGCAAGGAGGGTTCGACCTTGACGAGGAGAATGCGCACGGCGCCAATAGCCGTATCAAGACGAAGCTGAACCCCACAACCCATGCTTATACTCACTGCGAAATCCATCCTAGCATGCTGCTAGGAATTTGCGCGAGTATCATTCCGTTCCCTGATCATAACCAGGCTTGTTGACCAAGTTCTCGATGTCGTCCGTCGCGAGGCCGAGGGCTGCGACTGCCTCCAGGGTTTCCAGATCACCCACTCTCTCGGTGGTGGTACTGGTGCCGGTATGGGTACCCTCCTGATCTCCAAGATCCGCGAGGAGTTCCCCGACCGCATGATGGCCACCTTCTCGGTTGTCCCCTCCCCCAAGGTCTCTGACACCGTTGTCGAGCCCTACAACGCCACCCTCTCCGTGCACCAGCTCGTTGAGAACTCTGACGAGACCTTCTGCATTGACAACGAGGCTCTCTACGACATTTGCATGCGCACCCTTAAGCTCTCCAACCCCTCGTATGGCGACCTTAACCACCTTGTTTCCGCCGTCATGTCTGGTGTCACGGTCTCTCTCCGTTTCCCCGGTCAGCTCAACTCCGACCTCCGCAAGCTTGCCGTCAACATGGTTCCCTTCCCCCGTCTGCACTTCTTCATGGTCGGCTTTGCGCCCCTTACTAGCCGTGGCGCTCACTCTTTCCGTGCGGTTTCTGTCCCTGAGCTCACCCAGCAGATGTTCGACCCCAAGAACATGATGGCTGCCTCTGACTTCCGTAACGGTCGCTACCTGACTTGCTCTGCCATCTT

>*Podospora*_*bullata*_CBS_115576

ATTAGCGAGTTGC---------AAGACTCC--CAAACCCTCTGTGAATG-CCCTGG--------ACCGCTGCTTCGGCGG------------------TCGGGAGCCCCTA--------------------------------------------------GACGGGCG-ACCGCCGCCGGGCGACAACAC-CAACTCT-TGTATTTTT--CTGGCATCTCTGAGT--ACTCTTATAATGAGTCAAAACTTTC-AACAACGGATCTCTTGGTTCTGGCATCGATGAAGAACGCAGCGAAATGCGATAAGTAATGTGAATTGCAGATTTCAGTGAATCATCGAATCTTTGAACGCACATTGCGCCCGCCAGTATTCTGGCGGGCATGCCTGTTCGAGCGTCATTTCAACCCATCAAGCCCCTGCGCTT-GCGTTGGAGCCCTGCGG----CCG-CCGCAGCCTCCCAAAGACAGTGGCGGGCTCGCTAT-CACACCGAGTGCAGTAGATTTCTCCTCGCTCAGGGCGT-GTGGCGGGT-GCCGGCCGTGAAA---CCCCCCAAGCTTTCAAAGGTTGACCTCGGATCAGGTAGGAATACCCGCTGAACTTAAGCATATCAATAAGCGG???????????????????????????????????GGCGAGTGAAGCGGCAACAGCTCAAATTTGAAATCTGGCTCCGG-CCCGAGTTGTAATTTGCAGAGGAAGCTTCTGGTGACGCGCCGTCTAAGTCCCCTGGAACGGGGCGCCGCAGCGGGTGAGAGCCCCATCT-GACGACCGCTGACCCAGTGTGAAGCTCCTTCGACGAGTCGAGTAGTTTGGGAATGCTGCTCAAAATGGGAGGTAAATTCCTTCTAAAGCTAAATACCGGCCAGAGACCGATAGCGCACAAGTAGAGTGATC-------GAAAGATGAAAAGCACTTTGAAAAGAGGGTTAAACAGCACGTGAAATTGTTGAAAGGGAAGCGCTCATGACCAGACTTGCGCTGGGCTGATCATCCGGTGTTCTCACCGGTGCACTCTGCCCAGCTCAGGCCAGCATCGGTTTCGGCGGGGGGATAAAGGCGACGGGAACGTAGCTCCTCCGGGAGTGTTATAGCCCGGCGTGCAATACCCCCGCTGGGACCGAGGTCCGCGC-TCTGCAAGGATGCTGGCGTAATGGTCATCAGCGACCCGTCTTGAAACACGGACCAAGGAGTCAAGGTTTTGCGCGAGTGTTTGGGTGTCAAACCCGCACGCGTAATGAAAGTGAA-CGTAGGTGAGAG----CTTCGGCGCATCATCGACCGATCCTGATGTTCTCGGACGGATTTGAGTAGGAGCGTTAAGCCTTGGACCCGAAAGATGGTGAACTATGCTTGGATAGGGTGAAGCCAGAGGAAACTCTGGTGGAGGCTCGCAGCGGTTCTGACGTGCAAATCGATCGTCAAATC-TGAGCAT????????????????????????????????????????????????????????????????????????????????????????????????????????????????????????????????????????????????????????????????????????????????????????????????????????????????????????????????????????????????????????????????????????????????????????????????????????????????????????????????????????????????????????????????????????????????????????????????????????????????????????????????????????????????????????????????????????????????????????????????????????????????????????????????????????????????????????????????????????????????????????????????????????????????????????????????????????????????????????????????????????????????????????????????????????????????????????????????????????????????????????????????????????????????????????????????????????????????????????????????????????????????????????????????????????????????????????????????????????????????????????????????????????????????????????????????????????????????????????????????????????????????????????????????????????????????????????????????????????????????????????????????????????????????????????????????????????????????????????????????????????????????????????????????????????????????????????????????????????????????????????????????????????????????????????????????????????????????????????????????????????????????????????????????????????????????????????????????????????????????????????????????????????????????????????????????????????????????????????????????????????????????????????????????????????????????????????????????????????????????????????????????????????????????????????????????????????????????????????????????????????????????????????????

>*Podospora*_*communis*_CBS_118393

ATTACAGAGTTGC---------AAAACTCC--CACACCCTTTGTGAACG-CACTGGA-------ACTACTGCTTCGGCGG------------------GCAGCTCTTCAG----------------------------------------------------TGGGCAG-CCCGCCGGAGA-----AGCACTAAACTCT----TGTATTT-TTGGCTTCTCTGAGT-GA-TTATACAATGAATCAAAACTTTC-AACAACGGATCTCTTGGTTCTGGCATCGATGAAGAACGCAGCGAAATGCGATACGTAATGTGAATTGCAGAATTCAGTGAATCATCGAATCTTTGAACGCACATTGCGCCTGCCAGCACTCTGGCAGGCATGCCTGTTCGAGCGTCATTTCAA-CCATCAAGCCCTA-GGCTT-GCGTTGGAGCCCTGCGG----CTG-CCGCAGGCCCCTAAAAGCAGTGGCGGGCTCGCTAT-CACACCGAGTGCAGTAGATTACTCTTCGCTCAGGGCGT-GTGGCGGGT-TCCAGCCGTGAAA----CCCC--TACTTCTCAAGGTTGACCTCGGATCAGGTAGGAATACCCGCTGAACTTAAGCATATCAA???????????AAAGGCCCCAACAGGG-ATTGCCTCACCAACGGCGAGTGAAGCGGCAACAGCTCAAATTTGAAATCTGGCTCCGGACCCGAGTTGTAATTTGCAGAGGAAGCTTCTGGTGATGCACCGTCTAAGTCCCCTGGAACGGGGCGCCACAGTGGGTGAGAGCCCCATTT-GACGGACGCTGAGCCTGTGTGAAGCTCCTTCGACGAGTCGAGTAGTTTGGGAATGCTGCTCAAAATGGGAGGTAAATTCCTTCTAAAGCTAAATATTGGCCAGAGACCGATAGCGCACAAGTAGCAAGATGAGTGATCGAAAGATGAAAAGCACTTTGAAAAGAGGGTTAAACAGCACGTGAAATTGTTGAAAGGGAAGCGCTTGTGACCAGACTTGCGCTGGGTTGATCATCCGGTGTTCTCACCTGTGCACTCGGCCCACCTCAAGCCAGCATCGGTTTTGGCGGGGGGATTAAGGCGCTGGCAACGTATCTCCCCGGGGAGAGTTATAGGCCAGCGTGCAATGCCCCCGGTGGGACCTAGGTTCGTGCATCTGCAAGGATGCGGGAGGAATGGTAATCAGCGACCCGTCTTGAAACACGCACCAAGCAGTGAGGGATGTGTTCGAGTGTTTGGGTGTCAATCCCGACCGCGTAAGGAAAG-AAAGTGGAGGTGGGAAACAGCTTGGGTGCATCATCGACCGATCCTGATGTATTCGGAAGGATTTGAGTAAGAGCATGGATCCTGGGACCCGAAAGATGGTGAACTATGCTTGAATAGGGTGAAGCCAGAGGAAACTCTGGTGGAGGCTCGCAGCGGTTCTGACGTGCAAATCGATCGTCTAATC-CGCGCATGGGGGCGAAAGACTAATCGAACCATCTAGTAGCTGGTTACCGCC????????????????????????????????????????????????????????????????????????????????????????????????????????????????????????????????????????????????????????????????????????????????????????????????????????????????????????????????????????????????????????????????????????????????????????????????????????????????????????????????????????????????????????????????????????????????????????????????????????????????????????????????????????????????????????????????????????????????????????????????????????????????????????????????????????????????????????????????????????????????????????????????????????????????????????????????????????????????????????????????????????????????????????????????????????????????????????????????????????????????????????????????????????????????????????????????????????????????????????????????????????????????????????????????????????????????????????????????????????????????????????????????????????????????????????????????????????????????????????????????????????????????????????????????????????????????????????????????????????????????????????????????????????????????????????????????????????????????????????????????????????????????????????????????????????????????????????????????????????????????????????????????????????????????????????????????????????????????????????????????????????????????????????????????????????????????????????????????????????????????????????????????????????????????????????????????????????????????????????????????????????????????????????????????????????????????????????????????????????????????????????????????????????????????????????????????????????????????

>*Podospora*_*cupiformis*_CBS_246_71

ATTACCGAGTTCCAG-------TGGACTCC--CA-ACCCTGTGTGATCG-CACCTGCCATACCACATGTTGCCTCGGCGG----------------TAGTGGTCGAGCCTA----------------CCCAGTCATC-----------------TGGCGAGGGCTCCCA-CCCGCCGGGCG-----CTACA-AAACACTCTGTAACGTT--GTGGCATCTCTGAGT-GGCTTCTATAATAAGTCAAAACTTTC-AACAACGGATCTCTTGGTTCTGGCATCGATGAAGAACGCAGCGAAATGCGATAAGTAATGTGAATTGCAGAATTCAGTGAATCATCGAATCTTTGAACGCACATTGCGCCCGCCAGTATTCTGGCGGGCATGCCTGTCCGAGCGTCATTTCAACCCATCAAGCCC---AGCTT-GTGTTGGAGCCCTGCGG----CCG-CCGCAGCCTCCCAAAAACAGTGGCGGGCTCGCCAT-CACACTGAGTGCAGTAGTGTTCTCCTCGCTCCTGTGTC-GTGGCGGGT-TCCGGCCGTGAAA---CCCCCCCAATTATTCAAGGTTGACCTCGGATCAGGTAGGAATACCCGCTGAACTTAAGCATATCAATAAGCGGAGGA????????CAACTGGG-ATTGCCCTAGTAACGGCGAGTGAAGCGGCAACAGCTCAAATTTGAAATCTGGCTCCGG-CCCGAGTTGTAATTTGCAGAGGAAGCTTCTGGCGACGCGCCGCCTAAGTCCCCTGGAATGGGGCGCCACAGCGGGTGAGAGCCCCGTGT-GACGGCTGCTGAACCAGTGTGAAGCTCCTTCGACGAGTCGAGTAGTTTGGGAATGCTGCTCAAAATGGGAGGTAAATTCCTTCTAAAGCTAAATACCGGCCAGAGACCGATAGCGCACAAGTAGAGTGATC-------GAAAGATGAAAAGCACTTTGAAAAGAGGGTTAAACAGCACGTGAAATTGTTGAAAGGGAAGCGCTTATGACCAGACTTGCGCTGGGCTGATCATCCGGTGTTCTCACCGGTGCACTCTGCCCAGCGCAGGCCAGCATCGGTTTCGGCGGGGGGATAAAGGCGCTGGGAACGTAGCTCCCCCGGGAGTGTTATAGCCCAGCGTGCAATGCCCCCGCCGGGACCGAGGACAGCGC-CCTGCGAGGATGCTGGCGTAATGGTCATCAGCGACCCGTCTTGAAACACGGACCAAGGAGTCAAGGTTTTGCGCGAGTGTTTGGGTGTCAAACCCGCACGCGTAATGAAAGTGAA-CGTAGGTGAGAG----CTTCGGCGCATCATCGACCGATCCTGATGTTCTCGGATGGATTTGAGTAGGAGCGTTAAGCCTTGGACCCGAAAGATGGTGAACTATGCTTGGATAGGGTGAAGCCAGAGGAAACTCTGGTGGAGGCTCGCAGCGGTTCTGACGTGCAAATCGATCGTCAAATC-TGAGCATGGGGGCGAAAGACTAATCGA????????????????????????????????????????????????????????????????????????????????????????????????????????????????????????????????????????????????????????????????????????????????????????????????????????????????????????????????????????????????????????????????????????????????????????????????????????????????????????????????????????????????????????????????????????????????????????????????????????????????????????????????????????????????????????????????????????????????????????????????????????????????????????????????????????????????????????????????????????????????????????????????????????????????????????????????????????????????????????????????????????????????????????????????????????????????????????????????????????????????????????????????????????????????????????????????????????????????????????????????????????????????????????????????????????????????????????????????????????????????????????????????????????????????????????????????????????????????????????????????????????????????????????????????????????????????????????????????????????????????????????????????????????????????????????????????????????????????????????????????????????????????????????????????????????????????????????????????????????????????????????????????????????????????????????????????????????????????????????????????????????????????????????????????????????????????????????????????????????????????????????????????????????????????????????????????????????????????????????????????????????????????????????????????????????????????????????????????????????????????????????????????????????????????????????????????????????????????????????????????????????

>*Pseudorhypophila*_*mangenotii*_CBS_419_67

????????????????????????????????CA-AACCATCGCGAACGTTACCCAGAC-----GTCGTTGCTTCGGCGG------------------------------------------------------------------------------------GCTCG-CCCGCCGGGGGGACG-CTACC-AGACTCT-TGCAGTTAT--TCGGCCTCTCTGAGTACGATTTTTAAATAAGTCAAAACTTTC-AACAACGGATCTCTTGGTTCTGGCATCGATGAAGAACGCAGCGAAATGCGATAAGTAATGTGAATTGCAGAATTCAGTGAATCATCGAATCTTTGAACGCACATTGCGCCCGCCAGTATTCTGGCGGGCATGCCTGTTCGAGCGTCATTTCAA-CCTTCAAGCCC---TGCTT-GTGTTGGGGTCCTGCGG----CTGCCCGCAGGCCCTGAAAACCAGTGGCGGGCTCGCTAGTCACACCGAGCGCAGTAG--CACATCTCGCTCAGGGCGT-GCGGCGGGT-TCTTGCCGTTAAA---CACCCCCCCTTTCCACAGGTTGACCTCGGATCAGGTAGGAATACCCGCTGAACTTAAGCATATCAATAAGCGGAGGAAAAGAAACCAACAGGG-ATTGCCCTAGTAACGGCGAGTGAAGCGGCAACAGCTCAAATTTGAAATCTGGCTTCGG-CCCGAGTTGTAATTTGTAGAGGAAGCTTTTGGCGCGGCACCTACTGAGTCCCCTGGAACGGGGCGCCAGAGAGGGTGAGAGCCCCGTATAGTGGGATGCCTAGCCTCTGTAAAGCTCCTTCGACGAGTCGAGTAGTTTGGGAATGCTGCTCAAAATGGGAGGTAAATTTCTTCTAAAGCTAAATATTGGCCAGAGACCGATAGCGCACAAGTAGAGTGATC-------GAAAGATGAAAAGCACTTTGAAAAGAGGGTTAAACAGCACGTGAAATTGTTGAAAGGGAAGCGCTTGTGACCAGACTTGCGCCCGGTTGATCATCCGGTGTTCTCACCGGTGCACTCCTCCGGGCTCAGGCCAGCATCGGTTCTCGCGGGGGGATAAAGGCCCAGGGAACGTAGCTCTTCCGGGAGTGTTATAGCCCTGGGTGCAATGCCCTCGCGGGGACCGAGGTTCGCGCATCTGCAAGGATGCTGGCGTAATGGTCATCAGCGACCCGTCTTGAAACACGGACCAAGGAGTCAAGGTTTTGCGCGAGTGTTTGGGTGTCAAACCCGCACGCGTAATGAAAGTGAA-CGTAGGTGAGAG----CTTCGGCGCATCATCGACCGATCCTGATGTATTCGGATGGATTTGAGTAGGAGCGTTAAGCCTTGGACCCGAAAGATGGTGAACTATGCTTGGATAGGGTGAAGCCAGAGGAAACTCTGGTGGAGGCTCGCAGCGGTTCTGACGTGCAAATCGATCGTCAAATC-TGAGCATGGGGGCGAAAGACTAATCGAACCATCTAGTAGCTGGTTACCGCGGCCCTCAAGTACTCTCTTGCCACTGGTAACTGGGGAGACCAGAAGAAGGCCATGAGTTCCACAGCCGGTGTATCTCAGGTGTTGAACCGATATACTTTTGCCTCCACCTTGTCGCATTTGAGACGTACCAACACCCCTATTGGTCGTGACGGTAAGCTGGCGAAACCGCGCCAACTCCACAACACTCATTGGGGCCTGGTTTGTCCAGCCGAGACGCCCGAAGGACAGGCTTGCGGCCTCGTCAAGAATCTTTCACTCATGTGCTATGTCAGCGTGGGAACCGTGGCAGACCCTATTGTGGACTTCATGACCGCGCGTGGCATGGAGGTTCTTGAAGAGTACGAACCTCTTCGTTATCCCAACGCCACGAAAGTTTTCGTCAACGGCGCATGGGTCGGCGTCCATCATGATCCCAAGAATCTCGTGTCGTTGGTGCAGGCCCTTCGTCGTAAGAATACGATCAGTTTTGAGATTTCCCTCGTCAGAGAGATCCGTGATCGAGAGTTCAAGATCTTTTCTGATGCTGGTCGTGTGATGAGGCCCCTGTTCGTTGTTGAGACGGAAGACAACGGTATG---------CATGGCGTAGAAAAGGGAAATCTGGTGCTTACCAAACAACATATTAGGAACCTGGAGAGGGACAAGGAACTGGGCAAGTACCACCCTGACTACTGGGGTTGGGAGGGTCTCAAGGCCTCGGGAGCCATTGAATATCTTGACGCCGAGGAAGAGGAGACAACGATGATCTGCATGACTCCGGAAGATCTCGAAAACTACCGTCTTACGAAGAT------TGCTGGTCTCGATCATGTACCGGAGGACACGTCGGGTGGCGCTAATAGCCGTATCAAGACCAGATTGAACCCTACGACCCATGCCTATACCCATTGCGAAATTCACCCAAGTATGCTATTGGGAAT?????????????????????????????????????GCTTGTTGACCAAGTTCTTGATGTCGTCCGTCGTGAGGCCGAGGGCTGCGACTGCCTCCAGGGTTTCCAGATCACCCACTCCCTTGGTGGTGGTACCGGTGCCGGTATGGGTACCCTCCTGATCTCCAAGATCCGTGAGGAGTTCCCTGACCGCATGATGGCTACTTTCTCCGTCGTTCCGTCCCCCAAGGTCTCCGACACCGTTGTCGAGCCCTACAACGCCACCCTCTCCGTGCATCAGCTCGTTGAGAACTCCGACGAGACCTTCTGCATTGACAACGAGGCTCTCTACGACATTTGCATGCGCACCCTTAAGCTCTCCAACCCCTCTTATGGCGACCTTAACCACCTGGTTTCTGCCGTCATGTCTGGTGTCACCGTTTCCCTCCGTTTCCCCGGCCAGCTTAACTCGGACCTTCGCAAGCTCGCCGTCAACATGGTTCCTTTCCCCCGTCTGCACTTCTTCATGGTTGGCTTTGCGCCCCTTACTAGCCGCGGCGCTCACTCTTTCCGTGCGGTGTCAGTTCCTGAGCTCACCCAGCAGATGTTCGACCCCAAGAACATGATGGCTGCTTCTGACTTCCGCAACGGTCGCTACCTCACCTGCTCGGCCATCTT

>*Podospora*_*curvicolla*_CBS_259_69

ATTACCGAGTTCT---------TACAGAACC-CA-ACCCTGTGTGATAA-GTTCTCACCC---GAACTTTTCCACTACAG------------------GTCTCGGTCCACG----------GGCAGGCAGCGCCGG-------CCGCGGT------GGACTTCGAGGCA-CCTGTAGGA-------AACCC-AAACCTC-TGTATCATTAACACAAATATCTGAGCGACTTTACATAAAGAGTCAAAACTTTC-AACAACGGATCTCTTGGTTCTGGCATCGATGAAGAACGCAGCGAAATGCGATACGTAATGTGAATTGCAGAATTCAGTGAATCATCGAATCTTTGAACGCACATTGCGCCCGCCAGTATTCTGGCGGGCATGCCTGTCCGAGCGTCATTTCAACCCATCAAGCCCAG-CGCTT-GTGTTGGAGCCCTGCGG----CCG-CCGCAGGCTCCCAAAATTAGTGGCGGGCTCGCTAT-CACGCTGAGTGCAGTAGTATTCTTCTCACTCCTGCGGT-GTAGCGGGTAACCAGCCGTAAAA---CCCTT----TTACCTAAAGTTGACCTCGGATCAGGTAGGAATACCCGCTGAACTTAAGCATATCAATAAG???????AAAGAAACCAACAGGG-ATTGCCCTAGTAACGGCGAGTGAAGCGGCAACAGCTCAAATTTGAAATCTGGCAACAG-CCCGAGTTGTAATTTGCAGAGGAAGCTTCTGGCGACGCACTGTCTAAGTCCCCTGGAACGGGGCGCCACAGCGGGTGAGAGCCCCATGT-GATGGCTGCGGACCCAGTGTGAAGCTCCTTCGACGAGTCGAGTAGTTTGGGAATGCTGCTCAAAATGGGAGGTAAATTCCTTCTAAAGCTAAATATTGGCCAGAGACCGATAGCGCACAAGTAGAGTGATC-------GAAAGATGAAAAGCACTTTGAAAAGAGGGTTAAACAGCACGTGAAATTGTTGAAAGGGAAGCGCTCATGACCAGACTTGCGCTGGGCTGATCATCCGGTGTTCTCACCGGTGCACTCTGCCCGGCTCAGGCCAGCATCGGTTTTGGTGGGGGGATAAAGGCGCCGGGAACGTAGCTCCTCCGGGAGTGTTATAGCCCGGTGTGCAATACCCCCGCTGGGACCGAGGTCCGCGCATC-GCAAGGATGCTGGCGTAATGGTCATCAGCGACCCGTCTTGAAACACGGACCAAGGAGTCAAGGTTTTGCGCGAGTGTTTGGGTGTCAAACCCGCACGCGTAATGAAAGTGAA-CGTAGGTGAGAG----CTTCGGCGCATCATCGACCGATCCTGAAGTTTACGGACGGATTTGAGTAGGAGCGTTAAGCCTTGGACCCGAAAGATGGTGAACTATGCTTGGATAGGGTGAAGCCAGAGGAAACTCTGGTGGAGGCTCGCAGCGGTTCTGACGTGCAA-TCGATCGTCAA-TC-TGA????????????????????????????????????????????????????????????????????????????????????????????????????????????????????????????????????????????????????????????????????????????????????????????????????????????????????????????????????????????????????????????????????????????????????????????????????????????????????????????????????????????????????????????????????????????????????????????????????????????????????????????????????????????????????????????????????????????????????????????????????????????????????????????????????????????????????????????????????????????????????????????????????????????????????????????????????????????????????????????????????????????????????????????????????????????????????????????????????????????????????????????????????????????????????????????????????????????????????????????????????????????????????????????????????????????????????????????????????????????????????????????????????????????????????????????????????????????????????????????????????????????????????????????????????????????????????????????????????????????????????????????????????????????????????????????????????????????????????????????????????????????????????????????????????????????????????????????????????????????????????????????????????????????????????????????????????????????????????????????????????????????????????????????????????????????????????????????????????????????????????????????????????????????????????????????????????????????????????????????????????????????????????????????????????????????????????????????????????????????????????????????????????????????????????????????????????????????????????????????????????????????????????????????????

>*Schizothecium*_*conicum*_CBS_434_50

ATTACAGAGTTGC---------AAAACTCC--CA--ACCATTGTGAACC-TACCTC--------ACCGTTGCTTCGGCGG------------------GTGGCCCCTCTC-----------CGGGGGCCGCGCCGGGCC----CCACCCC--------GGGCCCGGCAA-CCCGTCAGAGG-----ACCGT-AACTCTT---AGTCATCA-TTGGCCTCTCTGAGT-AACTTATACAATAAGTCAAAACTTTC-AACAACGGATCTCTTGGTTCTGGCATCGATGAAGAACGCAGCGAAATGCGATACGTAATGTGAATTGCAGATTTCAGTGAATCATCGAATCTTTGAACGCACATTGCGCCCGCCAGTATTCTGGCGGGCATGCCTGTTCGAGCGTCATTTCAA-CCATCAAGCCCCC-GGCTT-GTGTTGGAGCCCTGCGG----CTG-CCGCAGGCTCCCAAATCCAGTGGCGGGCTCGTCGT-CGTACCGAGTGCAGTAA--ACATCCTCGCTCAGGGCAC-GTGACGGGT-TCCGGCCGTGAAA---CCCCC-ACTTATATCAAGGTTGACCTCGGATCAGGTAGGAATACCCGCTGAACTTAAGCATATCAATAAGCGGAGGAAAAGAAACCAACAGGG-ATTGCCCCAGTAACGGCGAGTGAAGCGGCAACAGCTCAAATTTGAAATCTGGCCTCGG-CCCGAGTTGTAATTTGTAGAGGAAGCTTCTGGTGCGGCGCCGTCCGAGTCTCCTGGAACGGAGCGCCATAGAGGGTGAGAGCCCCGTATGGACGGATGCCAAACCTGTGCGAAGCTCCTTCGACGAGTCGAGTAGTTTGGGAATGCTGCTCTAAATGGGAGGTAAATTCCTTCTAAAGCTAAATATTGGCCAGAGACCGATAGCGCACAAGTAGAGTGATC-------GAAAGATGAAAAGCACTTTGAAAAGAGGGTTAAACAGCACGTGAAATTGTTGAAAGGGAAGCGCTTGTGACCAGACTTGAGCGCGGCGGATCATCCGGTGTTCTCACCGGTGCACTCTGCCGCGCCCAGGCCAGCATCGGTTTCCGCCGGGGGACAAAGGCACCGGGAACGTAGCTCCTCCGGGAGTGTTATAGCCCGGGGCGTAATGCCCTAGCGGGGACCGAGGACCGCGCATCTGCAAGGATGCTGGCGTAATGGTCATCAGCGACCCGTCTTGAAACACGGACCAAGGAGTCAAGGTTTTGCGCGAGTGTTTGGGTGTTAAACCCGCACGCGTAATGAAAGTGAA-CGTAGGTGAGAG----CTTCGGCGCATCATCGACCGATCCTGATGTATTCGGATGGATTTGAGTAGGAGCGTTAAGCCTTGGACCCGAAAGATGGTGAACTATGCTTGGATAGGGTGAAGCCAGAGGAAACTCTGGTGGAGGCTCGCAGCGGTTCTGACGTGCAAATCGATCGTCAAATC-TGAGCAT????????????????????????????????????????????????????????????????????????????????????????????????????????????????????????????????????????????????????????????????????????????????????????????????????????????????????????????????????????????????????????????????????????????????????????????????????????????????????????????????????????????????????????????????????????????????????????????????????????????????????????????????????????????????????????????????????????????????????????????????????????????????????????????????????????????????????????????????????????????????????????????????????????????????????????????????????????????????????????????????????????????????????????????????????????????????????????????????????????????????????????????????????????????????????????????????????????????????????????????????????????????????????????????????????????????????????????????????????????????????????????????????????????????????????????????????????????????????????????????????????????????????????????????????????????????????????????????????????????????????????????????????????????????????????????????????????????????????????????????????????????????????????????????????????????????????????????????????????????????????????????????????????????????????????????????????????????????????????????????????????????????????????????????????????????????????????????????????????????????????????????????????????????????????????????????????????????????????????????????????????????????????????????????????????????????????????????????????????????????????????????????????????????????????????????????????????????????????????????????????????????????????????????????

>*Pseudoechria*_*decidua*_CBS_254_71

ATTACCGAGTTCT---------TACAGAACC-CA-ACCCTGTGTGATAA-GTTCTCACCC---GAACTTTTCCACTACAG------------------GTCTCGGTCTACG----------GGCAGGCAGCGCCAG-------CCGCGGT------GGACGTCGAGACA-CCTGTAGGA-------AATCT-AAACCTC-TGTATCATTATCACAAATTTCTGAGCGACTTTACATAAAGAGTCAAAACTTTC-AACAACGGATCTCTTGGTTCTGGCATCGATGAAGAACGCAGCGAAATGCGATACGTAATGTGAATTGCAGAATTCAGTGAATCATCGAATCTTTGAACGCACATTGCGCCCGCCAGTATTCTGGCGGGCATGCCTGTCCGAGCGTCATTTCAACCCATCAAGCCCAG-CGCTT-GTGTTGGAGCCCTGCGG----CCG-CCGCAGCCTCCCAAAATTAGTGGCGGGCTCGCTAT-CACGCTGAGTGCAGTAGTATTCTTCTCACTCCTGTGGT-GTAGCGGGTAACCAGCCGTAAAAACCCCCTT----ACTTTAAAAGTTGACCTCGGATCAGGTAGGAATACCCGCTGAACTTAAGCATATCAATAAGCGGAGGAAAAGAAACCAACAGGG-ATTGCCCTAGTAACGGCGAGTGAAGCGGCAACAGCTCAAATTTGAAATCTGGCAACAG-CCCGAGTTGTAATTTGCAGAGGAAGCTTCTGGCGACGCACTGTCTAAGTCCCCTGGAACGGGGCGCCACAGCGGGTGAGAGCCCCATGT-GATGGCTGCGGACCCAGTGTGAAGCTCCTTCGACGAGTCGAGTAGTTTGGGAATGCTGCTCAAAATGGGAGGTAAATTCCTTCTAAAGCTAAATATTGGCCAGAGACCGATAGCGCACAAGTAGAGTGATC-------GAAAGATGAAAAGCACTTTGAAAAGAGGGTTAAACAGCACGTGAAATTGTTGAAAGGGAAGCGCTCATGACCAGACTTGCGCTGGGCTGATCATCCGGTGTTCTCACCGGTGCACTCTGCCCGGCTCAGGCCAGCATCGGTTTTGGCGGGGGGATAAAGACGCCGGGAACGTAGCTCCCCCGGGAGTGTTATAGCCCGGCGTGCAATACCCCCGCTGGGACCGAGGTCCGCGCATC-GCAAGGATGCTGGCGTAATGGTCATCAGCGACCCGTCTTGAAACACGGACCAAGGAGTCAAGGTTTTGCGCGAGTGTTTGGGTGTCAAACCCGCACGCGTAATGAAAGTGAA-CGTAGGTGAGAG----CTTCGGCGCATCATCGACCGATCCTGAAGTTTACGGACGGATTTGAGTAGGAGCGTTAAGCCTTGGACCCGAAAGATGGTGAACTATGCTTGGATAGGGTGAAGCCAGAGGAAACTCTGGTGGAGGCTCGCAGCGGTTCTGACGTGCAAATCGATCGTCAAATC-TGAGCATGGGGGCGAAAGACTAATCGAACCATCTAGTAGCTGG-TACCGC????????????????????????????????????????????????????????????????????????????????????????????????????????????????TCCCATTTGCGGCGAACAAATACCCCCATCGGCCGCGATGGCAAGCTGGCTAAACCCCGACAGCTGCACAACACCCACTGGGGTCTCGTGTGCCCTGCAGAGACCCCAGAAGGACAAGCCTGCGGTCTCGTCAAGAATCTCTCCTTGATGTGCTATATTAGCGTCGGCACGAACGCAGAACCCATCGTCGACTTTATGACAGCTAGGAACATGGAACTTTTGGAAGAATACGAGCCGCTACGCTATCCCAATGCCACCAAGGTCTTCGTCAACGGAACGTGGGTGGGCGTCCATCAGGATCCAAAGCACCTGGTCAACCTGGTCCAAGGTCTGAGGAGAGCGGGCGTCATTAGTTTCGAGGTTTCGCTCGTCCGAGACATCCGCGACCGAGAGTTCAAGATCTTCTCCGATGCTGGCCGTGTCATGAGGCCGCTGTTTGTTGTCGCGCAGGACGATGACAAAGAG---AAGAATGGCGGCGTCGAAAAGGGACAGCTTGTTCTGACAAGACAACACATTGACCGGCTGGCACGGGACAAGGAAATCGGTAATTTGGACGAGAACTACCTGGGTTGGAATGGACTCCTTAGAGAGGGTTGCGTCGAGTACCTGGACGCCGAGGAGGAGGAGACGACCATGATCTGCATGAGCCCCGAGGACCTGGATGCTTTCCGGCTCACCAAGCT------------TGGTCATAATCCAAACGAGGAGGAGTCTGGCGGGCCCAACAAGCGTATCAAGACGAAGGTCAACCCGACGACCCACATGTATACGCATTGCGAGATTCACCCCAGCATGCTGCTTGGTATCTGCGCCAGCATCATTCCCTTCCCCGACCACAACCAA??????????????????????????????????????????????????????????????????????????????????????????????????????????????????????????????????????????????????????????????????????????????????????????????????????????????????????????????????????????????????????????????????????????????????????????????????????????????????????????????????????????????????????????????????????????????????????????????????????????????????????????????????????????????????????????????????????????????????????????????????????????????????????????????????????????????????????????????????????????????????????????????????????????????????????????????????????????

>*Podospora*_*excentrica*_CBS_118392

ATTACCGAGTTGTAA-------TAAACTCC--CAAAACCTTTGTGATCCTTACCTA--------TTCATTTCCCCGCAGCGCCCTCCCCTCTTGAGTTTCAGGGGCTCCCC-------TCCAAGGGAGCGAACTGG-------CTTCATT-----------GGCTTGAG-CCCGGGAGAAC-----AAAAC-AACCCTTCTGTAATTTAC-TTAGCATCTCTGAGT-CTTTAGACAAATGAGTCAAAACTTTC-AACAACGGATCTCTTGGTTCTGGCATCGATGAAGAACGCAGCGAAATGCGATACGTAATGTGAATTGCAGAATTCAGTGAATCATCGAATCTTTGAACGCACATTGCGCCCGCCGGTATTCTGGCGGGCATGCCTGTCCGAGCGTCATTTCAACCCATCAAGCCCAG-CGCTT-GTGTTGGAGTCCTGCGG----CCGCCCGCAGCCTCCCAAAGTCAGTGGCGGGCTCGCCAA-CACGCTGAGTGCAGTAGTATTCTCCTCACTCTTGCTGTGTTAGCGGGT-TCCGGCCGTTAAA---CCCCCTATAAACTTACAAGGTGACCTC??????????????????????????????????????????????????AAAGAAACCAACAGGG-ATTGCCTCACCCACGGCGAGTGAAGCGGCAACAGCTCAAATTTGAAATCTGGCTCCGG-CCCGAGTTGTAATTTGCAGAGGAAGCTTCTGGAGACGCGCCGTCTAAGTCCCCTGGAACGGGGCGCCACAGCGGGTGAGAGCCCCATGT-GACGGTTGCAGACCCAGTGTGAAGCTCCTTCGACGAGTCGAGTAGTTTGGGAATGCTGCTCAAAATGGGAGGTAAATTCCTTCTAAAGCTAAATATTGGCCAGAGACCGATAGCGCACAAGTAGAGTGATC-------GAAAGATGAAAAGCACTTTGAAAAGAGGGTTAAACAGCACGTGAAATTGTTGAAAGGGAAGCGCTCATGACCAGACTTGCGCCGGGCTGATCATCTGGTGTTCTCACCAGTGCACTCCGCCCGGCTCAGGCCAGCATCGGTTTCGGCGGGGGGATAAAGGCGCTGGGAACGTAGCTCCCCCGGGAGTGTTATAGCCCAGCGTGCAATACCCCCGCTGGGACCGAGGTCCGCGCAT-TGCAAGGATGCTGGCGTAATGGTCATCAGCGACCCGTCTTGAAACACGGACCAAGGAGTCAAGGTTTTGCGCGAGTGTTTGGGTGTCAAACCCGCACGCGTAATGAAAGTGAA-CGTAGGTGAGAG----CTTCGGCGCATCATCGACCGATCCTGATGTTTACGGACGGATTTGAGTAAGAGCGTTAAGCCTTGGACCCGAAAGATGGTGAACTATGCTTGGATAGGGTGAAGCCAGAGGAAACTCTGGTGGAGGCTCGCAGCGGTTCTTACGTGCAAATCGATCGTCAAATC-CAACCACCGGGGCGAAAGACTAATCGAACCATCTAGTAGCTGGTTACCGCC????????????????????????????????????????????????????????????????????????????????????????????????????????????????????????????????????????????????????????????????????????????????????????????????????????????????????????????????????????????????????????????????????????????????????????????????????????????????????????????????????????????????????????????????????????????????????????????????????????????????????????????????????????????????????????????????????????????????????????????????????????????????????????????????????????????????????????????????????????????????????????????????????????????????????????????????????????????????????????????????????????????????????????????????????????????????????????????????????????????????????????????????????????????????????????????????????????????????????????????????????????????????????????????????????????????????????????????????????????????????????????????????????????????????????????????????????????????????????????????????????????????????????????????????????????????????????????????????????????????????????????????????????????????????????????????????????????????????????????????????????????????????????????????????????????????????????????????????????????????????????????????????????????????????????????????????????????????????????????????????????????????????????????????????????????????????????????????????????????????????????????????????????????????????????????????????????????????????????????????????????????????????????????????????????????????????????????????????????????????????????????????????????????????????????????????????????????????????

>*Podospora*_*interstinacea*_CBS_113106

ATTACAGAGCTGC---------AAAACTCC--CA-ACCCTTTGTGAACG-AACCTA--------ACAGTTGCCTCGGCGG------------------CGAGCCTTC-------------------------------------------------------AGGGCGG-ACCGCCAGAGG-----AATAC-AAACTCT----TTGCATT-TTAGCATCTCTGATT-AC-TTATTCAATAAGTTAAAACTTTC-AACAACGGATCTCTTGGTTCTGGCATCGATGAAGAACGCAGCGAAATGCGATAAGTAATGTGAATTGCAGAATTCAGTGAATCATCGAATCTTTGAACGCACATTGCGCCCGCTAGTATTCTGGCGGGCATGCCTGTTCGAGCGTCATTTCAA-CCATCAAGCCCTA-GGCTT-GCGTTGGAGCCCTGCGG----CCG-CCGCAGCCTCCCAAATACAGTGGCGGGCTCGCTAT-CACACCGAGTGCAGTAGTTTACTCTTCGCTCAGGGCGT-GTGGCGGGT-GCTAGCCGTAAAA---CCCCCC-GATATCTAAAGGTTGACCTCGGATCAGGTAGGAATACCCGCTGAACTTAAGCATATCAATAAGCGGAGGAA?????????ACAGGG-ATTGCCCTAGTAACGGCGAGTGAAGCGGCAACAGCTCAAATTTGAAATCTGGCTCCGG-CCCGAGTTGTAATTTGCAGAGGAAGCTTCTGGTGATATACCGTCTAAGTCCCCTGGAACGGGGCGCCACAGTGGGTGAGAGCCCCATGT-GACGGATGTAGATCCTGTGTGAAGCTCCTTCGACGAGTCGAGTAGTTTGGGAATGCTGCTCAAAATGGGAGGTAAATTCCTTCTAAAGCTAAATATTGGCCAGAGACCGATAGCGCACAAGTAGAGTGATC-------GAAAGATGAAAAGCACTTTGAAAAGAGGGTTAAACAGCACGTGAAATTGTTGAAAGGGAAGCGCTTGTGACCAGACTTGCGCTGGGCTGATCATCCGGTGTTCTCACCGGTGCACTCGGCCCAGCACAGGCCAGCATCGGTTTTGGCGGGGGGATAAAGACTCAGGGAATGTAGCTCCTCCGGGAGTGTTATAGCCCGGTGTGCAATACCCCCGCTGGGACCGAGGTTCGCGCATTCGCAAGGATGCTGGCGTAATGGTCATCAGCGACCCGTCTTGAAACACGGACCAAGGAGTCAAGGTTTTGCGCGAGTGTTTGGGTGTCAAACCCGCACGCGTAATGAAAGTGAA-CGTAGGTGAGAG----CTTCGGCGCATCATCGACCGATCCTGATGTATTCGGATGGATTTGAGTAGGAGCGTTAAGCCTTGGACCCGAAAGATGGTGAACTATGCTTGGATAGGGTGAAGCCAGAGGAAACTCTGGTGGAGGCTCGCAGCGGTTCTGACGTGCAAATCGATCGTCAAATC-TGAGCATGGGGGCGAAAGACTAATCGA????????????????????????????????????????????????????????????????????????????????????????????????????????????????????????????????????????????????????????????????????????????????????????????????????????????????????????????????????????????????????????????????????????????????????????????????????????????????????????????????????????????????????????????????????????????????????????????????????????????????????????????????????????????????????????????????????????????????????????????????????????????????????????????????????????????????????????????????????????????????????????????????????????????????????????????????????????????????????????????????????????????????????????????????????????????????????????????????????????????????????????????????????????????????????????????????????????????????????????????????????????????????????????????????????????????????????????????????????????????????????????????????????????????????????????????????????????????????????????????????????????????????????????????????????????????????????????????????????????????????????????????????????????????????????????????????????????????????????????????????????????????????????????????????????????????????????????????????????????????????????????????????????????????????????????????????????????????????????????????????????????????????????????????????????????????????????????????????????????????????????????????????????????????????????????????????????????????????????????????????????????????????????????????????????????????????????????????????????????????????????????????????????????????????????????????????????????????????????????????????????????

>*Pseudoechria*_*longicollis*_CBS_368_52

ATTACCGAGTTCT---------TACAGAACC-CA-ACCCTGTGTGATAA-GTTCTCACCC---GAACTTTTCCACTACAG------------------GTCTCGGTCCACG----------GGCAGGCAGCGCCGG-------CCGCGGT------GGACTTCGAGGCA-CCTGTAGGA-------AACCC-AAACCTC-TGTATCATTAACACAAATATCTGAGCGACTTTACATAAAGAGTCAAAACTTTC-AACAACGGATCTCTTGGTTCTGGCATCGATGAAGAACGCAGCGAAATGCGATACGTAATGTGAATTGCAGAATTCAGTGAATCATCGAATCTTTGAACGCACATTGCGCCCGCCAGTATTCTGGCGGGCATGCCTGTCCGAGCGTCATTTCAACCCATCAAGCCCAG-CGCTT-GTGTTGGAGCCCTGCGG----CCG-CCGCAGGCTCCCAAAATTAGTGGCGGGCTCGCTAT-CACGCTGAGTGCAGTAGTATTCTTCTCACTCCTGCGGT-GTAGCGGGTAACCAGCCGTAAAA---CCCTT----TTACCTAAAGTTGACCTCGGATCAGGTAGGAATACCCGCTGAACTTAAGCATATCAATAAGCGGAGGAAAAGAAACCAACAGGG-ATTGCCCTAGTAACGGCGAGTGAAGCGGCAACAGCTCAAATTTGAAATCTGGCAACAG-CCCGAGTTGTAATTTGCAGAGGAAGCTTCTGGCGACGCACTGTCTAAGTCCCCTGGAACGGGGCGCCACAGCGGGTGAGAGCCCCATGT-GATGGCTGCGGACCCAGTGTGAAGCTCCTTCGACGAGTCGAGTAGTTTGGGAATGCTGCTCAAAATGGGAGGTAAATTCCTTCTAAAGCTAAATATTGGCCAGAGACCGATAGCGCACAAGTAGAGTGATC-------GAAAGATGAAAAGCACTTTGAAAAGAGGGTTAAACAGCACGTGAAATTGTTGAAAGGGAAGCGCTCATGACCAGACTTGCGCTGGGCTGATCATCCGGTGTTCTCACCGGTGCACTCTGCCCGGCTCAGGCCAGCATCGGTTTTGGTGGGGGGATAAAGGCGCCGGGAACGTAGCTCCTCCGGGAGTGTTATAGCCCGGTGTGCAATACCCCCGCTGGGACCGAGGTCCGCGCATC-GCAAGGATGCTGGCGTAATGGTCATCAGCGACCCGTCTTGAAACACGGACCAAGGAGTCAAGGTTTTGCGCGAGTGTTTGGGTGTCAAACCCGCACGCGTAATGAAAGTGAA-CGTAGGTGAGAG----CTTCGGCGCATCATCGACCGATCCTGAAGTTTACGGACGGATTTGAGTAGGAGCGTTAAGCCTTGGACCCGAAAGATGGTGAACTATGCTTGGATAGGGTGAAGCCAGAGGAAACTCTGGTGGAGGCTCGCAGCGGTTCTGACGTGCAAATCGATCGTCAAATC-TGAGCATGGGGGCGAAAGACTAATCGAACCATCTAGTAGCTGGGTACCGCA????????????????????????????????????????????????????????????????????????????????????????????????????????????????????????????????????ACCCCCATCGGCCGCGATGGCAAGCTGGCTAAACCCCGACAGCTGCACAACACCCACTGGGGTCTCGTGTGCCCTGCAGAGACCCCAGAAGGACAAGCTTGCGGTCTCGTCAAAAATCTTTCCTTAATGTGCTACATTAGTGTCGGCACGAACGCAGAACCCATCGTCGACTTCATGACAGCTAGGAACATGGAACTTTTGGAGGAATATGAGCCGCTACGCTATCCCAATGCCACCAAGGTCTTCGTCAACGGAACGTGGGTGGGCGTCCATCAGGACCCCAAGCATCTGGTCACCCTGGTCCAAGGTCTGAGGAGAGCTGGCGTCATTAGTTTCGAGGTTTCGCTCGTCCGAGACATCCGCGACCGAGAGTTCAAGATCTTCTCCGATGCTGGCCGTGTCATGAGGCCGCTGTTTGTTGTCGCGCAGGACGATGACAAAGAG---AAGAATGGCGGCGTGGAAAAGGGACAGCTTGTTCTCACAAGACAACACATTGACCGGCTGGCACGGGACAAGGAAATCGGAAATTTGGACGAGAACTACCTGGGCTGGAATGGTCTTCTGAGAGAGGGTTGCGTCGAGTATCTGGACGCCGAGGAAGAGGAGACGACCATGATCTGCATGAGCCCCGAGGACCTGGATGCTTTCCGGCTCACCAAGCT------------TGGTCATAATCCAAACGACGACGAGTCCGGCGGACCTAACAAGCGTATCAAGACGAAGGTGAACCCGACGACTCACATGTATACGCATTGCGAGATTCACCCCAGCATGCTGCTTGGTATCTGCGCCAGCATCATTCCCTTCCCCGACCACAACCAA??????????????????????????????????????????????????????????????????????????????????????????????????????????????????????????????????????????????????????????????????????????????????????????????????????????????????????????????????????????????????????????????????????????????????????????????????????????????????????????????????????????????????????????????????????????????????????????????????????????????????????????????????????????????????????????????????????????????????????????????????????????????????????????????????????????????????????????????????????????????????????????????????????????????????????????????????????????

>*Podospora*_*prethopodalis*_CBS_121128

ATTACTGAGTTCCAC-------TGGACTCC--CA--CCCTGTGTGATTC-GACTCGCGAACCACTTTGTTGCCTCGGCAG------------------AAAACAGTGGTTG------------------AGACCTG-------CCCCCTAGCGGTGCGGGCTCCCATCA-GCTGCCGAGCG-----AGCAA-AAACTCTTCTTGTAATT-GTTGGCATCTCTGAGT-AGCTTTTGCAATAAGTCAAAACTTTC-AACAACGGATCTCTTGGTTCTGGCATCGATGAAGAACGCAGCGAAATGCGATAAGTAATGTGAATTGCAGAATTCAGTGAATCATCGAATCTTTGAACGCACATTGCGCCCGCCAGTATTCTGGCGGGCATGCCTGCCCGAGCGTCATTTCAACCCTTCAAGCCC---AGCTT-GTGTTGGAGCCCTGCGGTCCCCCC-TCGCAGGCTTCTAAAAGCAGTGGCGGGCTCGCCAT-CACACTGAGTGCAGTAGTGT---TCTCGCTCCTGTGTG-TTGGTGGGT-TCCAGCCGTAAAG---CCCCCTACATTATACAAGGT?????????????????????????????????????????????????????????AAAGAAACCAACTGGG-ATTGCCCTAGTAACGGCGAGTGAAGCGGCAACAGCTCAAATTTGAAATCTGGCTCCGG-CCCGAGTTGTAATTTGCAGAGGAAGCTTCTGGCGACGCGCCGCCTAAGTCCCCTGGAATGGGGCGCCACAGCGGGTGAGAGCCCCGTGT-GACGGCTGCTGAACCAGTGTGAAGCTCCTTCGACGAGTCGAGTAGTTTGAGAATGCTGCTCAAAATGGGAGGTAAATTCCTTCTAAAGCTAAATACCGGCCAGAGACCGATAGCGCACAAGTAGAGTGATC-------GAAAGATGAAAAGCACTTTGAAAAGAGGGTTAAACAGCACGTGAAATTGTTGAAAGGGAAGCGCTCATGACCAGACTTGCGCTAGGCTGATCATCCGGTGTTCTCACCGGTGCACTCTGCCTAGCGCAGGCCAGCATCGGTTTCAGCGGGGGGATAAAGGCGTTGGGAACGTAGCTCCCCCGGGAGTGTTATAGCCCAGCGTGCAATGCCCCCGCCGGGACCGAGGACAGCGC-CCTGCGAGGATGCTGGCGTAATGGTCATCAGCGACCCGTCTTGAAACACGGACCAAGGAGTCAAGGTTTTGCGCGAGTGTTTGGGTGTCAAACCCGCACGCGTAATGAAAGTGAA-CGTAGGTGAGAG----CTTCGGCGCATCATCGACCGATCCTGATGTTTTCGGATGGATTTGAGTAGGAGCGTTAAGCCTTGGACCCGAAAGATGGTGAACTATGCTTGGATAGGGTGAAGCCAGAGGAAACTCTGGTGGAGGCTCGCAGCGGTTCTGACGTGCAAATCGATCGTCAAATC-TGAGCATGGGGGCG?????????????????????????????????????????????????????????????????????????????????????????????????????????????????????????????????????????????????????????????????????????????????????????????????????????????????????????????????????????????????????????????????????????????????????????????????????????????????????????????????????????????????????????????????????????????????????????????????????????????????????????????????????????????????????????????????????????????????????????????????????????????????????????????????????????????????????????????????????????????????????????????????????????????????????????????????????????????????????????????????????????????????????????????????????????????????????????????????????????????????????????????????????????????????????????????????????????????????????????????????????????????????????????????????????????????????????????????????????????????????????????????????????????????????????????????????????????????????????????????????????????????????????????????????????????????????????????????????????????????????????????????????????????????????????????????????????????????????????????????????????????????????????????????????????????????????????????????????????????????????????????????????????????????????????????????????????????????????????????????????????????????????????????????????????????????????????????????????????????????????????????????????????????????????????????????????????????????????????????????????????????????????????????????????????????????????????????????????????????????????????????????????????????????????????????????????????????????????????????????????????????????????????

>*Zopfiella*_*erostrata*_CBS_255_71

ATTACAGAGTTGC---------AAAACTCC--CA-ACCCTTTGTGAATC-AACTAG--------ACAGTTGCCTCGGCGG------------------GAGGCCCTTCAG-----------------------------------------------------GGGCTG-CCCGCCGGAGG-----ACCACTAAACTCT----TGTATTT-TTGGCCTCTCTGAGT-AA-TTCTAAAATGAATCAAAACTTTC-AACAACGGATCTCTTGGTTCTGGCATCGATGAAGAACGCAGCGAAATGCGATACGTAATGTGAATTGCAGAATTCAGTGAATCATCGAATCTTTGAACGCACATTGCGCCCGCCAGCACTCTGGCGGGCATGCCTGTTCGAGCGTCATTTCAA-CCATCAAGCCCCA-GGCTT-GTGTTGGAGCCCTGCGG----CTG-CCGCAGCCTCCTAAAAGCAGTGGCGGGCTCGCTAT-CACACCGAGTGCAGTAGATTTCTCTTCGCTCAGGACGT-GTGGCGGGT-TCCGGCCGTGAAA---CCCCC--TACTCTTTAAGGTTGACCTCGGATCAGGTAGGAATACCCGCTGAACTTAAGCATATCAATAAGCGGAGGAA???????CAACAGGG-ATTGCCCTAGTAACGGCGAGTGAAGCGGCAACAGCTCAAATTTGAAATCTGGCTCCGG-CCCGAGTTGTAATTTGCAGAGGAAGCTTCTGGTGATGCACTGTCTAAGTCCCCTGGAACGGGGCGCCACAGTGGGTGAGAGCCCCATTT-GACAGAGGCAGATCCTGTGTGAAGCTCCTTCGACGAGTCGAGTAGTTTGGGAATGCTGCTCAAAATGGGAGGTAAATTCCTTCTAAAGCTAAATATTGGCCAGAGACCGATAGCGCACAAGTAGAGTGATC-------GAAAGATGAAAAGCACTTTGAAAAGAGGGTTAAACAGCACGTGAAATTGTTGAAAGGGAAGCGCTTGTGACCAGACTTGCGCTGGGCTGATCATCCGGTGTTCTCACCGGTGCACTCGGCCCAGCTCAGGCCAGCATCGGTTTTGGTGGGGGGATAAAGGCGCTGGGAACGTAGCTCCTTCGGGAGTGTTATAGCCCAGCGTGCAATGCCCCCGCTGGGACCGAGGTTCGCGCATCTGCAAGGATGCTGGCGTAATGGTCATCAGCGACCCGTCTTGAAACACGGACCAAGGAGTCAAGGTTTTGCGCGAGTGTTTGGGTGTCAAACCCGCACGCGTAATGAAAGTGAA-CGTAGGTGAGAG----CTTCGGCGCATCATCGACCGATCCTGATGTATTCGGATGGATTTGAGTAGGAGCGTTAAGCCTTGGACCCGAAAGATGGTGAACTATGCTTGGATAGGGTGAAGCCAGAGGAAACTCTGGTGGAGGCTCGCAGCGGTTCTGACGTGCAAATCGATCGTCAAATC-TGAGCATGGGGGCGAAAGACTAATCGA????????????????????????????????????????????????????????????????????????????????????????????????????????????????????????????????????????????????????????????????????????????????????????????????????????????????????????????????????????????????????????????????????????????????????????????????????????????????????????????????????????????????????????????????????????????????????????????????????????????????????????????????????????????????????????????????????????????????????????????????????????????????????????????????????????????????????????????????????????????????????????????????????????????????????????????????????????????????????????????????????????????????????????????????????????????????????????????????????????????????????????????????????????????????????????????????????????????????????????????????????????????????????????????????????????????????????????????????????????????????????????????????????????????????????????????????????????????????????????????????????????????????????????????????????????????????????????????????????????????????????????????????????????????????????????????????????????????????????????????????????????????????????????????????????????????????????????????????????????????????????????????????????????????????????????????????????????????????????????????????????????????????????????????????????????????????????????????????????????????????????????????????????????????????????????????????????????????????????????????????????????????????????????????????????????????????????????????????????????????????????????????????????????????????????????????????????????????????????????????????????????

>*Pseudoechria*_*prolifica*_CBS_250_71

ATTACCGAGTTCT---------TACAGAACC-CA-ACCCTGTGTGATAA-GTTCTCACCC---GAACTTTTCCACTACAG------------------GTCTCGGTCCG----------------------------------CCGCGGT------GGACTTCGAGGCG-CCTGTAGGA-------AACCC-AAACCTC-TGTATCATTAACACAAATATCTGAGCGACTTTACATAAAGAGTCAAAACTTTC-AACAACGGATCTCTTGGTTCTGGCATCGATGAAGAACGCAGCGAAATGCGATACGTAATGTGAATTGCAGAATTCAGTGAATCATCGAATCTTTGAACGCACATTGCGCCCGCCAGTATTCTGGCGGGCATGCCTGTCCGAGCGTCATTTCAACCCATCAAGCCCAG-CGCTT-GTGTTGGAGCCCTGCGG----CCG-CCGCAGCCTCCCAAAATTAGTGGCGGGCTCGCTAT-CACGCTGAGTGCAGTAGTATTCTTCTCACTCCTGCGGT-GTAGCGGGTTACCAGCCGTAAAA---CCCTT----TTACCTAAAGTTGACCTCGGATCAGGTAGGAATACCCGCTGAACTTAAGCATATCAATAAGCGGAGGAAAAGAAACCAACAGGG-ATTGCCCTAGTAACGGCGAGTGAAGCGGCAACAGCTCAAATTTGAAATCTGGCAACAG-CCCGAGTTGTAATTTGCAGAGGAAGCTTCTGGCGACGCACTGTCTAAGTCCCCTGGAACGGGGCGCCACAGCGGGTGAGAGCCCCATGT-GATGGCTGCGGACCCAGTGTGAAGCTCCTTCGACGAGTCGAGTAGTTTGGGAATGCTGCTCAAAATGGGAGGTAAATTCCTTCTAAAGCTAAATATTGGCCAGAGACCGATAGCGCACAAGTAGAGTGATC-------GAAAGATGAAAAGCACTTTGAAAAGAGGGTTAAACAGCACGTGAAATTGTTGAAAGGGAAGCGCTCATGACCAGACTTGCGCTGGGCTGATCATCCGGTGTTCTCACCGGTGCACTCTGCCCGGCTCAGGCCAGCATCGGTTTTGGCGGGGGGATAAAGGCGCCGGGAACGTAGCTCCTCCGGGAGTGTTATAGCCCGGTGTGCAATACCCCCGCTGGGACCGAGGTCCGCGCATC-GCAAGGATGCTGGCGTAATGGTCATCAGCGACCCGTCTTGAAACACGGACCAAGGAGTCAAGGTTTTGCGCGAGTGTTTGGGTGTCAAACCCGCACGCGTAATGAAAGTGAA-CGTAGGTGAGAG----CTTCGGCGCATCATCGACCGATCCTGAAGTTTACGGACGGATTTGAGTAGGAGCGTTAAGCCTTGGACCCGAAAGATGGTGAACTATGCTTGGATAGGGTGAAGCCAGAGGAAACTCTGGTGGAGGCTCGCAGCGGTTCTGACGTGCAAATCGATCGTCAAATC-TGAGCATGGGGGCGAAAGACTAATCGAACCATCTAGTAGCTGGTTACCGCG?????????????????????????????????????????????????????????????????????????????????????????????????????????ACACTGTCGCATTTGCGGCGAACAAATACCCCCATCGGCCGCGATGGCAAGCTGGCTAAACCCCGACAGCTGCACAACACCCACTGGGGTCTCGTGTGCCCTGCAGAGACCCCGGAAGGACAAGCTTGCGGTCTCGTCAAAAATCTTTCCTTAATGTGCTACATTAGTGTCGGCACGAACGCAGAACCCATCGTCGACTTCATGACAGCTAGGAACATGGAACTTCTGGAGGAATACGAGCCGCTGCGCTATCCCAATGCCACCAAGGTCTTCGTCAACGGAACGTGGGTGGGCGTCCATCAGGACCCCAAGCATCTGGTCACCCTGGTCCAAGGTTTGAGGAGAGCTGGCGTCATTAGTTTCGAGGTTTCGCTCGTCCGAGACATCCGCGACCGAGAGTTCAAGATCTTTTCCGATGCCGGCCGTGTCATGAGGCCGCTGTTTGTTGTCGCGCAGGACGACGACAAAGAG---AAGAATGGCGGCGTCGAAAAGGGACAGCTTGTTCTCACAAGACAACACATTGACCGGCTGGCACGGGACAAGGAAATCGGGAATTTGGACGAGAACTACCTGGGCTGGAATGGTCTTCTGAGAGAGGGTTGCGTCGAGTATCTGGACGCCGAGGAAGAGGAGACGACCATGATCTGCATGAGCCCCGAGGACCTGGATGCTTTCCGGCTCACCAAGCT------------TGGTCATAATCCAAACGACGACGAGTCCGGCGGACCCAACAAGCGTATCAAGACGAAGGTCAACCCGACGACTCACATGTATACGCATTGCGAGATTCACCCCAGCATGCTGCTTGGCATCTGCGCCAGCATCATTCCCTTCCCCGACCACAACCAA??????????????????????????????????????????????????????????????????????????????????????????????????????????????????????????????????????????????????????????????????????????????????????????????????????????????????????????????????????????????????????????????????????????????????????????????????????????????????????????????????????????????????????????????????????????????????????????????????????????????????????????????????????????????????????????????????????????????????????????????????????????????????????????????????????????????????????????????????????????????????????????????????????????????????????????????????????????

>*Garciamycella*_*cyclaminis*_CBS_120402

ATTAGCGAGTAT----------AACTCTCT--AA-AACCATTGTGAACG-AACAGAC--------CGGTTGCTTCGGCGG------------------ACGGCTT---------------------GCCG-----------------------------------------CCGCCGGAAG-----AACTC-AAACTCT----TGTTATC-ATTGTCTCTCTGAGT-AAATTACTTAATAAGTTAAAACTTTC-AACAACGGATCTCTTGGTTCTGGCATCGATGAAGAACGCAGCGAAATGCGATAAGTAATGTGAATTGCAGAATTCAGTGAATCATCGAATCTTTGAACGCACATTGCGCCCGCCAGTATTCTGGCGGGCATGCCTGTTCGAGCGTCATTTCAA-CCATCAAGCCCCA-GGCTT-GTGTTGGGGGCCTGCAG----CTG-CTGCAGCCCCCTAAAAGCAGTGGCGGACTCGCTGT-CATTCCGAGCGCAGTAG-TTACATCTCGCTCTGGACGTGGCGGCGGGC-ACCTGCCGTGAAA---CACTC-----TTTCTAAGGTTGACCTCGGATCAGGTAGGAATACCCGCTGAACTTAAGCATATCAATAAGCGGAGGAAAAGAAACCAACAGGG-ATTGCCCCAGTAACGGCGAGTGAAGCGGCAACAGCTCAAATTTGAAATCTGGCTTCGG-CCCGAGTTGTAATTTGTAGAGGAAGCTTCTGGCGCGGTGCCATCCGAGTCCCCTGGAACGGGGCGCCATAGAGGGTGAGAGCCCCGTATGGATGGATGCCTAGCCTGTGTGAAGCTCCTTCGACGAGTCGAGTAGTTTGGGAATGCTGCTCAAAATGGGAGGTAAATTCCTTCTAAAGCTAAATATTGGCCAGAGACCGATAGCGCACAAGTAGAGTGATC-------GAAAGATGAAAAGCACTTTGAAAAGAGGGTTAAACAGCACGTGAAATTGTTGAAAGGGAAGCGCTTGTGACCAGACTTGCGCCAGGCTGATCATCCGGTGTTCTCACCGGTGCACTCTGCCTGGCACAGGCCAGCATCAGTTTTGGTGGGGGGATAAAGGGCGCAGGAACGTAGCTCCTCCGGGAGTGTTATAGCCTGTGTCGTAATGCCCTCGCCGGGACTGAGGACCGCGCATCTGCAAGGATGCTGGCGTAATGGTTATCAGCGACCCGTCTTGAAACACGGACCAAGGAGTCAAGGTTTTGCGCGAGTGTTTGGGTGTAAAACCCGCACGCGTAATGAAAGTGAA-CGTAGGTGAGAG----CTTCGGCGCATCATCGACCGATCCTGATGTATTCGGATGGATTTGAGTAAGAGCGTTAAGCCTTGGACCCGAAAGATGGTGAACTATGCTTGGATAGGGTGAAGCCAGAGGAAACTCTGGTGGAGGCTCGCAGCGGTTCTGACGTGCAAATCGATCGTCAAATC-TGAGCATGGGGGCGAAAGACTAATCGAACCATCTAGTAGCTGGTTACCGCC???CTCAAGTACTCTTTGGCGACRGGCAATTGGGGCGACCAGAAGAAGGCAATGAGTTCGACGGCTGGTGTGTCGCAGGTGTTGAACAGATACACCTTCGCGTCTACCCTGTCCCATTTGCGTCGCACCAACACCCCGATCGGTCGTGATGGCAAACTCGCAAAGCCTCGTCAGCTCCACAACACCSATTGGGGCTTGGTTTGCCCTGCCGAAACACCCGAGGGGCAGGCTTGCGGTCTCGTCAAGAACCTGTCCTTGATGTGTTACATCAGTGTCGGTACCAACGCGGAGCCGATCGTTGACTTTATGATTGCAAGAAACATGGAGGTGCTGGAAGAATACGAGCCGCTCCGGTATCCCAACGCCACCAAGGTCTTTGTGAATGGTACCTGGGTCGGTGTTGTTCAGGACGCCAAAAGTCTGGTTGGTCTTGTCCAGGGGCTGAGACGATCAAATATTATTTCCTTCGAGGTGTCCCTGGTCAGAGATATCCGAGACCGCGAGTTCAAGATCTTTTCAGATGCCGGTCGCGTCATGAGGCCTCTCTTCGTTGTCGAGCAAGAAGATGGCAACCCG------AACACCAAGGTTGAGCGAGGACAGCTGGTGTTGACGAAGGACCACACGGCGAAGTTGGAGCACGACAGGGAGATTGGGAGGTTTGACCCCAATTATTACGGATGGGATGGCCTGCTCCGGGACGGATGTGTCGAGTATCTCGATGCAGAGGAAGAGGAGACAACCATGATCTGCATGTCGCCTGAAGATCTCGAACACTTCCGAACGACAAAGAT?????????????????????????????????????????????????????????????????????????????????????????????????????????????????????????????????????????????????????????????????????????GCTTGTTGACCAAGTTCTCGATGTTGTTCGTCGCGAGGCTGAGGGCTGCGACTGCCTTCAGGGCTTCCAGATCACCCACTCTCTCGGTGGTGGTACTGGTGCCGGTATGGGTACCCTCCTTATCTCCAAGATTCGTGAGGAGTTCCCCGACCGCATGATGGCGACCTTCTCCGTCGTCCCCTCTCCCAAGGTCTCGGATACCGTTGTCGAGCCCTACAACGCCACCCTCTCCGTCCACCAGCTTGTTGAGAACTCCGACGAGACCTTCTGCATTGACAACGAGGCTCTCTACGACATCTGCATGAGGACACTCAAGCTCTCCAACCCCTCGTACGGCGATCTTAACCACCTCGTTTCCGCCGTCATGTCCGGTGTTACCGTCTCGCTCCGTTTCCCCGGCCAGCTCAACTCGGATCTCCGCAAGCTTGCCGTGAACATGGTGCCTTTCCCCCGTCTGCATTTCTTCATGGTCGGCTTTGCGCCCCTTACCAGCCGCGGCGCCCACTCTTTCCGTGCCGTCTCGGTTCCCGAGCTCACGCAGCAGATGTTCGACCCCAAGAACATGATGGCTGCTTCCGACTTCCGTAACGGTCGCTACCTCACTTGCTCCGCCATCTT

>*Garciamycella*_*chlamydospora*_CBS_150388

ATTAGCGAGTAT----------AACTCTCT--AA-AACCATTGTGAACG-AACGAAC--------CCGTTGCTTCGGCGG------------------ACGGCTCT--------------------GCCG-----------------------------------------CCGCCGGAAG-----AACGC-AAACTCT----TGTTATT-ATTGTTTCTCTGAGT--ACTTATTCAATAAGTAAAAACTTTC-AACAACGGATCTCTTGGTTCTGGCATCGATGAAGAACGCAGCGAAATGCGATAAGTAATGTGAATTGCAGAATTCAGTGAATCATCGAATCTTTGAACGCACATTGCGCCCGCCAGTATTCTGGCGGGCATGCCTGTTCGAGCGTCATTTCAA-CCATCAAGCCCCA-GGCTT-GTGTTGGGGGCCTGCAG----CTG-CTGCAGCCCCCTAAAAGCAGTGGCGGACTCGCTGT-CATTCCGAGCGCAGTAG-TTACATCTCGCTCTGGGCGTGGCGGTGGGC-ACCTGCCGTGAAA---CACTC-----TTTCTAAGGTTGACCTCGGATCAGGTAGGAATACCCGCTGAACTTAAGCATATCAATAAGCGGAGGAAAAGAAACCAACAGGG-ATTGCCCTAGTAACGGCGAGTGAAGCGGCAACAGCTCAAATTTGAAATCTGGCTTCGG-CCCGAGTTGTAATTTGTAGAGGAAGCTTCTGGCGCGGTGCCATCCGAGTCCCCTGGAACGGGGCGCCATAGAGGGTGAGAGCCCCGTATGGATGGATGCCTAGCCTGTGTGAAGCTCCTTCGACGAGTCGAGTAGTTTGGGAATGCTGCTCAAAATGGGAGGTAAATTCCTTCTAAAGCTAAATATTGGCCAGAGACCGATAGCGCACAAGTAGAGTGATC-------GAAAGATGAAAAGCACTTTGAAAAGAGGGTTAAACAGCACGTGAAATTGTTGAAAGGGAAGCGCTTGTGACCAGACTTGCGCCAGGCTGATCATCCGGTGTTCTCACCGGTGCACTCTGCCTGGCACAGGCCAGCATCAGTTTTGGTGGGGGGATAAAGGGCGCAGGAACGTAGCTCCTCCGGGAGTGTTATAGCCTGTGTCGTAATGCCCTCGCCGGGACTGAGGACCGCGCATCTGCAAGGATGCTGGCGTAATGGTCATCAGCGACCCGTCTTGAAACACGGACCAAGGAGTCAAGGTTTTGCGCGAGTGTTTGGGTGTAAAACCCGCACGCGTAATGAAAGTGAA-CGTAGGTGAGAG----CTTCGGCGCATCATCGACCGATCCTGATGTATTCGGATGGATTTGAGTAAGAGCGTTAAGCCTTGGACCCGAAAGATGGTGAACTATGCTTGGATAGGGTGAAGCCAGAGGAAACTCTGGTGGAGGCTCGCAGCGGTTCTGACGTGCAAATCGATCGTCAAATC-TGAGCATGGGGGCGAAAGACTAATCGAACCATCTAGTAGCTGGTTACCGCC?????????????????????????????????????????????????????????????????????????????????????????????????TTGCGTCGACACTGTCCCATTTGCGTCGTACCAACACTCCGATCGGCCGCGATGGCAAGCTTGCGAAGCCCCGTCAGCTCCACAACACTCATTGGGGCTTGGTTTGCCCTGCCGAAACGCCCGAGGGTCAGGCTTGCGGTCTCGTCAAGAACTTGTCGTTGATGTGCTACATCAGCGTTGGCACCAATGCGGAGCCGATTGTTGACTTTATGATAGCAAGGAACATGGAAGTTCTGGAGGAGTACGAACCGCTTCGATATCCCAACGCCACCAAGGTCTTTGTGAATGGCACTTGGGTCGGTGTTGTTCAGGACGCCAAAAGCCTGGTTGGTCTTGTCCAGGGCCTGCGACGGTCGAATATTATTTCGTTCGAGGTTTCACTGGTCAGAGATATCCGAGACCGAGAGTTCAAGATCTTTTCGGATGCCGGTCGTGTCATGCGACCCCTGTTTGTTGTGGAGCAAGAAGACGGCAACACA------ACGAGCGGCGTCGAGCGAGGACAGTTGGTTTTGAAGAAGGAGCACTGCAATAAGTTGGAGCGCGATCGAGAGATTGGTAGATTCGACCCCGATTACTACGGCTGGGACGGCCTGCTCCGGGACGGATGTGTCGAGTATCTCGATGCGGAGGAAGAAGAGACAACCATGATCTGCATGTCTCCCGAAGATCTCGAGATCTTCAGAACGACGAAGAT------CTTGGGCATTCCCCAGGTCGATGTCGAGGACCCTAGTGCGGGTAACAAGCGCATCCCGACAAAACTCAATCCAACTACCCACGCGTACACGCACTGCGAAATTCACCCGAGCATGCTGCTCGGCATCTGCGCAAGCATCATTCCTTTCCCTGATCACAACCAAGCTTGTTGACCAAGTTCTCGATGTCGTCCGTCGTGAGGCTGAGGGCTGCGACTGCCTCCAGGGCTTCCAGATCACCCACTCTCTCGGTGGTGGTACCGGTGCCGGTATGGGTACCCTCCTCATCTCCAAGATCCGCGAGGAGTTCCCCGACCGCATGATGGCGACTTTCTCCGTCGTCCCCTCCCCTAAGGTGTCGGATACCGTTGTCGAGCCCTACAACGCCACCCTCTCCGTCCACCAGCTTGTTGAGAACTCGGACGAGACCTTCTGCATCGACAACGAGGCTCTCTACGACATCTGCATGAGGACACTCAAGCTCTCCAACCCCTCATACGGCGACCTTAACCACCTCGTTTCGGCCGTCATGTCCGGTGTTACCGTCTCGCTCCGTTTCCCCGGTCAGCTCAACTCGGATCTCCGCAAGCTCGCCGTGAACATGGTGCCCTTCCCCCGTCTGCACTTCTTCATGGTCGGCTTTGCCCCTCTTACTAGCCGCGGCGCCCACTCTTTCCGTGCTGTCTCGGTTCCCGAGCTCACCCAGCAGATGTTCGACCCCAAGAACATGATGGCTGCCTCCGACTTCCGCAACGGTCGTTACCTCACTTGCTCCGCCATCTT

>*Garciamycella*_*cyclaminis*_CBS_166.42

ATTAGCGAGTAT----------AACTCTCT--AA-AACCATTGTGAACG-AACAGAC--------CGGTTGCTTCGGCGG------------------ACGGCTT---------------------GCCG-----------------------------------------CCGCCGGAAG-----AACTC-AAACTCT----TGTTATC-ATTGTCTCTCTGAGT-AAATTACTTAATAAGTTAAAACTTTC-AACAACGGATCTCTTGGTTCTGGCATCGATGAAGAACGCAGCGAAATGCGATAAGTAATGTGAATTGCAGAATTCAGTGAATCATCGAATCTTTGAACGCACATTGCGCCCGCCAGTATTCTGGCGGGCATGCCTGTTCGAGCGTCATTTCAA-CCATCAAGCCCCA-GGCTT-GTGTTGGGGGCCTGCAG----CTG-CTGCAGCCCCCTAAAAGCAGTGGCGGACTCGCTGT-CATTCCGAGCGCAGTAG-TTACATCTCGCTCTGGACGTGGCGGCGGGC-ACCTGCCGTGAAA---CACTC-----TTTCTAAGGTTGACCTCGGAT??????????????????????????GCATATCAATAAGCGGAGGAAAAGAAACCAACAGGGATTGCCCCCAGTAACGGCGAGTGAAGCGGCAACAGCTCAAATTTGAAATCTGGCTTCGG-CCCGAGTTGTAATTTGTAGAGGAAGCTTCTGGCGCGGTGCCATCCGAGTCCCCTGGAACGGGGCGCCATAGAGGGTGAGAGCCCCGTATGGATGGATGCCTAGCCTGTGTGAAGCTCCTTCGACGAGTCGAGTAGTTTGGGAATGCTGCTCAAAATGGGAGGTAAATTCCTTCTAAAGCTAAATATTGGCCAGAGACCGATAGCGCACAAGTAGAGTGATC-------GAAAGATGAAAAGCACTTTGAAAAGAGGGTTAAACAGCACGTGAAATTGTTGAAAGGGAAGCGCTTGTGACCAGACTTGCGCCAGGCTGATCATCCGGTGTTCTCACCGGTGCACTCTGCCTGGCACAGGCCAGCATCAGTTTTGGTGGGGGGATAAAGGGCGCAGGAACGTAGCTCCTCCGGGAGTGTTATAGCCTGTGTCGTAATGCCCTCGCCGGGACTGAGGACCGCGCATCTGCAAGGATGCTGGCGTAATGGTTATCAGCGACCCGTCTTGAAACACGGACCAAGGAGTCAAGGTTTTGCGCGAGTGTTTGGGTGTAAAACCCGCACGCGTAATGAAAGTGAA-CGTAGGTGAGAG----CTTCGGCGCATCATCGACCGATCCTGATGTATTCGGATGGATTTGAGTAAGAGCGTTAAGCCTTGGACCCGAAAGATGGTGAACTATGCTTGGATAGGGTGAAGCCAGAGGAAACTCTGGTGGAGGCTCGCAGCGGTTCTGACGTGCAAATCGATCGTCAAATC-TGAGCATGGGGGCGAAAGACTAATCGAACCATCTAGTAGCTGGTTACCGCC?????????????????????????????????????????????????????????????????????????????????????????????????????????????????????????????????????????????GGTCGTGATGGCAAACTCGCAAAGCCTCGTCAGCTCCACAACACCCATTGGGGCTTGGTTTGCCCTGCCGAAACACCCGAGGGGCAGGCTTGCGGTCTCGTCAAGAACCTGTCCTTGATGTGTTACATCAGTGTCGGTACCAACGCGGAGCCGATCGTTGACTTTATGATTGCAAGAAACATGGAGGTGCTGGAAGAATACGAGCCGCTCCGGTATCCCAACGCCACCAAGGTCTTTGTGAATGGTACCTGGGTCGGTGTTGTTCAGGACGCCAAAAGTCTGGTTGGTCTTGTCCAGGGGCTGAGACGATCAAATATTATTTCCTTCGAGGTGTCCCTGGTCAGAGATATCCGAGACCGCGAGTTCAAGATCTTTTCAGATGCCGGTCGCGTCATGAGGCCTCTCTTCGTTGTCGAGCAAGAAGATGGCAACCCG------AACACCAAGGTTGAGCGAGGACAGCTGGTTTTGACCAAGGAACACACGGCGAAGTTGGAGCACGACAGGGAGATTGGGAGGTTTGACCCCAATTATTACGGATGGGATGGCCTGCTCCGGGACGGATGTGTCGAGTATCTCGATGCAGAGGAAGAGGAGACAACCATGATCTGCATGTCACCTGAAGATCTCGAACACTTCCGAACGAC?????????????????????????????????????????????????????????????????????????????????????????????????????????????????????????????????????????????????????????????????????????????????????????????????????????????????????????????????????????????????????????????????????????????????????????????????????????????????????????????????????????????????????????????????????????????????????????????????????????????????????????????????????????????????????????????????????????????????????????????????????????????????????????????????????????????????????????????????????????????????????????????????????????????????????????????????????????????????????????????????????????????????????????????????????????????????????????????????????????????????????????????????????????????????????????????????????????????????????????

>*Garciamycella*_*fici*

ATTAGCGAGTAT----------AACTCTCT--AA-AACCATTGTGAACG-AACGAAC--------CCGTTGCTTCGGCGG------------------ACGGCTTT--------------------GCCG-----------------------------------------CCGCCGGAAG-----AACGT-AAACTCT----TGTTATT-ATTGTTTCTCTGAGT--ACTTATTCAATAAGTAAAAACTTTC-AACAACGGATCTCTTGGTTCTGGCATCGATGAAGAACGCAGCGAAATGCGATAAGTAATGTGAATTGCAGAATTCAGTGAATCATCGAATCTTTGAACGCACATTGCGCCCGCCAGTATTCTGGCGGGCATGCCTGTTCGAGCGTCATTTCAA-CCATCAAGCCCTA-GGCTT-GTGTTGGGGACCTGCAG----CTG-CTGCAGCCCCCTAAAAGCAGTGGCGGACTCGCTGT-CATTCCGAGCGCAGTAG-TTACATCTCGCTCTGGGTATGGCGGCGTGC-ACTTGCCGTGAAA---CACTT-------TCTAAGGTTGACCTCGGATCAGGTAGGAATACCCGCTGAACTTAAGCATATCAATAAGCGGAGGAAAAGAAACCAACAGGG-ATTGCCCTAGTAACGGCGAGTGAAGCGGCAACAGCTCAAATTTGAAATCTGGCTTCGG-CCCGAGTTGTAATTTGTAGAGGAAGCTTCTGGCGCGGTGCCATCCGAGTCCCCTGGAACGGGGCGCCATAGAGGGTGAGAGCCCCGTATGGATGGATGCCTAGCCTGTGTGAAGCTCCTTCGACGAGTCGAGTAGTTTGGGAATGCTGCTCAAAATGGGAGGTAAATTCCTTCTAAAGCTAAATATTGGCCAGAGACCGATAGCGCACAAGTAGAGTGATC-------GAAAGATGAAAAGCACTTTGAAAAGAGGGTTAAACAGCACGTGAAATTGTTGAAAGGGAAGCGCTTGTGACCAGACTTGCGCCAGGTTGATCATCCGGTGTTCTCACCGGTGCACTCTGCCTGGCACAGGCCAGCATCAGTTTTGGCGGGGGGATAAAGGGCGCAGGAACGTAGCTCCTCCGGGAGTGTTATAGCCTGTGTCGTAATGCCCTCGCCGGGACTGAGGACCGCGCATCTGCAAGGATGCTGGCGTAATGGTCATCAGCGACCCGTCTTGAAACACGGACCAAGGAGTCAAGGTTTTGCGCGAGTGTTTGGGTGTAAAACCCGCACGCGTAATGAAAGTGAA-CGTAGGTGAGAG----CTTCGGCGCATCATCGACCGATCCTGATGTATTCGGATGGATTTGAGTAAGAGCGTTAAGCCTTGGACCCGAAAGATGGTGAACTATGCTTGGATAGGGTGAAGCCAGAGGAAACTCTGGTGGAGGCTCGCAGCGGTTCTGACGTGCAAATCGATCGTCAAATC-TGAGCATGGGGGCGAAAGACTAATCGAA???????????????????????????????????????????????????????????????????????????????????????????????????????????????????????????????????????????????????????????????????????????????????????????????????????????????????????????????????????????????????????????????????????????????????????????????????????????????????????????????????????????????????????????????????????????????????????????????????????????????????????????????????????????????????????????????????????????????????????????????????????????????????????????????????????????????????????????????????????????????????????????????????????????????????????????????????????????????????????????????????????????????????????????????????????????????????????????????????????????????????????????????????????????????????????????????????????????????????????????????????????????????????????????????????????????????????????????????????????????????????????????????????????????????????????????????????????????????????????????????????????????????????????????????????????????????????????????????????????????????????????????????????????????????????????????????????????????????????????????????????????????????????????????????????????????????????????????????????????????????????????????????????????????????????????????????????????????????????????????????????????????????????????????????????????????????????????????????????????????????????????????????????????????????????????????????????????????????????????????????????????????????????????????????????????????????????????????????????????????????????????????????????????????????????????????????????????????????????????????????????????

>*Lundqvistomyces*_*tanzaniensis*_TRTC51981

ATTAGCGAGTAA----------AACTCTCT--AA-CACCATTGTGAACG-AACCGAAT------TTAGTTGCTTCGGCGG----------------------------------------------------------------------------------GGTAAAA-CCCGCCGGAAG-----AACGC-AAACACT---CTGTAATG-ATTGTATCTCTGAGTCAACTTTTTTAATAAGTTAAAACTTTC-AACAACGGATCTCTTGGTTCTGGCATCGATGAAGAACGCAGCGAAATGCGATAAGTAATGTGAATTGCAGAATTCAGTGAATCATCGAATCTTTGAACGCACATTGCGCCCGCCAGCACTCTGGCGGGCATGCCTGTTCGAGCGTCATTTCAA-CCATCAAGCCCCA-GGCTT-GCGTTGGGGGCCTGCGG----CTG-CCGCAGCCCCCTAAAAGCAGTGGCGGGCTCGCTGT-CATTCCGAGCGCAGTAG-TTACATCTCGCTCTGGGCGTGGCGGCGGGC-ACCGGCCGTAAAA---CACTC-----TTTCTAAGGTTGACC????????????????????????????????????????????????????AAAGAAACCAACAGGG-ATTGCCCTAGTAACGGCGAGTGAAGCGGCAACAGCTCAAATTTGAAATCTGGCTTCGG-CCCGAGTTGTAATTTGTAGAGGAAGCTTCTGGCGCGGTGCCGTCCGAGTCCCCTGGAACGGGGCGCCATAGAGGGTGAGAGCCCCGTATGGACGGATGCTAAGCCTGTGTGAAGCTCCTTCGACGAGTCGAGTAGTTTGGGAATGCTGCTCAAAATGGGAGGTAAATTCCTTCTAAAGCTAAATATTGGCCAGAGACCGATAGCGCACAAGTAGAGTGATC-------GAAAGATGAAAAGCACTTTGAAAAGAGGGTTAAACAGCACGTGAAATTGTTGAAAGGGAAGCGCTTGTGACCAGACTTGCGCCAGGTTGATCATCCGGTGTTCTCACCGGTGCACTCTGCCTGGCACAGGCCAGCATCAGTTTTGGCGGGGGGATAAAGGGCGCTTGAAAGTAGCTCCTCCGGGAGTGTTATAGCTTGCGTCGTAATACCCTCGCCGGGACTGAGGACCGCGCATCTGCAAGGATGCTGGCGTAATGGTCACCAGCGACCCGTCTTGAAACACGGACCAAGGAGTCAAGGTTTTGCGCGAGTGTTTGGGTGTTAAACCCGCACGCGTAATGAAAGTGAA-CGTAGGTGAGAG----CTTCGGCGCATCATCGACCGATCCTGATGTATTCGGATGGATTTGAGTAAGAGCGTTAAGCCTTGGACCCGAAAGATGGTGAACTATGCTTGGATAGGGTGAAGCCAGAGGAAACTCTGGTGGAGGCTCGCAGCGGTTCTGACGTGCAAATCGATCGTCAAATC-TGAGCATGGGGGCGAAAGACTAATCGAACCATCTAGTAGCTGGTTACCGCCGGTCTCAAGTACTCATTGGCCACAGGCAACTGGGGTGACCAGAAGAAGGCGATGAGCTCGACAGCAGGTGTCTCGCAGGTGTTGAACCGTTACACTTTCGCGTCGACCCTCTCGCATTTGCGTCGCACCAATACTCCCATCGGCCGCGATGGCAAGCTTGCTAAGCCTCGACAACTTCACAACACCCATTGGGGTCTGGTCTGCCCTGCAGAAACGCCAGAGGGTCAAGCTTGCGGCCTGGTCAAGAACCTCTCTTTGATGTGCTACATCAGCGTTGGCACAAATGCTGATCCTATTATTGACTTTATGATCGCCAGAAACATGGAAGTCCTCGAGGAGTACGAGCCGCTTCGATATCCCAACGCAACCAAGGTCTTCGTCAATGGAACCTGGGTTGGTGTTGTCCAGGACCCGAAGAATCTCGTTGGCCTTGTCCAGAACCTTAGAAGGTCAAATATCATTTCCTTCGAGGTCTCCCTGGTTCGCGATATAAGAGATCGAGAGGTCAAGATTTTCTCGGATGCCGGTCGTGTAATGCGACCGTTGTTCGTAGTGGAGCAAGAAGATGATAACCCG------CAGACAAAGGTCTCGAGAGGGCAGCTGGTGTTGAGAAAGGAGCACATTGAGCGATTGGAGCGCGACAGGGAGATTGGAAGATTCGACCCCAATTACTACGGGTGGGATGGCCTTCTTCGAGACGGATGCGTCGAGTACCTCGACGCCGAGGAAGAAGAAACCACCATGATCTGCATGTCGCCTGAAGATCTCGACCATTTCCGAATGACGAAAGT------TTTGGGCATCACGCCTCACGAAGTGGAAGACCCTAGTGCGGGCAACAAGCGCATCCCTACCAAGCTCAACCCGACCACGCATGCGTACACGCATTGCGAGATCCATCCCAGTATGCTGCTCGGCATTTGCGCCAGCATCATTCCGTTCCCGGATCACAACCAGGCTTGTTGACCAAGTCCTCGATGTTGTCCGTCGTGAGGCTGAGGGCTGCGACTGCCTCCAGGGCTTCCAGATCACCCACTCCCTCGGTGGTGGTACCGGTGCCGGTATGGGTACCCTACTTATCTCCAAGATCCGCGAGGAGTTCCCTGACCGCATGATGGCGACTTTCTCCGTCGTCCCCTCCCCCAAGGTCTCGGATACCGTCGTCGAGCCCTACAATGCCACCCTCTCCGTTCACCAGCTTGTTGAGAACTCGGACGAGACCTTCTGCATTGACAACGAGGCTCTCTACGACATCTGCATGAGGACACTCAAGCTCTCCAACCCCTCGTATGGTGACCTTAACCACCTCGTCTCCGCTGTCATGTCCGGTGTCACCGTCTCCCTCCGTTTCCCCGGTCAGCTCAACTCCGATCTCCGCAAGCTCGCCGTGAACATGGTGCCCTTCCCTCGTCTGCACTTCTTCATGGTTGGCTTTGCCCCTCTTACTAGCCGTGGCGCCCACTCTTTCCGTGCCGTCTCGGTTCCCGAGCTCACCCAGCAGATGTTTGACCCCAAGAACATGATGGCTGCTTCTGATTTCCGCAACGGTCGCTACCTCACTTGCTCCGCCATCTT

>*Lundqvistomyces*_*karachiensis*_CBS_657_74

ATTAGCGAGTAA----------TACTCTCT--AA-CACCATTGTGAACG-AACCGAAT------TTAGTTGCTTCGGCGG----------------------------------------------------------------------------------GGTAAAA-CCCGCCGGAAG-----AACGC-AAACACT---CTGTAATG-ATTGTATCTCTGAGT-TAACTTTTTAATAAGTTAAAACTTTC-AACAACGGATCTCTTGGTTCTGGCATCGATGAAGAACGCAGCGAAATGCGATAAGTAATGTGAATTGCAGAATTCAGTGAATCATCGAATCTTTGAACGCACATTGCGCCCGCCAGCACTCTGGCGGGCATGCCTGTTCGAGCGTCATTTCAA-CCATCAAGCCCCA-GGCTT-GCGTTGGGGGCCTGCGG----CTG-CCGCAGCCCCCTAAAAGCAGTGGCGGGCTCGCTGT-CATTCCGAGCGCAGTAG-TTACATCTCGCTCTGGGCGTGGCGGCGGGC-ACCGGCCGTAAAA---CACTC-----TTTCTAAGGTTGACCTCGGATCAGGTAGGAATACCCGCTGAACTTAAGCATATCAATAAGCGGAGGAAAAGAAACCAACAGGG-ATTGCCCTAGTAACGGCGAGTGAAGCGGCAACAGCTCAAATTTGAAATCTGGCTTCGG-CCCGAGTTGTAATTTGTAGAGGAAGCTTCTGGCGCGGTGCCGTCCGAGTCCCCTGGAACGGGGCGCCATAGAGGGTGAGAGCCCCGTATGGACGGATGCTAAGCCTGTGTGAAGCTCCTTCGACGAGTCGAGTAGTTTGGGAATGCTGCTCAAAATGGGAGGTAAATTCCTTCTAAAGCTAAATATTGGCCAGAGACCGATAGCGCACAAGTAGAGTGATC-------GAAAGATGAAAAGCACTTTGAAAAGAGGGTTAAACAGCACGTGAAATTGTTGAAAGGGAAGCGCTTGTGACCAGACTTGCGCCAGGTTGATCATCCGGTGTTCTCACCGGTGCACTCTGCCTGGCACAGGCCAGCATCAGTTTTGGCGGGGGGATAAAGGGCGCTTGAACGTAGCTCCTCCGGGAGTGTTATAGCGTGCGTCGTAATACCCTCGCCGGGACTGAGGACCGCGCATCTGCAAGGATGCTGGCGTAATGGTCACCAGCGACCCGTCTTGAAACACGGACCAAGGAGTCAAGGTTTTGCGCGAGTGTTTGGGTGTTAAACCCGCACGCGTAATGAAAGTGAA-CGTAGGTGAGAG----CTTCGGCGCATCATCGACCGATCCTGATGTATTCGGATGGATTTGAGTAAGAGCGTTAAGCCTTGGACCCGAAAGATGGTGAACTATGCTTGGATAGGGTGAAGCCAGAGGAAACTCTGGTGGAGGCTCGCAGCGGTTCTGACGTGCAAATCGATCGTCAAATC-TGAGCATGGGGGCGAAAGACTAATCGAACCATCTAGTAGCTGGTTACCGCCGGTCTCAAGTACTCGTTGGCCACAGGCAACTGGGGTGACCAGAAGAAGGCGATGAGCTCAACAGCAGGTGTCTCGCAGGTGTTGAACCGTTACACTTTCGCGTCGACCCTCTCGCATTTGCGTCGCACCAATACTCCCATCGGCCGCGATGGCAAGCTCGCTAAGCCTCGACAACTTCACAACACCCATTGGGGTCTGGTCTGCCCTGCAGAAACGCCAGAGGGTCAAGCTTGCGGCCTGGTCAAGAACCTCTCTTTGATGTGCTACATCAGCGTTGGCACGAATGCTGATCCTATTATCGACTTTATGATAGCCAGAAACATGGAAGTCCTCGAGGAGTACGAGCCGCTTCGATATCCCAACGCAACCAAGGTCTTCGTCAACGGAACCTGGGTTGGTGTTGTCCAGGACCCGAAGAATCTCGTTGGCCTTGTCCAGAACCTTAGAAGGTCAAATATCATTTCCTTCGAGGTCTCACTGGTTCGCGATATAAGAGATCGAGAGTTCAAGATTTTCTCGGATGCCGGTCGTGTAATGCGACCGTTGTTCGTAGTGGAGCAAGAAGATGATAACCCG------CAGACAAAGGTCTCGAGAGGGCAGCTGGTGTTGAGAAAGGAGCACATTGAGCGATTGGAGCGCGACAGAGAGATTGGAAGATTCGACCCCAATTACTACGGGTGGGATGGCCTTCTTCGAGACGGATGCGTCGAGTACCTCGACGCCGAGGAAGAAGAAACCACCATGATCTGCATGTCGCCTGAAGATCTCGACCATTTCCGAATGACGAAAGT------TTTGGGCATCACGCCTCACGAAGTGGAAGACCCTAGTGCGGGCAACAAGCGCATCCCTACCAAGCTCAACCCGACCACTCATGCGTACACGCATTGCGAGAT?????????????????????????????????????????????????????????????????????????????????????????????????????????????????????????????TTCCAGATCACCCACTCCCTCGGTGGTGGTACCGGTGCCGGTATGGGTACCCTACTTATCTCCAAGATCCGCGAGGAGTTCCCTGACCGCATGATGGCGACTTTCTCCGTCGTCCCCTCCCCCAAGGTCTCGGATACCGTCGTCGAGCCCTACAACGCCACCCTCTCCGTGCACCAGCTTGTTGAGAACTCGGACGAGACCTTCTGCATTGACAACGAGGCTCTCTACGACATCTGCATGAGGACACTCAAGCTCTCCAACCCCTCGTATGGTGACCTTAACCACCTCGTCTCCGCTGTCATGTCCGGTGTCACCGTCTCCCTCCGTTTCCCCGGTCAGCTCAACTCTGATCTCCGCAAGCTCGCCGTGAACATGGTGCCCTTCCCTCGTCTGCACTTCTTTATGGTTGGCTTTGCCCCTCTTACTAGCCGTGGCGCCCACTCTTTCCGTGCCGTCTCGGTTCCCGAGCTCACCCAGCAGATGTTCGACCCCAAGAACATGATGGCTGCTTCTGATTTCCGCAACGGTCGCTACCTCACTTGCTCCGCCATCTT

>*Schizothecium*_*selenosporum*_CBS_109403

ATTACAGAGTTGC---------AAAACTCC--CA--ACCATTGTGAACC-TACCTC--------ACCGTTGCTTCGGCGG------------------GTGGCCCCACCC--------------GGGCCGCGCCGG-------CCCCCCC--------GGGGCCGGCAA-CCCGTCAGAGG-----ACCCA-CACTCTT---AGTCATCATTTGGCCTCTCTGAGT-AACTTATACAATAAGTCAAAACTTTC-AACAACGGATCTCTTGGTTCTGGCATCGATGAAGAACGCAGCGAAATGCGATACGTAATGTGAATTGCAGATTTCAGTGAATCATCGAATCTTTGAACGCACATTGCGCCCGCCAGTATTCTGGCGGGCATGCCTGTTCGAGCGTCATTTCAA-CCATCAAGCCCCC-GGCTT-GTGTTGGGGCCCTGCGG----CTG-CCGCAGGCCCCTAAATCCAGTGGCGGGCTCGTCGT-CGTACCGAGCGCAGTAA--ACATCCTCGCTCGGGGACC-CCGACGGGT-GCCGGCCGTGAAA---CCCCC---CCTTCTCAAGGTTGACCTCGGATCAGGTAGGAATACCCGCTGAACTTAAGCATATCAATAAGCGGAGGAAAAGAAACCAACAGGG-ATTGCCCCAGTAACGGCGAGTGAAGCGGCAACAGCTCAAATTTGAAATCTGGCCTCGG-CCCGAGTTGTAATTTGCAGAGGAAGCTTCTGGTGCGGCGCCGTCCGAGTCTCCTGGAACGGAGCGCCATAGAGGGTGAGAGCCCCGTATGGACGGACGCCAAACCTGTGTGAAGCTCCTTCGACGAGTCGAGTAGTTTGGGAATGCTGCTCAAAATGGGAGGTAAATTCCTTCTAAAGCTAAATACCGGCCAGAGACCGATAGCGCACAAGTAGAGTGATC-------GAAAGATGAAAAGCACTTTGAAAAGAGGGTTAAACAGCACGTGAAATTGTTGAAAGGGAAGCGCTTGTG--CAGACTCGGGCGCGGCGGATCATCCGGTGTTCTCACCGGTGCACTCCGCCGCGCCCGGGCCAGCATCGGCTTCCGCCGGGGGATAAAGGTCCCGGGAACGTAGCTCCTCCGGGAGTGTTATAGCCCGGGGCGCAATGCCCCGGCGGGGGCCGAGGACCGCGCAT-TGCAAGGATG??????????????????????????????????????????????????????????????????????????????????????????????????????????????????????????????????????????????????????????????????????????????????????????????????????????????????????????????????????????????????????????????????????????????????????????????????????????????????????????????????????????????????????????????????????????????????????????????????????????????????????????????????????????????????????TCGACCCTCTCTCATTTGCGCCGCACCAACACGCCCATCGGCCGCGACGGAAAGCTCGCCAAGCCGCGCCAGCTTCACAACACGCATTGGGGCCTCGTCTGTCCAGCCGAGACGCCCGAGGGCCAGGCCTGCGGGCTGGTCAAGAATCTGTCGCTCATGTGCTACATCAGCGTGGGCACCAACGCGGAGCCCATTATCGAGTTCATGATCGCACGCAACATGGAGGTCTTGGAAGAGTACGAGCCACTGCGCTCCCCCAATGCCACCAAGATCTTTGTCAACGGCACGTGGGTCGGCGTGCACCACGACGCCAAGCACCTCGTGCACCTTGTCCAGGGCCTCCGGCGATCCAACATTGTGAGCTTCGAGGTGTCGCTGGTCCGGGATATCCGAGACCGCGAGTTCAAGATCATGTCGGATGCCGGCCGCGTCATGAGGCCCCTCTTCGTCGTCGAGACCGAGGACGAGAGCTCC---------ACGGGGGTGGAAAAGGGCGAGCTGGTGCTCACCAAGACCCACGTCCAGAAGCTGGCCAACGACAAGCTGATTGGGAAATACCACAAGGACTACTTTGGGTGGCAAGGCCTCTTGCAATCAGGCGCCGTCGAATACCTCGACGCCGAGGAGGAGGAGACGGCCATGATCTCCATGTCGCCCGAGGACCTCGACCATTTCCGCGACGCCAAGGC------GAGAAACTTTGAGGAACCCGAGGGCAAGGTGGTGACCGAGGGCAACAAGCGCATCCCGACGAGGATCAACCCGACGACCTACATGTATACGCACTGCGAGATCCACCCCAGCATGCTGCTCGGCATCTGTGCGAGCATCATCCCTTTCCCGGACCACAACCAG??????????????????????????????????????????????????????????????????????????????????????????????????????????????????????????????????????????????????????????????????????????????????????????????????????????????????????????????????????????????????????????????????????????????????????????????????????????????????????????????????????????????????????????????????????????????????????????????????????????????????????????????????????????????????????????????????????????????????????????????????????????????????????????????????????????????????????????????????????????????????????????????????????????????????????????????????????????

>*Podospora*_*serotina*_CBS_252_71

ATTACCGAGTTCT---------TACAGAACC-CA-ACCCTGTGTGATAA-GTTCTCACCC---GAACTTTTCCACTACAG------------------GTCTCGGTCCG----------------------------------CCGCGGT------GGACTTCGAGGCG-CCTGTAGGA-------AACCC-AAACCTC-TGTATCATTAACACAAATATCTGAGCGACTTTACATAAAGAGTCAAAACTTTC-AACAACGGATCTCTTGGTTCTGGCATCGATGAAGAACGCAGCGAAATGCGATACGTAATGTGAATTGCAGAATTCAGTGAATCATCGAATCTTTGAACGCACATTGCGCCCGCCAGTATTCTGGCGGGCATGCCTGTCCGAGCGTCATTTCAACCCATCAAGCCCAG-CGCTT-GTGTTGGAGCCCTGCGG----CCG-CCGCAGCCTCCCAAAATTAGTGGCGGGCTCGCTAT-CACGCTGAGTGCAGTAGTATTCTTCTCACTCCTGCGGT-GTAGCGGGTTACCAGCCGTAAAA---CCCTT----TTACCTAAAGTTGACCTCGGATCAGGTAGGAATACCCGCTGAACTTAAGCATATCA????????????????????????????????????????AACGGCGAGTGAAGCGGCAACAGCTCAAATTTGAAATCTGGCAACAG-CCCGAGTTGTAATTTGCAGAGGAAGCTTCTGGCGACGCACTGTCTAAGTCCCCTGGAACGGGGCGCCACAGCGGGTGAGAGCCCCATGT-GATGGCTGCGGACCCAGTGTGAAGCTCCTTCGACGAGTCGAGTAGTTTGGGAATGCTGCTCAAAATGGGAGGTAAATTCCTTCTAAAGCTAAATATTGGCCAGAGACCGATAGCGCACAAGTAGAGTGATC-------GAAAGATGAAAAGCACTTTGAAAAGAGGGTTAAACAGCACGTGAAATTGTTGAAAGGGAAGCGCTCATGACCAGACTTGCGCTGGGCTGATCATCCGGTGTTCTCACCGGTGCACTCTGCCCGGCTCAGGCCAGCATCGGTTTTGGCGGGGGGATAAAGGCGCCGGGAACGTAGCTCCTCCGGGAGTGTTATAGCCCGGTGTGCAATACCCCCGCTGGGACCGAGGTCCGCGCATC-GCAAGGATGCTGGCGTAATGGTCATCAGCGACCCGTCTTGAAACACGGACCAAGGAGTCAAGGTTTTGCGCGAGTGTTTGGGTGTCAAACCCGCRCGCGTAATGAAAGTGAA-CGTAGGTGAGAG----CTTCGGCGCATCATCGACCGATCCTGAAGTTTACGGACGGATTTGAGTAGGAGCGTTAAGCCTTGGACCCGAAAGATGGTGAACTATGCTTGGATAGGGTGAAGCCAGAGGAAACTCTGGTGGAGGCTCGCAGCGGTTCTGACGTGCAAATCGATCGTCAAATC-TGAGCATGGG?????????????????????????????????????????????????????????????????????????????????????????????????????????????????????????????????????????????????????????????????????????????????????????????????????????????????????????????????????????????????????????????????????????????????????????????????????????????????????????????????????????????????????????????????????????????????????????????????????????????????????????????????????????????????????????????????????????????????????????????????????????????????????????????????????????????????????????????????????????????????????????????????????????????????????????????????????????????????????????????????????????????????????????????????????????????????????????????????????????????????????????????????????????????????????????????????????????????????????????????????????????????????????????????????????????????????????????????????????????????????????????????????????????????????????????????????????????????????????????????????????????????????????????????????????????????????????????????????????????????????????????????????????????????????????????????????????????????????????????????????????????????????????????????????????????????????????????????????????????????????????????????????????????????????????????????????????????????????????????????????????????????????????????????????????????????????????????????????????????????????????????????????????????????????????????????????????????????????????????????????????????????????????????????????????????????????????????????????????????????????????????????????????????????????????????????????????????????????????????????????????????????????????????

>*Rinaldiella*_*pentagonospora*_CBS_132344

ATTACAGGGTAGCTTTATGCTGTAAACCCC--CA--ACCATTGTGAACG-AAGCTTATTC----GCAGTTGCCTCAGCGG------------------GTGGTTAT-----------------------------------------------------------GCTA-CCCGCCTGAAG-----CACGC-AAACTCT----TGTTTTA-ATGTCATCTCTGAGT-AACT--TTTAATAAGTCAAAACTTTC-AACAACGGATCTCTTGGTTCTGGCATCGATGAAGAACGCAGCGAAATGCGATAAGTAATGTGAATTGCAGAATTCAGTGAATCATCGAATCTTTGAACGCACATTGCGCCCGCTAGTATTCTGGCGGGCATGCCTGTTCGAGCGTCATTTCAA-CCATCAAGCCCTA-GGCTT-GTGTTGGGGCTCTGCGG----CTG-CCGCAGTCCCCTAAAAGCAGTGGCGGACTCGCTGT-CATACCGAGCGCAGTAG-CATAT-CTCGCTCTGGGCGTGGCGGCGGGT-ACCGGCCGTTAAA---CAATC------CATCAAGGTTGACCTCGGATCAGGTAGGAATACCCGCTGAACTTAAGCATATCAA???????????AAAGAAACCAACAGGG-ATTGCCCTAGTAACGGCGAGTGAAGCGGCAACAGCTCAAATTTGAAATCTGGCTTCGG-CCCGAGTTGTAATTTGCAGAGGAAGCTTCTGGCGCGGCGCTGTCCGAGTCCCCTGGAACGGGGCGCCATAGAGGGTGAGAGCCCCGTATGGATGGATGCCTAGCCTGTGTGAAGCTCCTTCGACGAGTCGAGTAGTTTGGGAATGCTGCTCAAAATGGGAGGTAAATTCCTTCTAAAGCTAAATATTGGCCAGAGACCGATAGCGCACAAGTAGAGTGATC-------GAAAGATGAAAAGCACTTTGAAAAGAGGGTTAAATAGCACGTGAAATTGTTGAAAGGGAAGCGCTTGTGACCAGACTTGCGCCAGGCTGATCATCCGGTGTTCTCACCGGTGCACTCTGCCTGGCTCAGGCCAGCATCGGTTTTGGTGGGGGGATAAAGACCTAGGGAACGTAGCTCTTTCGGGAGTGTTATAGCCCTGGGTGTAATGCCCCCGCTGGGACCGAGGTTCGCGCATCTGCAAGGATGCTGGCGTAATGGTCATCAGCGACCCGTCTTGAAACACGGACCAAGGAGTCAAGGTTTTGCGCGAGTGTTTGGGTGTTAAACCCGCACGCGTAATGAAAGTGAA-CGTAGGTGAGAG----CTTCGGCGCATCATCGACCGATCCTGATGTATTCGGATGGATTTGAGTAGGAGCGTTAAGCCTTGGACCCGAAAGATGGTGAACTATGCTTGGATAGGGTGAAGCCAGAGGAAACTCTGGTGGAGGCTCGCAGCGGTTCTGACGTGCAA???????????????????????????????????????????????????????????????????GGGCTGAAGTACTCGCTCGCAACTGGTAACTGGGGTGATCAGAAGAAGGCGATGAGCTCTACTGCCGGTGTTTCGCAAGTGTTGAACCGATATACATTTGCTTCCACACTTTCCCACTTGCGGCGTACCAACACACCCATCGGTCGCGACGGGAAACTCGCCAAACCCCGACAACTGCACAACACTCACTGGGGCTTGGTCTGCCCCGCAGAGACCCCCGAAGGCCAAGCATGCGGTCTTGTGAAGAACCTTTCTCTCATGTGCTACGTCAGCGTCGGCACTAATGCCGAGCCGATTGTCGATTTCATGATAGCTCGGAATATGGAAGTCCTCGAGGAGTATGAACCCTTGCGGTACCCGAATGCTACCAAAGTCTTCGTCAACGGCACCTGGGTTGGTGTTCACCAAGACCCGAAGCACTTGGTCGGTTTGGTGCAAGGCCTGAGAAGAACCAACGTCATCTCTTTTGAGGTCTCGCTAGTGAGAGACATTCGAGACAGGGAGTTCAAGATCTTCTCGGACGCCGGCCGAGTTATGCGACCGCTCTTTGTTGTGGAGCAAGAGGACGACAATCCC---------GCCAAGATTGAGAAGGGACAGTTGGTCTTGACGAAATCACACGTACAGCGTCTGGAGAACGATAAGGTAATTGGTCGATACCACAAGGATTACTTCGGTTGGGATGGCCTGGTGAGGGAAGGCTGCGTCGAGTATCTAGATGCCGAGGAGGAGGAGACAACCATGATTTGCATGACCCCTGAGGACCTGGACACCTACCGCTTGACGAAGCT?????????????????????????????????????????????????????????????????????????????????????????????????????????????????????????????????????????????????????????????????????????GCTTGTCGACCAAGTTCTCGATGTCGTCCGTCGCGAGGCCGAGGGCTGCGACTGCCTCCAGGGCTTCCAGATTACCCACTCACTCGGCGGTGGTACCGGTGCTGGTATGGGCACCCTCCTTATCTCCAAGATTCGCGAGGAGTTCCCCGACCGCATGATGGCGACTTTCTCGGTCGTTCCCTCACCCAAGGTGTCGGATACCGTTGTTGAGCCCTACAACGCCACTCTCTCCGTCCACCAACTCGTTGAGAACTCGGATGAGACCTTCTGCATTGACAACGAGGCTCTTTACGACATCTGCATGCGGACGCTCAAGCTGTCCAACCCCTCGTACGGCGACCTCAACCACCTGGTCTCTGCCGTCATGTCGGGCGTCACCGTTTCTCTCCGTTTCCCCGGCCAGCTCAACTCTGACCTCCGCAAGCTTGCCGTCAACATGGTTCCCTTCCCGCGTCTCCACTTCTTCATGGTGGGCTTCGCTCCTCTTACCAGCCGTGGCGCGCACTCTTTCCGTGCCGTCTCGGTGCCCGAGCTTACCCAGCAGATGTTCGACCCCAAGAACATGATGGCCGCTTCTGACTTCCGCAACGGTCGCTACCTTACCTGCTCTGCCATTTT

>*Schizothecium*_*aloides*_CBS_879_72

ATTACAGAGTTGC---------GAAACTCC--CA--ACCATTGTGAACC-TACCTC--------ACCGTTGCTTCGGCGG------------------GTGGCCCCCTC-------------CGGGGCCGCGCCGG-------CCCCCCC--------GGGGTCGGCAA-CCCGTCAGAGG-----ACCGA-AACTCTT---TGTAACCA-CTGGCCTCTCTGAGT-AACTTATACAATAAGTCAAAACTTTC-AACAACGGATCTCTTGGTTCTGGCATCGATGAAGAACGCAGCGAAATGCGATACGTAATGTGAATTGCAGATTTCAGTGAATCATCGAATCTTTGAACGCACATTGCGCCCGCTAGTATTCTGGCGGGCATGCCTGTTCGAGCGTCATTTCAA-CCATCAAGCCCCCGGGCTT-GCGTTGGAGCCCTGCGG----CTG-CCGCAGGCTCCCAAATCCAGTGGCGGGCTCGTCGT-CGTACCGAGTGCAGTAA--ACATCCTCGCTCAGGGAAC-GCGTC-GGT-TCTTGCCGTGAAA---CCCCC--CCTATATCAAGGTTGACCTCGGATCAGGTAGGAATACCCGCTGAACTTAAGCATATCAATAAGCGGAGGAA???????CAACAGGG-ATTGCCCCAGTAACGGCGAGTGAAGCGGCAACAGCTCAAATTTGAAATCTGGCCTCGG-CCCGAGTTGTAATTTGTAGAGGAAGCTTCTGGTGCGGCGCCGTCCGAGTCTCCTGGAACGGAGCGCCATAGAGGGTGAGAGCCCCGTATGGTCGGATGCCAAACCTGTGTGAAGCTCCTTCGACGAGTCGAGTAGTTTGGGAATGCTGCTCTAAATGGGAGGTAAATTCCTTCTAAAGCTAAATATTGGCCAGAGACCGATAGCGCACAAGTAGAGTGATC-------GAAAGATGAAAAGCACTTTGAAAAGAGGGTTAAACAGCACGTGAAATTGTTGAAAGGGAAGCGCTTGTGACCAGACTTGAGGGCGGCGGATCATCCGGTGTTCTCACCGGTGCACTCCGCCGCTCCCAGGCCAGCATCGGTTTCCGCGGGGGGACAAAGGTCCCGGGAACGTAGCTCCTCCGGGAGTGTTATAGCCCGGGGCGCAATGCCCCCGCGGGGACCGAGGACCGCGCATCTGCAAGGATGCTGGCGTAATGGTCATCAGCGACCCGTCTTGAAACACGGACCAAGGAGTCAAGGTTTTGCGCGAGTGTTTGGGTGTCAAACCCGCACGCGTAATGAAAGTGAA-CGTAGGTGAGAG----CTTCGGCGCATCATCGACCGATCCTGATGTATTCGGATGGATTTGAGTAGGAGCGTTAAGCCTTGGACCCGAAAGATGGTGAACTATGCTTGGATAGGGTGAAGCCAGAGGAAACTCTGGTGGAGGCTCGCAGCGGTTCTGACGTGCAAATCGATCGTCAAATC-TGAGCATGGGGGCGAAAGACTAATCGA????????????????????????????????????????????????????????????????????????????????????????????????????????????????????????????????????????????????????????????????????????????????????????????????????????????????????????????????????????????????????????????????????????????????????????????????????????????????????????????????????????????????????????????????????????????????????????????????????????????????????????????????????????????????????????????????????????????????????????????????????????????????????????????????????????????????????????????????????????????????????????????????????????????????????????????????????????????????????????????????????????????????????????????????????????????????????????????????????????????????????????????????????????????????????????????????????????????????????????????????????????????????????????????????????????????????????????????????????????????????????????????????????????????????????????????????????????????????????????????????????????????????????????????????????????????????????????????????????????????????????????????????????????????????????????????????????????????????????????????????????????????????????????????????????????????????????????????????????????????????????????????????????????????????????????????????????????????????????????????????????????????????????????????????????????????????????????????????????????????????????????????????????????????????????????????????????????????????????????????????????????????????????????????????????????????????????????????????????????????????????????????????????????????????????????????????????????????????????????????????????????

>*Schizothecium*_*carpinicola*_CBS_228_87

ATTACAGAGTTGC---------AAAACTCC--CA--ACCATTGTGAACC-TACCTC--------ACCGTTGCTTCGGCGG------------------GTGGCCCCCTCC-----------GCGGGGCCGCGCCGG-------CCCCCCC-------GGGGGTCGGCAA-CCCGTCAGAGG-----ACCGA-AACTCTT---AGTAACCA-CTGGCCTCTCTGAGT-AACTTATACAATAAGTCAAAACTTTC-AACAACGGATCTCTTGGTTCTGGCATCGATGAAGAACGCAGCGAAATGCGATACGTAATGTGAATTGCAGATTTCAGTGAATCATCGAATCTTTGAACGCACATTGCGCCCGCTAGTATTCTGGCGGGCATGCCTGTTCGAGCGTCATTTCAA-CCATCAAGCCCCC-GGCTT-GCGTTGGAGCCCTGCGG----CTG-CCGCAGGCTCCCAAATCCAGTGGCGGGCTCGTCGT-CGTACCGAGTGCAGTAA--ACATCCTCGCTCAGGGAAC-GCGTCGGGT-TCTTGCCGTGAAA---CCCCC--CCCACATCAAGGTTGACCTCGGATCAGGTAGGAATACCCGCTGAACTTAAGCATATCAATAAGCGGAGGAA????????????????ATTGCCCCAGTAACGGCGAGTGAAGCGGCAACAGCTCAAATTTGAAATCTGGCCTCGG-CCCGAGTTGTAATTTGTAGAGGAAGCTTCTGGTGCGGCGCCGTCCGAGTCTCCTGGAACGGAGCGCCATAGAGGGTGAGAGCCCCGTATGGTCGGATGCCAAACCTGTGTGAAGCTCCTTCGACGAGTCGAGTAGTTTGGGAATGCTGCTCTAAATGGGAGGTAAATTCCTTCTAAAGCTAAATATTGGCCAGAGACCGATAGCGCACAAGTAGAGTGATC-------GAAAGATGAAAAGCACTTTGAAAAGAGGGTTAAACAGCACGTGAAATTGTTGAAAGGGAAGCGCTTGTGACCAGACTTGAGCGCGGCGGATCATCCGGTGTTCTCACCGGTGCACTCCGCCGTGCCCAGGCCAGCATCGGTTTCCGCGGGGGGACAAAGGTCCCGGGAACGTAGCTCCTCCGGGAGTGTTATAGCCCGGGGCGCAATGCCCCCGCGGGGACCGAGGACCGCGCATCTGCAAGGATGCTGGCGTAATGGTCATCAGCGACCCGTCTTGAAACACGGACCAAGGAGTCAAGGTTTTGCGCGAGTGTTTGGGTGTTAAACCCGCACGCGTAATGAAAGTGAA-CGTAGGTGAGAG----CTTCGGCGCATCATCGACCGATCCTGATGTATTCGGACGGATTTGAGTAGGAGCGTTAAGCCTTGGACCCGAAAGATGGTGAACTATGCTTGGATAGGGTGAAGCCAGAGGAAACTCTGGTGGAGGCTCGCAGCGGTTCTGACGTGCAAATCGATCGTCAAATC-TGAGCATGGGGGCGAAAGACTAATCGAT???????????????????????????????????????????????????????????????????????????????????????????????????????????????????????????????????????????????????????????????????????????????????????????????????????????????????????????????????????????????????????????????????????????????????????????????????????????????????????????????????????????????????????????????????????????????????????????????????????????????????????????????????????????????????????????????????????????????????????????????????????????????????????????????????????????????????????????????????????????????????????????????????????????????????????????????????????????????????????????????????????????????????????????????????????????????????????????????????????????????????????????????????????????????????????????????????????????????????????????????????????????????????????????????????????????????????????????????????????????????????????????????????????????????????????????????????????????????????????????????????????????????????????????????????????????????????????????????????????????????????????????????????????????????????????????????????????????????????????????????????????????????????????????????????????????????????????????????????????????????????????????????????????????????????????????????????????????????????????????????????????????????????????????????????????????????????????????????????????????????????????????????????????????????????????????????????????????????????????????????????????????????????????????????????????????????????????????????????????????????????????????????????????????????????????????????????????????????????????????????????????

>*Schizothecium*_*glutinans*_CBS_134_83

ATTACAGAGTTGC---------AAAACTCC--CA--ACCATTGTGAACC-TACCTC--------ACCGTTGCTTCGGCGG------------------GTGGCCCGCTAAT----------CCCAGGCCGCGCCGG-------CCCCTTTC---TAGGGGGCCCGGCAA-CCCGTCAGAGG-----CCCGA-AACTCTT---CGTCATCA-GTGGCCTCTCTGAGT-AACTTATACAATAAGTCAAAACTTTC-AACAACGGATCTCTTGGTTCTGGCATCGATGAAGAACGCAGCGAAATGCGATACGTAATGTGAATTGCAGATTTCAGTGAATCATCGAATCTTTGAACGCACATTGCGCCCGCCAGTATTCTGGCGGGCATGCCTGTTCGAGCGTCATTTCAA-CCATCAAGCGCCTGCGCTT-GTGTTGTAGCCCTGCGG----CTG-CCGCAGGCTCCCAAACCCAGTGGCGGGCTCGTCGT-CGTACCGAGTGCAGTAAATTTA-CCACGCTCAGGGCGC-GCGACGGGTGACCGGCCGTAAAA---CCCCCCAACTTTATCAAGGTTGACCTCGGATCAGGTAGGAATACCCGCTGAACTTAAGCATATCAATAAGCGGAGGA????????CAACAGGG-ATTGCCCCAGTAACGGCGAGTGAAGCGGCAACAGCTCAAATTTGAAATCTGGCCTCGG-CCCGAGTTGTAATTTGCAGAGGAAGCTTCTGGTGCGGCGCTGTCCGAGTCTCCTGGAACGGAGCGCCATAGAGGGTGAGAGCCCCGTATGGACGGATGCCAAACCTGTGTGAAGCTCCTTCGACGAGTCGAGTAGTTTGGGAATGCTGCTCTAAATGGGAGGTAAATTCCTTCTAAAGCTAAATATTGGCCAGAGACCGATAGCGCACAAGTAGAGTGATC-------GAAAGATGAAAAGCACTTTGAAAAGAGGGTTAAACAGCACGTGAAATTGTTGAAAGGGAAGCGCTTGTGACCAGACTTGAGCGCGGCGGATCATCCAGTGTTCTCACCGGTGCACTCCGCCGCGTCCAGGCCAGCATCGGTTTCCACGGGGGGACAAAGGTCCCGGGAACGTAGCTCTTTCGAGAGTGTTATAGCCCGGGGCGCAATGCCCCCGCGGGGACCGAGGACCGCGCATCTGCAAGGATGCTGGCGTAATGGTCATCAGCGACCCGTCTTGAAACACGGACCAAGGAGTCAAGGTTTTGCGCGAGTGTTTGGGTGTTAAACCCGCACGCGTAATGAAAGTGAA-CGTAGGTGAGAG----CTTCGGCGCATCATCGACCGATCCTGATGTATTCGGATGGATTTGAGTAGGAGCGTTAAGCCTTGGACCCGAAAGATGGTGAACTATGCTTGGATAGGGTGAAGCCAGAGGAAACTCTGGTGGAGGCTCGCAGCGGTTCTGACGTGCAAATCGATCGTCAAATC-TGAGCATGGGGGCGAAAGACTAATCGA????????????????????????????????????????????????????????????????????????????????????????????????????????????????????????????????????????????????????????????????????????????????????????????????????????????????????????????????????????????????????????????????????????????????????????????????????????????????????????????????????????????????????????????????????????????????????????????????????????????????????????????????????????????????????????????????????????????????????????????????????????????????????????????????????????????????????????????????????????????????????????????????????????????????????????????????????????????????????????????????????????????????????????????????????????????????????????????????????????????????????????????????????????????????????????????????????????????????????????????????????????????????????????????????????????????????????????????????????????????????????????????????????????????????????????????????????????????????????????????????????????????????????????????????????????????????????????????????????????????????????????????????????????????????????????????????????????????????????????????????????????????????????????????????????????????????????????????????????????????????????????????????????????????????????????????????????????????????????????????????????????????????????????????????????????????????????????????????????????????????????????????????????????????????????????????????????????????????????????????????????????????????????????????????????????????????????????????????????????????????????????????????????????????????????????????????????????????????????????????????????????

>*Schizothecium*_*inaequale*_CBS_356_49

ATTACAGAGTTGC---------AAAACTCC--CA--ACCATTGTGAACC-TACCTC--------ACCGTTGCCTCGGCGG------------------GTGGCCCCCAC-------------CCGGGCCGCGCCGG-------CCCCACC---------GGGCCGGCAA-CCCGTCAGAGG-----ACCGC-AACTCTT---AGTCATCA-TTGGCCTCTCTGAGT-AACTTATACAATAAGTCAAAACTTTC-AACAACGGATCTCTTGGTTCTGGCATCGATGAAGAACGCAGCGAAATGCGATACGTAATGTGAATTGCAGATTTCAGTGAATCATCGAATCTTTGAACGCACATTGCGCCCGCCAGTATTCTGGCGGGCATGCCTGTTCGAGCGTCATTTCAA-CCATCAAGCGCCC-CGCTT-GTGTTGGGGCCCTGCGG----CTG-CCGCAGACCCCTAAATCCAGTGGCGGGCTCGTCGT-CGTACCGAGTGCAGTAA--ACATCCTCGCTCAGGGCAC-GCGCCGGGTCTCTTGCCGTGAAA---CACCC--CACATATCAAGGTTGACCTCGGATCAGGTAGGAATACCCGCTGAACTTAAGCATATCAATAAGCGGAGGAAAAGAAACCAACAGGG-ATTGCCCCAGTAACGGCGAGTGAAGCGGCAACAGCTCAAATTTGAAATCTGGCCTCGG-CCCGAGTTGTAATTTGCAGAGGAAGCTTCTGGTGCGGCGCCGTCCGAGTCTCCTGGAACGGAGCGCCATAGAGGGTGAGAGCCCCGTATGGACGGATGCCAAACCTGTGTGAAGCTCCTTCGACGAGTCGAGTAGTTTGGGAATGCTGCTCTAAATGGGAGGTAAATTCCTTCTAAAGCTAAATATTGGCCAGAGACCGATAGCGCACAAGTAGAGTGATC-------GAAAGATGAAAAGCACTTTGAAAAGAGGGTTAAACAGCACGTGAAATTGTTGAAAGGGAAGCGCTTGTGACCAGACTTGAGCGCGGCGGATCATCCGGTGTTCTCACCGGTGCACTCCGCCGCGCCCAGGCCAGCATCGGCTTCCGCGGGGGGACAAAGGTCCCGGGAACGTAGCTCCTCCGGGAGTGTTATAGCCCGGGGCGCAATGCCCCCGCGGGGGCCGAGGACCGCGCATCTGCAAGGATG????????????????????????????????????????????????????????????????????????????????????????????????????????????????????????????????????????????????????????????????????????????????????????????????????????????????????????????????????????????????????????????????????????????????????????????????????????????????????????????????????????????????????????????????????????????????????????????????????????????????????????????????????????????????TTTGCTTCGACCCTCTCTCATTTGCGCCGCACCAACACCCCCATCGGCCGCGACGGAAAGCTGGCCAAGCCGCGCCAGCTGCACAACACCCATTGGGGCCTCGTCTGTCCGGCCGAGACGCCCGAGGGCCAGGCCTGCGGGCTGGTCAAGAATTTGTCCCTCATGTGCTACATCAGCGTGGGCACGAACGCGGAGCCCATTGTCGAGTTCATGATTGCGCGCAACATGGAGGTCTTGGAAGAGTACGAGCCACTGCGCTCCCCCAACGCCACCAAGATCTTTGTAAACGGTACGTGGGTCGGCGTGCACCACGACGCCAAGCATCTCGTGCACCTTGTCCAGGGCCTCCGGCGATCCAACATTGTGAGCTTCGAAGTGTCGCTGGTCCGGGATATCCGAGACCGCGAGTTCAAGATCATGTCGGATGCCGGCCGCGTCATGAGGCCCCTCTTCGTTGTCGAGACCGAGGACGAGAGCTCC---------ACGGGAGTAGAAAAGGGCGAGCTGGTGCTCACCAAGACCCACGTCCAGAAGCTGGCCAACGACAAGCTGATTGGGAAATACCACAAGGACTACTTCGGGTGGCAAGGCCTCTTGCAATCGGGCGCCGTCGAATACCTCGACGCCGAGGAGGAGGAGACGTCCATGATCTCCATGTCGCCCGAGGACCTCGACCATTTCCGCGACGCCAAGGC------GAGAAACTTTGAGGAACCCGAGGGCAAGGTGGTAACCGAGGGCAACAAGCGCATCCCGACGAGGATCAACCCGACGACCTACATGTATACGCACTGCGAGATCCACCCCAGCATGCTGCTCGGCATCTGCGCGAGCATCATCCCTTTCCCCGACCACAACCAG??????????????????????????????????????????????????????????????????????????????????????????????????????????????????????????????????????????????????????????????????????????????????????????????????????????????????????????????????????????????????????????????????????????????????????????????????????????????????????????????????????????????????????????????????????????????????????????????????????????????????????????????????????????????????????????????????????????????????????????????????????????????????????????????????????????????????????????????????????????????????????????????????????????????????????????????????????????

>*Schizothecium*_*minicauda*_CBS_227_87

ATTACAGAGTTGC---------AAAACTCC--CA--ACCATTGTGAACC-TACCTC--------ACCGTTGCTTCGGCGG------------------GTGGCCCCCAC-------------CCGGGCCGCGCCGG-------CTCCACC--------GGAGCCGGCAA-CCCGTCAGAGG-----ACCGA-AACTCTT---AGTAATTA-CTGGCCTCTCTGAGT-AACTTATACAATAAGTCAAAACTTTC-AACAACGGATCTCTTGGTTCTGGCATCGATGAAGAACGCAGCGAAATGCGATACGTAATGTGAATTGCAGATTTCAGTGAATCATCGAATCTTTGAACGCACATTGCGCCCGCCAGTATTCTGGCGGGCATGCCTGTTCGAGCGTCATTTCAA-CCATCAAGCGCCC-CGCTT-GTGTTGGGGCCCTGCGG----CTG-CCGCAGACCCCTAAATCCAGTGGCGGGCTCGTCGT-CGTACCGAGTGCAGTAA--ACATCCTCGCTCAGGGCAC-GCGGCGGGTTCCTTGCCGTGAAA---CACCC--CACATATCAAGGTTGACCTCGGATCAGGTAGGAATACCCGCTGAACTTAAGCATATCAATA?????????AAAGAAACCAACAGGG-ATTGCCCCAGTAACGGCGAGTGAAGCGGCAACAGCTCAAATTTGAAATCTGGCCTCGG-CCCGAGTTGTAATTTGCAGAGGAAGCTTCTGGTGCGGCGCCGTCCGAGTCTCCTGGAACGGAGCGCCATAGAGGGTGAGAGCCCCGTATGGACGGATGCCAAACCTGTGTGAAGCTCCTTCGACGAGTCGAGTAGTTTGGGAATGCTGCTCTAAATGGGAGGTAAATTCCTTCTAAAGCTAAATATTGGCCAGAGACCGATAGCGCACAAGTAGAGTGATC-------GAAAGATGAAAAGCACTTTGAAAAGAGGGTTAAACAGCACGTGAAATTGTTGAAAGGGAAGCGCTTGTGACCAGACTTGAGCGCGGCGGATCATCCGGTGTTCTCACCGGTGCACTCCGCCGCGCCCAGGCCAGCATCGGTTTCCGCGGGGGGACAAAGGTCCCGGGAACGTAGCTCCTCCGGGAGTGTTATAGCCCGGGGCGCAATGCCCCCGCGGGGACCGAGGACCGCGCATCTGCAAGGATGCTGGCGTAATGGTCATCAGCGACCCGTCTTGAAACACGGACCAAGGAGTCAAGGTTTTGCGCGAGTGTTTGGGTGTTAAACCCGCACGCGTAATGAAAGTGAA-CGTAGGTGAGAG----CTTCGGCGCATCATCGACCGATCCTGATGTATTCGGATGGATTTGAGTAGGAGCGTTAAGCCTTGGACCCGAAAGATGGTGAACTATGCTTGGATAGGGTGAAGCCAGAGGAAACTCTGGTGGAGGCTCGCAGCGGTTCTGACGTGCAAATCGATCGTCAAATC-TGAGCATGGGGGCGAAAGACTAATCGAACCATCTAGTAGCTGGTTACCGCC????????????????????????????????????????????????????????????????????????????????????????????????????????????????????????????????????????????????????????????????????????????????????????????????????????????????????????????????????????????????????????????????????????????????????????????????????????????????????????????????????????????????????????????????????????????????????????????????????????????????????????????????????????????????????????????????????????????????????????????????????????????????????????????????????????????????????????????????????????????????????????????????????????????????????????????????????????????????????????????????????????????????????????????????????????????????????????????????????????????????????????????????????????????????????????????????????????????????????????????????????????????????????????????????????????????????????????????????????????????????????????????????????????????????????????????????????????????????????????????????????????????????????????????????????????????????????????????????????????????????????????????????????????????????????????????????????????????????????????????????????????????????????????????????????????????????????????????????????????????????????????????????????????????????????????????????????????????????????????????????????????????????????????????????????????????????????????????????????????????????????????????????????????????????????????????????????????????????????????????????????????????????????????????????????????????????????????????????????????????????????????????????????????????????????????????????????????????????

>*Schizothecium*_*tetrasporum*_CBS_394_87

ATTACAGAGTTGC---------AAAACTCC--CA--ACCATTGTGAACC-TACCTC--------ACCGTTGCCTCGGCGG------------------GTGGCTCCCAC-------------CCGGGCCGCGCCGG-------CCCCACC---------GGGCCGGCAA-CCCGTCAGAGG-----ACCGC-AACTCTT---AGTCATCA-TTGGCCTCTCTGAGT-AACTTATACAATAAGTCAAAACTTTC-AACAACGGATCTCTTGGTTCTGGCATCGATGAAGAACGCAGCGAAATGCGATACGTAATGTGAATTGCAGATTTCAGTGAATCATCGAATCTTTGAACGCACATTGCGCCCGCCAGTATTCTGGCGGGCATGCCTGTTCGAGCGTCATTTCAA-CCATCAAGCGCAC-CGCTT-GTGTTGGGGCCCTGCGG----CTG-CCGCAGACCCCTAAATCCAGTGGCGGGCTCGTCGT-CGTACCGAGTGCAGTAA--ACATCCTCGCTCAGGGCAC-GCGCCGGGTCTCTTGCCGTGAAA---CACCC--CACATATCAAGGTTGACCTCGGATCAGGTAGGAATACCCGCTGAACTTAAGCATATCA????????????AAAGAAACCAACAGGG-ATTGCCCCAGTAACGGCGAGTGAAGCGGCAACAGCTCAAATTTGAAATCTGGCCTCGG-CCCGAGTTGTAATTTGCAGAGGAAGCTTCTGGTGCGGCGCCGTCCGAGTCTCCTGGAACGGAGCGCCATAGAGGGTGAGAGCCCCGTATGGACGGATGCCAAACCTGTGTGAAGCTCCTTCGACGAGTCGAGTAGTTTGGGAATGCTGCTCTAAATGGGAGGTAAATTCCTTCTAAAGCTAAATATTGGCCAGAGACCGATAGCGCACAAGTAGAGTGATC-------GAAAGATGAAAAGCACTTTGAAAAGAGGGTTAAACAGCACGTGAAATTGTTGAAAGGGAAGCGCTTGTGACCAGACTTGAGCGCGGCGGATCATCCGGTGTTCTCACCGGTGCACTCCGCCGTGCCCAGGCCAGCATCGGCTTCCGCGGGGGGACAAAGGTCCCGGGAACGTAGCTCCTCCGGGAGTGTTATAGCCCGGGGCGCAATGCCCCCGCGGGGGCCGAGGACCGCGCATCTGCAAGGATGCTGGCGTAATGGTCATCAGCGACCCGTCTTGAAACACGGACCAAGGAGTCAAGGTTTTGCGCGAGTGTTTGGGTGTTAAACCCGCACGCGTAATGAAAGTGAA-CGTAGGTGAGAG----CTTCGGCGCATCATCGACCGATCCTGATGTATTCGGATGGATTTGAGTAGGAGCGTTAAGCCTTGGACCCGAAAGATGGTGAACTATGCTTGGATAGGGTGAAGCCAGAGGAAACTCTGGTGGAGGCTCGCAGCGGTTCTGACGTGCAAATCGATCGTCAAATC-TGAGCATGGGGGCGAAAGACTAATCGAACCATCTAGTAGCTGGTTACCGCC????????????????????????????????????????????????????????????????????????????????????????????????????????????????????????????????????????????????????????????????????????????????????????????????????????????????????????????????????????????????????????????????????????????????????????????????????????????????????????????????????????????????????????????????????????????????????????????????????????????????????????????????????????????????????????????????????????????????????????????????????????????????????????????????????????????????????????????????????????????????????????????????????????????????????????????????????????????????????????????????????????????????????????????????????????????????????????????????????????????????????????????????????????????????????????????????????????????????????????????????????????????????????????????????????????????????????????????????????????????????????????????????????????????????????????????????????????????????????????????????????????????????????????????????????????????????????????????????????????????????????????????????????????????????????????????????????????????????????????????????????????????????????????????????????????????????????????????????????????????????????????????????????????????????????????????????????????????????????????????????????????????????????????????????????????????????????????????????????????????????????????????????????????????????????????????????????????????????????????????????????????????????????????????????????????????????????????????????????????????????????????????????????????????????????????????????????????????????

>*Schizothecium*_*keniense*_YMF175

ATTACAGAGTTGC---------AAAACTCC--CA--ACCATTGTGAACT-TACCTA--------AACGTTGCTTCGGCGG------------------GTGGCGCCCCTAM--------CTCGGGCGCCGCGCCGGGAAC---CCCTAGC-------GGGCCCCGGCAA-CCCGTCAGAAG-----ACTTT-AACTCTT---AGTAACTA-CTGGCCTCTCYGAGT-AACTTATACAATAAGTCAAAACTTTC-AACAACGGATCTCTTGGTTCTGGCATCGATGAAGAACGCAGCGAAATGCGATACGTAATGTGAATTGCAGATTTCAGTGAATCATCGAATCTTTGAACGCACATTGCGCCCGCCAGTATTCTGGCGGGCATGCCTGTTCGAGCGTCATTTCAA-CCATCAAGC-CCC-GGCTT-GTGTTGGAGCCCTGCGG----CTG-CCGCAGACTCCCAAATCCAGTGGCGGGCTCGTCGT-CGTACCGAGTGCAGTAA--ACATCCTCGCTCAGGGAAC-GCGTCGGGT-TCTTGCCGTGAAA---CCCCC--ACTATATCAAGGTTGACCTCGGATCAGGTAGGAATACCCGCTGAACTTAAGCATATCAATAAGCGGAGGAAAAGAAACCAACAGGG-ATTGCCCCAGTAACGGCGAGTGAAGCGGCAACAGCTCAAATTTGAAATCTGGCCTCGG-CCCGAGTTGTAATTTGTAGAGGAAGCTTCTGGTGCGGCGCCGTCCGAGTCTCCTGGAACGGAGCGCCATAGAGGGTGAGAGCCCCGTATGGACGGATGCCAAACCTGTGTGAAGCTCCTTCGACGAGTCGAGTAGTTTGGGAATGCTGCTCTAAATGGGAGGTAAATTCCTTCTAAAGCTAAATATTGGCCAGAGACCGATAGCGCACAAGTAGAGTGATC-------GAAAGATGAAAAGCACTTTGAAAAGAGGGTTAAACAGCACGTGAAATTGTTGAAAGGGAAGCGCTTGTGACCAGACTTGAGCGCGGCGGATCATCCGGTGTTCTCACCGGTGCACTCTGCCGCGCCCAGGCCAGCATCGGTTTCCGCTGGGGGACAAAGGTCCCGGGAACGTAGCTCCTCCGGGAGTGTTATAGCCCGGGGCGTAATGCCCTAGTGGGGACCGAGGACCGCGCATCTGCAAGGATGCTGGCGTAATGGTCATCAGCGACCCGTCTTGAAACACGGACCAAGGAGTCAAGGTTTTGCGCGAGTGTTTGGGTGTTAAACCCGCACGCGTAATGAAAGTGAA-CGTAGGTGAGAG----CTTCGGCGCATCATCGACCGATCCTGATGTATTCGGATGGATTTGAGTAGGAGCGTTAAGCCTTGGACCCGAAAGATGGTGAACTATGCTTGGATAGGGTGAAGCCAGAGGAAACTCTGGTGGAGGCTCGCAGCGGTTCTGACGTGCAAATCGATCGTCAAATC-TGAGCATGGGGGCGAAAGACTAATCGAACCATCTAGTAGCTGGTTACCGCC??????????????????????????????????????????????????GGCGAGCTCGACGGCCGGTGTGTCTCAGGTGTTGAACCGCTACACATTTGCCTCGACGCTCTCCCATTTGCGCCGTACCAACACCCCCATCGGTCGCGACGGAAAGCTCGCCAAGCCGCGCCAGCTTCACAACACACATTGGGGTCTCGTCTGTCCGGCCGAGACGCCCGAGGGCCAGGCCTGCGGGCTGGTCAAGAATCTGTCGCTCATGTGCTACATCAGCGTGGGCACCAACGCGGAGCCCATCATCGAGTTCATGATTGCGCGCAACATGGAGGTCTTGGAGGAGTACGAGCCGCTTCGGTCCCCCAACGCCACCAAGATCTTTGTCAACGGCACCTGGGTCGGCGTGCACCATGACGCCAAGCACCTTGTGCGTCTTGTCCAGGACCTCAGGCGATCGAGCATTGTGAGCTTTGAGGTTTCCCTGGTTCGTGATATCAGAGATCGCGAGTTCAAGATCATGTCGGATGCCGGGCGCGTTATGAGGCCCCTCTTTGTTGTTGAGACAGAAGACGAGAACTCG---------TCGGGGGTGGAAAAGGGCCAGCTCGTGCTCACAAAGACGCACATCCAAAAGCTGGCCAACGACAAGCTGATTGGGAAATACCACAAGGACTACTTTGGGTGGCAGGGTCTCTTGCGGTCTGGCGCCGTCGAATACCTCGACGCCGAGGAAGAGGAGACGACCATGATCTCCATGTCCCCCGAGGACTTGGACCACTTCCGCGAGGCCAAGGC------------GCGGAACTTTGAGGATTCCGAGGTCAAGGTCGAGGGCAACAAGCGTATCCCGACGAGGATCAACCCAACGACGCACATGTACACGCACTGCGAGATTCACCCCAGCATGCTGCTTGGAATCTGCGCCAGCATCATCCCTTTCCCAGACCACAACCAGGCTTGTCGACCAGGTTCTCGATGTCGTCCGTCGCGAGGCCGAGGGCTGCGACTGCCTCCAGGGCTTCCAGATCACTCACTCGCTCGGTGGTGGTACCGGTGCCGGTATGGGTACCCTCCTCATCTCCAAGATCCGCGAGGAGTTCCCCGACCGCATGATGGCGACCTTCTCCGTCGTTCCCTCGCCCAAGGTGTCGGATACCGTCGTCGAGCCCTACAACGCCACCCTCTCCGTCCACCAGCTTGTCGAGAACTCGGACGAGACCTTCTGCATTGACAACGAGGCTCTCTACGACATCTGCATGCGCACCCTCAAGCTGTCGAACCCCTCGTACGGCGACCTCAACCACCTCGTCTCGGCCGTCATGTCGGGTGTCACCGTTTCGCTGCGCTTCCCCGGCCAGCTCAACTCCGATCTCCGCAAGCTCGCCGTCAACATGGTTCCCTTCCCCCGTCTCCACTTCTTCATGGTCGGCTTCGCTCCCCTCACCAGCCGCGGCGCCCACTCCTTCCGTGCCGTCTCGGTTCCCGAGCTCACGCAGCAAATGTTCGACCCCAAGAACATGATGGCTGCCTCTGACTTCCGCAACGGTCGCTACCTCACGTGCTCTGCCATCTT

>*Schizothecium*_*miniglutinans*_CBS_131.94

ATTACAGAGTTGC---------AAAACTCC--CA--ACCATTGTGAATC-TACCTC--------ACCGTTGCTTCGGCGG------------------GTGGCCCGGGGCA-------ACCCCCGGGCCGCGCCGG-------CTCCACT---------GGGCCGGCAA-CCCGTCAGAGG-----ACCGA-AACTCTT---AGTCATCA-TTGGCCTCTCTGAGT-AACTTATACAATAAGTCAAAACTTTC-AACAACGGATCTCTTGGTTCTGGCATCGATGAAGAACGCAGCGAAATGCGATACGTAATGTGAATTGCAGATTTCAGTGAATCATCGAATCTTTGAACGCACATTGCGCCCGCCAGTATTCTGGCGGGCATGCCTGTTCGAGCGTCATTTCAA-CCATCAAGCGCCTGCGCTT-GTGTTGGAGCCCTGCGG----CTG-CCGCAGGCTCCCAAACCCAGTGGCGGGCTCGTCGT-CGTACCGAGTGCAGTAA-ACTTACCACGCTCAGGGAAC-GCGACGGGTGACCGGCCGTAAAA---CCCCC-AAACTTATCAAGGTTGACCTCGGATCAGGTAGGAATACCCGCTGAACTTAAGCATATCAATAAGCGG??????????????????????????????????????????????????????????????????????????????????????????????????????????????????????????????????????????????????????????????????????????????????????????????????????????????????????????????????????????????????????????????????????????????????????????????????????????????????????????????????????????????????????????????????????????????????????????????????????????????????????????????????????????????????????????????????????????????????????????????????????????????????????????????????????????????????????????????????????????????????????????????????????????????????????????????????????????????????????????????????????????????????????????????????????????????????????????????????????????????????????????????????????????????????????????????????????????????????????????????????????????????????????????????????????????????????????????????????????????????????????????????????????????????????????????????????????????????????????????????????????????????????????????????????????????????????????????????????????????????????????????????????????????????????????????????????????????????????????????????????????????????????????????????????????????????????????????????????????????????????????????????????????????????????????????????????????????????????????????????????????????????????????????????????????????????????????????????????????????????????????????????????????????????????????????????????????????????????????????????????????????????????????????????????????????????????????????????????????????????????????????????????????????????????????????????????????????????????????????????????????????????????????????????????????????????????????????????????????????????????????????????????????????????????????????????????????????????????????????????????????????????????????????????????????????????????????????????????????????????????????????????????????????????????????????????????????????????????????????????????????????????????????????????????????????????????????????????????????????????????????????????????????????????????????????????????????????????????????????????????????????????????????????????????????????????????????????????????????????????????????????????????????????????????????????????????????????????????????????????????????????????????????????????????????????????????????????????????????????????????????????????????????????????????????????????????????????????????????????????????????????????????????????????????????????????????????????????????????????

>*Schizothecium*_*curvisporum*_CBS_507_50

ATTACAGAGTTGC---------AAAACTCC--CA--ACCATTGTGAACC-TACCTC--------ACCGTTGCCTCGGCGG------------------GTGGCCCCCAC-------------CAGGGCCGCGCCGG-------CCCCACC----------GGCCGGCAA-CCCGTCAGAGG-----ACCGC-AACTCTT---AGTCATCA-TTGGCCTCTCTGAGT-AACTTATACAATAAGTCAAAACTTTC-AACAACGGATCTCTTGGTTCTGGCATCGATGAAGAACGCAGCGAAATGCGATACGTAATGTGAATTGCAGATTTCAGTGAATCATCGAATCTTTGAACGCACATTGCGCCCGCCAGTATTCTGGCGGGCATGCCTGTTCGAGCGTCATTTCAA-CCATCAAGCGCCC-CGCTT-GTGTTGGGGCCCTGCGG----CTG-CCGCAGACCCCTAAATCCAGTGGCGGGCTCGTCGT-CGTACCGAGTGCAGTAA--ACATCCTCGCTCAGGGCAC-GCGCCGGGTCTCTTGCCGTGAAA---CACCC--CACATATCAAGGTTGACCTCGGATCAGGTAGGAATACCCGCTGAACTTAAGCATATCAATAAGCGGAGGAA?????????ACAGGG-ATTGCCCCAGTAACGGCGAGTGAAGCGGCAACAGCTCAAATTTGAAATCTGGCCTCGG-CCCGAGTTGTAATTTGCAGAGGAAGCTTCTGGTGCGGCGCCGTCCGAGTCTCCTGGAACGGAGCGCCATAGAGGGTGAGAGCCCCGTATGGACGGATGCCAAACCTGTGTGAAGCTCCTTCGACGAGTCGAGTAGTTTGGGAATGCTGCTCTAAATGGGAGGTAAATTCCTTCTAAAGCTAAATATTGGCCAGAGACCGATAGCGCACAAGTAGAGTGATC-------GAAAGATGAAAAGCACTTTGAAAAGAGGGTTAAACAGCACGTGAAATTGTTGAAAGGGAAGCGCTTGTGACCAGACTTGAGCGCGGCGGATCATCCGGTGTTCTCACCGGTGCACTCCGCCGCGCCCAGGCCAGCATCGGCTTCCGCGGGGGGACAAAGGTCCCGGGAACGTAGCTCCTCCGGGAGTGTTATAGCCCGGGGCGCAATGCCCCCGCGGGGGCCGAGGACCGCGCATCTGCAAGGATGCTGGCGTAATGGTCATCAGCGACCCGTCTTGAAACACGGACCAAGGAGTCAAGGTTTTGCGCGAGTGTTTGGGTGTTAAACCCGCACGCGTAATGAAAGTGAA-CGTAGGTGAGAG----CTTCGGCGCATCATCGACCGATCCTGATGTTTTCGGATGGATTTGAGTAGGAGCGTTAAGCCTTGGACCCGAAAGATGGTGAACTATGCTTGGATAGGGTGAAGCCAGAGGAAACTCTGGTGGAGGCTCGCAGCGGTTCTGACGTGCAAATCGATCGTCAAATC-TGAGCATGGGGGCGAAAGACTAATCG?????????????????????????????????????????????????????????????????????????????????????????????????????????????????????????????????????????????????????????????????????????????????????????????????????????????????????????????????????????????????????????????????????????????????????????????????????????????????????????????????????????????????????????????????????????????????????????????????????????????????????????????????????????????????????????????????????????????????????????????????????????????????????????????????????????????????????????????????????????????????????????????????????????????????????????????????????????????????????????????????????????????????????????????????????????????????????????????????????????????????????????????????????????????????????????????????????????????????????????????????????????????????????????????????????????????????????????????????????????????????????????????????????????????????????????????????????????????????????????????????????????????????????????????????????????????????????????????????????????????????????????????????????????????????????????????????????????????????????????????????????????????????????????????????????????????????????????????????????????????????????????????????????????????????????????????????????????????????????????????????????????????????????????????????????????????????????????????????????????????????????????????????????????????????????????????????????????????????????????????????????????????????????????????????????????????????????????????????????????????????????????????????????????????????????????????????????????????????????????????????????????

>*Schizothecium*_*fimbriatum*_CBS_144_54

ATTACAGAGTTGC---------AAAACTCC--CA--ACCATTGTGAACC-TACCTC--------ACCGTTGCTTCGGCGG------------------GTGGCCCCACCC--------------GGGCCGCGTCGG--------CCCCCC--------GGGGCCGGCAA-CCCGTCAGAGG-----ACCCA-CACTCTC---AGTTATCA-TTGGCCTCTCTGAGT-AACTTATACAATAAGTCAAAACTTTC-AACAACGGATCTCTTGGTTCTGGCATCGATGAAGAACGCAGCGAAATGCGATACGTAATGTGAATTGCAGATTTCAGTGAATCATCGAATCTTTGAACGCACATTGCGCCCGCCAGTATTCTGGCGGGCATGCCTGTTCGAGCGTCATTTCAA-CCATCAAGCCCCC-GGCTT-GTGTTGGGGCCCTGCGG----CTG-CCGCAGACCCCTAAATCCAGTGGCGGGCTCGTCGT-CGTACCGAGTGCAGTAA--ACATCCTCGCTCAGGGAAC-CCGACGGGT-GCCGGCCGTGAAA---CCCCC---CCTTCTCAAGGTTGACCTCGGATCAGGTAGGAATACCCGCTGAACTTAAGCATATCAATAAGCGGAGGA???????CCAACAGGGCATTGCCCCAGTAACGGCGAGTGAAGCGGCAACAGCTCAAATTTGAAATCTGGCCTCGG-CCCGAGTTGTAATTTGCAGAGGAAGCTTCTGGTGCGGCGCCGTCCGAGTCTCCTGGAACGGAGCGCCATAGAGGGTGAGAGCCCCGTATGGACGGACGCCAAACCTGTGTGAAGCTCCTTCGACGAGTCGAGTAGTTTGGGAATGCTGCTCAAAATGGGAGGTAAATTCCTTCTAAAGCTAAATATTGGCCAGAGACCGATAGCGCACAAGTAGAGTGATC-------GAAAGATGAAAAGCACTTTGAAAAGAGGGTTAAACAGCACGTGAAATTGTTGAAAGGGAAGCGCTTGTGACCAGACTTGGGCGCGGCGGATCATCCGGTGTTCTCACCGGTGCACTCCGCCGCGCCCGGGCCAGCATCGGCTTCCGCCGGGGGACAAAGGTCCCGGGAACGTAGCTCCTCCGGGAGTGTTATAGCCCGGGGCGCAATGCCCCGGCGGGGGCCGAGGACCGCGCAT-TGCAAGGATGCTGGCGTAATGGTCATCAGCGACCCGTCTTGAAACACGGACCAAGGAGTCAAGGTTTTGCGCGAGTGTTTGGGTGTTAAACCCGCACGCGTAATGAAAGTGAA-CGTAGGTGAGAG----CTTCGGCGCATCATCGACCGATCCTGATGTATTCGGATGGATTTGAGTAGGAGCGTTAAGCCTTGGACCCGAAAGATGGTGAACTATGCTTGGATAGGGTGAAGCCAGAGGAAACTCTGGTGGAGGCTCGCAGCGGTTCTGACGTGCAAATCGATCGTCAAATC-TGAGCATGGGGGCGAAAGACTAATCGA????????????????????????GCGCTCAAGTACTCGCTTGCCACGGGCAACTGGGGCGACCAGAAGAAGGCGGCAAGCTCGACGGCCGGCGTGTCGCAGGTGCTGAATCGCTACACGTTCGCCTCGACCCTCTCTCATTTGCGCCGCACCAACACACCCATCGGTCGCGATGGAAAGCTCGCCAAGCCGCGTCAGCTTCACAACACGCATTGGGGCCTCGTCTGTCCGGCCGAGACGCCCGAGGGCCAGGCCTGCGGGCTGGTCAAGAATCTGTCGCTCATGTGCTACATCAGCGTGGGCACCAACGCGGAGCCCATTGTCGAGTTCATGATTGCGCGCAACATGGAGGTGTTGGAAGAGTACGAGCCGCTGCGCTCCCCCAACGCCACCAAGATCTTTGTCAACGGCACATGGGTCGGCGTGCACCACGACGCCAAGCACCTCGTGCACCTTGTCCAGGGCCTCCGGCGATCCAACATTGTGAGCTTCGAGGTGTCGCTGGTCCGGGATATCCGAGACCGCGAGTTCAAGATCATGTCGGATGCCGGCCGCGTCATGAGACCGCTCTTTGTTGTCGAGACCGAGGACGAGAGCTCT---------ACGGGAGTGGAAAAGGGCGAGCTGGTGCTCACCAAGACCCACGTCCAGAAGCTGGCCAACGACAAGCTGATTGGGAAATACCACAAGGACTACTTTGGGTGGCAAGGCCTCTTGCAATCGGGCGCCGTCGAATACCTCGACGCCGAGGAGGAGGAGACGTCCATGATCTCCATGTCGCCCGAGGACCTGGACCATTTCCGCGACGCCAAGGC------GAGAAACTTTGAGGAGCCCGAGGGCAAGGTGGTGACCGAGGGCAACAAGCGCATCCCGACGAGGATCAACCCGACGACCTACATGTACACGCACTGCGAGATCCACCCCAGCATGCTGCTCGGTATCTGCGCGAGCATCATCCCTTTCCCCGACCACAACCAGGCTTGTCGACCAGGTTCTCGATGTCGTCCGTCGCGAGGCCGAGGGCTGCGACTGCCTCCAGGGCTTCCAGATCACCCACTCGCTCGGTGGTGGTACCGGTGCCGGCATGGGTACTCTCCTCATCTCCAAGATCCGCGAGGAGTTCCCCGACCGCATGATGGCGACCTTCTCCGTCGTCCCCTCGCCCAAGGTGTCGGATACCGTCGTGGAACCCTACAACGCCACCCTCTCCGTCCACCAGCTTGTTGAGAACTCGGACGAGACCTTCTGCATTGACAACGAGGCTCTCTACGACATCTGCATGCGCACCCTCAAGCTGTCGAACCCCTCGTACGGCGACCTCAACCACCTCGTCTCGGCCGTCATGTCGGGTGTCACCGTTTCGCTGCGCTTCCCCGGCCAGCTCAACTCCGATCTCCGCAAGCTCGCCGTGAACATGGTTCCCTTCCCCCGTCTCCACTTCTTCATGGTCGGCTTCGCCCCTCTTACCAGCCGTGGCGCCCACTCTTTCCGCGCCGTTTCGGTTCCCGAGCTCACCCAGCAGATGTTCGACCCCAAGAACATGATGGCTGCCTCGGACTTCCGCAACGGTCGTTACCTTACCTGCTCTGCCATCTT

>*Schizothecium_dactylidis*_KUNCC_25-1919

ATTACAGAGTTGC---------AAAACTCC--CA--ACCATTGTGAATC-TACCTC--------ACCGTTGCCTCGGCGG------------------GTGGCTCCTCCCCCTCACCCGGGGTCGGGCCGCGCCGG-------CTCCCGGCT-CCC-GGGCGCCGGCAATCCCGTCAGAGG-----ACCAGCAACCTTC---TGTCGTCA-TTGGCCTCTCTGAGT-AACTTATACAATAAGTCAAAACTTTC-AACAACGGATCTCTTGGTTCTGGCATCGATGAAGAACGCAGCGAAATGCGATACGTAATGTGAATTGCAGATTTCAGTGAATCATCGAATCTTTGAACGCACATTGCGCCCGCCAGTATTCTGGCGGGCATGCCTGTTCGAGCGTCATTTCAA-CCATCAAGCGCCCGCGCTT-GTGTTGGAGCCCTGCGG----CCG-CCGCAGCCTCCCAAACCCAGTGGCGGGCTCGTCGT-CGTACCGAGTGCAGTAA-ACATACCACGCTCCGGGCAC-GCGACGGGTCACCGGCCGTTAAA---CCCCCC--ACTTATCAAGGTTGACCTCGGATCAGGTAGGAATACCCGCTGAACTTAAGCATATCAA??????????????????????CAGGG-ATTGCCCCAGTAACGGCGAGTGAAGCGGCAACAGCTCAAATTTGAAATCTGGCCTCGG-CCCGAGTTGTAATTTGCAGAGGAAGCTTCTGGTGCGGCGCCGTCCGAGTCTCCTGGAACGGAGCGCCATAGAGGGTGAGAGCCCCGTATGGACGGATGCCAAACCTGTGTGAAGCTCCTTCGACGAGTCGAGTAGTTTGGGAATGCTGCTCAAAATGGGAGGTAAATTCCTTCTAAAGCTAAATATTGGCCAGAGACCGATAGCGCACAAGTAGAGTGATC-------GAAAGATGAAAAGCACTTTGAAAAGAGGGTTAAACAGCACGTGAAATTGTTGAAAGGGAAGCGCTTGTGACCAGACTTGAGCGCGGCGGATCATCCGGTGTTCTCACCGGTGCACTCCGCCGCGCCCAGGCCAGCATCGGTTTCCGCGGGGGGACAAAGGTCCCGGGAACGTGGCTCCTCCGGGAGTGTTATAGCCCGGGGCGCAATGCCCCCGCAGGGACCGAGGACCGCGCATCTGCAAGGATGCTGGCGTAATGGTCATCAGCGACCCGTCTTGAAACACGGACCAAGGAGTCAAGGTTTTGCGCGAGTGTTTGGGTGTTAAACCCGCACGCGTAATGAAAGTGAA-CGTAGGTGAGAG----CTTCGGCGCATCATCGACCGATCCTGATGTATTCGGATGGATTTGAGTAGGAGCGTTAAGCCTTGGACCCGAAAGATGGTGAACTATGCTTGGATAGGGTGAAGCCAGAGGAAACTCTGGTGGAGGCTCGCAGCGGTTCTGACGTGCAAATCGATCGTCAAATC-TGAGCATGGGGGCGAAA??????????????????????????????????GCGCTCAAGTACTCGCTCGCCACCGGCAACTGGGGCGACCAGAAGAAGGCGGCGAGCTCGACGGCCGGCGTGTCCCAAGTCTTGAATCGCTACACGTTTGCCAGTACCCTCTCGCATTTGCGGCGCACCAACACGCCCATCGGCCGCGACGGAAAGCTCGCAAAGCCGCGCCAGCTTCACAACACCCATTGGGGTCTCGTCTGTCCGGCAGAGACGCCCGAGGGCCAGGCTTGCGGGCTGGTCAAGAATCTATCGCTCATGTGCTACATCAGCGTGGGCACCAACGCGGAGCCCATTGTCGAGTTCATGATTGCGCGGAACATGGAGGTCTTGGAAGAGTACGAGCCCTTGCGTTCTCCCAACGCCACCAAGATCTTTGTCAACGGCACATGGGTCGGCGTTCATCAAGACCCGAAACACCTCGTGCACCTGGTTCAGGGACTTAGGCGGTCCAACATTGTGAGCTTCGAGGTGTCGCTCGTGCGGGATATCCGAGACCGTGAATTCAAGATCATGTCGGATGCCGGCCGCGTCATGAGGCCCCTCTTCGTCGTCGAGACGGAGGATGAGAGCTCG---------ACGGGGGTGGAAAAGGGTCAGCTGGTGCTCACCAAAGCCCACATCCAAAAGCTGGCCAACGACAAACTGATTGGGAAATACCACAAGGACTACTTTGGGTGGCAGGGCCTGTTGCAGTCGGGCGCCGTCGAATACCTCGACGCAGAGGAGGAGGAGACGGCCATGATC???????????????????????????????????????????????????????????????????????????????????????????????????????????????????????????????????????????????????????????????????????????????????????????????????????????????????????????????????????????????????????????????????????????????????????????????????????????????????????????????????????????????????????????????????????????????????????????????????????????????????????????????????????????????????????????????????????????????????????????????????????????????????????????????????????????????????????????????????????????????????????????????????????????????????????????????????????????????????????????????????????????????????????????????????????????????????????????????????????????????????????????????????????????????????????????????????????????????????????????????????????????????????????????????

>*Schizothecium*_*curvuloides*_CBS_129.94

ATTACAGAGTTGC---------AAAACTCC--CA--ACCATTGTGAACA-TACCTC--------ACCGTTGCTTCGGCGG------------------GTGGCCCCACTCCCTCACCCGGGGTGGGGCCGCGCCGG--------CCCCGGCTTCCC-GGGCGCCGGCAATCCCGTCAAAGG-----ACCTG-AACTCTT---AGTCATCA-TTGGCCTCTCTGAGT-AACTTATACAATAAGTCAAAACTTTC-AACAACGGATCTCTTGGTTCTGGCATCGATGAAGAACGCAGCGAAATGCGATACGTAATGTGAATTGCAGATTTCAGTGAATCATCGAATCTTTGAACGCACATTGCGCCCGCCAGTATTCTGGCGGGCATGCCTGTTCGAGCGTCATTTCAA-CCATCAAGCGCCCGCGCTT-GTGTTGGAGCCCTGCGG----CCG-CCGCAGGCTCCCAAACCCAGTGGCGGGCTCGTCGT-CGTACCGAGTGCAGTAA-ACATACCACGCTCAGGGCAC-GCGGCGGGTGACCGGCCGTAAAA---CCCCCC--ACATATCAAGGTTGACCTCGGATCAGGTAGGAATACCCGCTGAACTTAAGCATATCAATAAGCGG??????????????????????????????????????????????????????????????????????????????????????????????????????????????????????????????????????????????????????????????????????????????????????????????????????????????????????????????????????????????????????????????????????????????????????????????????????????????????????????????????????????????????????????????????????????????????????????????????????????????????????????????????????????????????????????????????????????????????????????????????????????????????????????????????????????????????????????????????????????????????????????????????????????????????????????????????????????????????????????????????????????????????????????????????????????????????????????????????????????????????????????????????????????????????????????????????????????????????????????????????????????????????????????????????????????????????????????????????????????????????????????????????????????????????????????????????????????????????????????????????????????????????????????????????????????????????????????????????????????????????????????????????????????????????????????????????????????????????????????????????????????????????????????????????????????????????????????????????????????????????????????????????????????????????????????????????????????????????????????????????????????????????????????????????????????????????????????????????????????????????????????????????????????????????????????????????????????????????????????????????????????????????????????????????????????????????????????????????????????????????????????????????????????????????????????????????????????????????????????????????????????????????????????????????????????????????????????????????????????????????????????????????????????????????????????????????????????????????????????????????????????????????????????????????????????????????????????????????????????????????????????????????????????????????????????????????????????????????????????????????????????????????????????????????????????????????????????????????????????????????????????????????????????????????????????????????????????????????????????????????????????????????????????????????????????????????????????????????????????????????????????????????????????????????????????????????????????????????????????????????????????????????????????????????????????????????????????????????????????????????????????????????????????????????????????????????????????????????????????????????????????????????????????????????????????????????????????????????????????

>*Schizothecium*_*dakotense*_CBS_130.94

ATTACAGAGTTGC---------AAAACTCC--CA--ACCATTGTGAATC-TACCTC--------ACCGTTGCCTCGGCGG------------------GTGGCTCCTCCCCCTCACCCGGGGTCGGGCCGCGCCGG-------CTCCCGGCT-CCC-GGGCGCCGGCAATCCCGTCAGAGG-----ACCAGCAACCTTC---TGTCGTCA-TTGGCCTCTCTGAGT-AACTTATACAATAAGTCAAAACTTTC-AACAACGGATCTCTTGGTTCTGGCATCGATGAAGAACGCAGCGAAATGCGATACGTAATGTGAATTGCAGATTTCAGTGAATCATCGAATCTTTGAACGCACATTGCGCCCGCCAGTATTCTGGCGGGCATGCCTGTTCGAGCGTCATTTCAA-CCATCAAGCGCCCGCGCTT-GTGTTGGAGCCCTGCGG----CCG-CCGCAGCCTCCCAAACCCAGTGGCGGGCTCGTCGT-CGTACCGAGTGCAGTAA-ACATACCACGCTCCGGGCAC-GCGACGGGTCACCGGCCGTTAAA---CCCCCC--ACTTATCAAGGTTGACCTCGGATCAGGTAGGAATACCCGCTGAACTTAAGCATATCAATAAGCGG??????????????????????????????????????????????????????????????????????????????????????????????????????????????????????????????????????????????????????????????????????????????????????????????????????????????????????????????????????????????????????????????????????????????????????????????????????????????????????????????????????????????????????????????????????????????????????????????????????????????????????????????????????????????????????????????????????????????????????????????????????????????????????????????????????????????????????????????????????????????????????????????????????????????????????????????????????????????????????????????????????????????????????????????????????????????????????????????????????????????????????????????????????????????????????????????????????????????????????????????????????????????????????????????????????????????????????????????????????????????????????????????????????????????????????????????????????????????????????????????????????????????????????????????????????????????????????????????????????????????????????????????????????????????????????????????????????????????????????????????????????????????????????????????????????????????????????????????????????????????????????????????????????????????????????????????????????????????????????????????????????????????????????????????????????????????????????????????????????????????????????????????????????????????????????????????????????????????????????????????????????????????????????????????????????????????????????????????????????????????????????????????????????????????????????????????????????????????????????????????????????????????????????????????????????????????????????????????????????????????????????????????????????????????????????????????????????????????????????????????????????????????????????????????????????????????????????????????????????????????????????????????????????????????????????????????????????????????????????????????????????????????????????????????????????????????????????????????????????????????????????????????????????????????????????????????????????????????????????????????????????????????????????????????????????????????????????????????????????????????????????????????????????????????????????????????????????????????????????????????????????????????????????????????????????????????????????????????????????????????????????????????????????????????????????????????????????????????????????????????????????????????????????????????????????????????????????????????????????????
